# Genomics of Rapid Incipient Speciation in Sympatric Threespine Stickleback

**DOI:** 10.1371/journal.pgen.1005887

**Published:** 2016-02-29

**Authors:** David A. Marques, Kay Lucek, Joana I. Meier, Salome Mwaiko, Catherine E. Wagner, Laurent Excoffier, Ole Seehausen

**Affiliations:** 1 Aquatic Ecology and Evolution, Institute of Ecology and Evolution, University of Bern, Bern, Switzerland; 2 Department of Fish Ecology and Evolution, Centre of Ecology, Evolution & Biogeochemistry, Eawag: Swiss Federal Institute of Aquatic Science and Technology, Kastanienbaum, Switzerland; 3 Computational and Molecular Population Genetics Lab, Institute of Ecology and Evolution, University of Bern, Bern, Switzerland; 4 Swiss Institute of Bioinformatics, Lausanne, Switzerland; 5 Biodiversity Institute, University of Wyoming, Wyoming, United States of America; 6 Department of Animal and Plant Science, University of Sheffield, Sheffield, United Kingdom; University of California Davis, UNITED STATES

## Abstract

Ecological speciation is the process by which reproductively isolated populations emerge as a consequence of divergent natural or ecologically-mediated sexual selection. Most genomic studies of ecological speciation have investigated allopatric populations, making it difficult to infer reproductive isolation. The few studies on sympatric ecotypes have focused on advanced stages of the speciation process after thousands of generations of divergence. As a consequence, we still do not know what genomic signatures of the early onset of ecological speciation look like. Here, we examined genomic differentiation among migratory lake and resident stream ecotypes of threespine stickleback reproducing in sympatry in one stream, and in parapatry in another stream. Importantly, these ecotypes started diverging less than 150 years ago. We obtained 34,756 SNPs with restriction-site associated DNA sequencing and identified genomic islands of differentiation using a Hidden Markov Model approach. Consistent with incipient ecological speciation, we found significant genomic differentiation between ecotypes both in sympatry and parapatry. Of 19 islands of differentiation resisting gene flow in sympatry, all were also differentiated in parapatry and were thus likely driven by divergent selection among habitats. These islands clustered in quantitative trait loci controlling divergent traits among the ecotypes, many of them concentrated in one region with low to intermediate recombination. Our findings suggest that adaptive genomic differentiation at many genetic loci can arise and persist in sympatry at the very early stage of ecotype divergence, and that the genomic architecture of adaptation may facilitate this.

## Author Summary

Ecological speciation can be defined as the evolution of new, reproductively isolated, species driven by natural selection and ecologically-mediated sexual selection. Its genomic signature has mainly been studied in ecotypes and emerging species that started diverging hundreds to thousands of generations ago, while little is known about the very early stages of species divergence. To fill this knowledge gap, we studied whether and how threespine stickleback, which have adapted either to lake or to stream environments in less than 150 years, differ across their genomes. We found several segments of the genome to be clearly divergent between lake and stream ecotypes, even when both forms breed side by side in the same area. Strikingly, this genomic differentiation was mainly concentrated in one region with low to intermediate recombination rates and clustered around genes controlling ecotype-specific phenotypic traits. Our findings suggest that genomic differentiation can arise despite gene flow already very early at the onset of speciation, and that its occurrence may be facilitated by the genomic organization of genes that control traits involved in adaptation and reproductive isolation.

## Introduction

The question of how and why populations split and diverge into new species is foundational to the field of evolutionary biology. Our ability to study the genetic basis of these processes has fundamentally changed with the next-generation sequencing revolution, which for the first time in history allows biologists to study genome-wide changes associated with speciation at the levels of individuals and populations [1]. In particular, speciation driven by divergent natural selection and by ecologically-mediated sexual selection, termed ‘ecological speciation’ [2], has come into the focus of speciation genomics. This is because genomic data allows us to make inferences on the relationship between individual phenotype and genotype, to detect targets of selection and to infer past and present gene flow among emerging species. The influences of gene flow, selection, mating, standing genetic variation, the organization of genes in the genome and of geography on speciation can now be investigated with unprecedented resolution.

Consequently, ecological speciation theory has increasingly explored more complex scenarios incorporating these factors, including predictions about how genome-wide patterns of divergence reflect these processes [3–7]. Genetic differentiation is expected to be heterogeneous across the genome, because loci under disruptive ecological selection, conferring extrinsic post-zygotic reproductive isolation, will be more resistant to gene flow than the rest of the genome, leading to elevated differentiation around these loci [3]. Other barrier loci conferring intrinsic post-zygotic or pre-zygotic reproductive isolation can have similar effects. Collectively, these genomic regions resistant to gene flow have been called ‘genomic islands of differentiation’ [5,8,9]. Such genomic islands are thought to be the points around which reproductive isolation ‘crystallizes’. They are expected to be more effective if they contain several genes involved in adaptation or reproductive isolation with little recombination between them [10–14], for example multiple adapted genes captured inside an inversion [15,16] or close to centromeres [17]. This matters most when speciation happens in the face of considerable gene flow. At the beginning of such speciation, only few islands of differentiation in the genome are expected to be under sufficiently strong divergent selection to resist gene flow [3–6]. Unless the regions under divergent selection also pleiotropically affect mate choice [18,19], gene flow is expected to relatively freely occur across the rest of the genome at this stage. With increasing reproductive isolation, either because some of the selected loci will have effects on mating through linkage or pleiotropy [20], or because selection works on linkage disequilibrium between genomic islands [21], the number of islands is predicted to increase and the rest of the genome should start diverging due to background selection, selection unrelated to speciation and due to drift. Some models predict further that islands would grow in size due to a local ‘spill over’ effect of strong selection reducing effective gene flow at nearby, weakly selected mutations [5,22].

### Controversial origins of genomic islands

Several empirical studies have looked for such patterns in divergently adapted ecotypes, incipient species and incompletely isolated species with varying degrees of reproductive isolation [23–30]. Most of them have revealed heterogeneous genomic differentiation across genomes with islands of differentiation among ecotypes or species [8,23,24,26–29,31–36]. While some studies found mainly many smaller islands of differentiation [24,26,28–30,32,33,35,36], others found few large islands [8,27], and in some cases islands were associated with genomic regions of reduced recombination, e.g. inside inversions [8,26,37]. Most authors have interpreted these patterns as evidence for ongoing differential gene flow among incipient species, concluding that speciation with gene flow might be common [e.g. 8,24,27,28,34,38]. However, this conclusion has been challenged as some of the observed patterns of genomic differentiation might equally be explained by speciation without gene flow [39,40]. Indeed, when allopatric populations have no gene flow, heterogeneous differentiation across the genome is also expected due to local adaptation, background selection and drift in each population interacting with variation in recombination and mutation rates [39–41]. Therefore, sympatric species that began to speciate in allopatry before they established sympatry can also show this pattern.

In order to find genomic signatures of speciation with gene flow, it is therefore crucial to distinguish between different possible causes of heterogeneous genomic divergence. One way to address this is to investigate pairs of populations with independent evidence for current gene flow and where a phase of geographical isolation can be ruled out. This is difficult for ecotypes or species for which divergence started several thousands to millions of generations ago [42], as in most current speciation genomic studies. Instead, a focus on the very beginning of the ecological speciation process, when recently emerged ecotypes have diverged for tens to a few hundreds of generations without geographical isolation, does minimize uncertainty about past and current gene flow. It has the caveat, though, that it is impossible to know whether the ecotypes will continue to evolve towards distinct species and ultimately build diversity at macroevolutionary scales [1]. We here study very recently diverged ecotypes of the threespine stickleback (*Gasterosteus aculeatus* complex) that resemble older ecotypes and reproductively isolated species of this complex that are well-studied elsewhere in the world [43].

### Recent ecotype divergence in Lake Constance threespine stickleback

Threespine stickleback are a popular model for ecological speciation research because ecotypes have repeatedly evolved many times across the Northern hemisphere, by adapting to different habitats and evolving various degrees of reproductive isolation [43]. While most stickleback ecotypes and species pairs started diverging soon after the retreat of the Pleistocene glaciers ~12,000 years ago [43] (but see [44,45]), stickleback were introduced into the Lake Constance region only less than 150 years ago [46]. This date comes from the examination of detailed records on the fish of the Lake Constance region, reaching back several hundred years in time [47–50], and from ichthyologic analyses of the distribution and natural history of stickleback in that region, which all show that stickleback did not exist in the catchment until late in the 19^th^ century [51,52]. A recent analysis suggested that stickleback had been present in the Lake Constance region for at least 2,000 to 4,000 years and had colonized Lake Constance from the upper Danube [53]. This is at odds with historical data that unequivocally document the absence of stickleback from the middle and upper Danube until the 19^th^ century, when stickleback were introduced both into the upper Danube and into the Lake Constance system [46–52,54]. Mitochondrial phylogeographic analyses further suggest that the Lake Constance stickleback population originates from a North Eastern European lineage inhabiting the Southern Baltic Sea catchments [46,55]. It is only around the middle of the 20^th^ century that stickleback have become common in Lake Constance and inflowing rivers [51].

**Fig 1:**
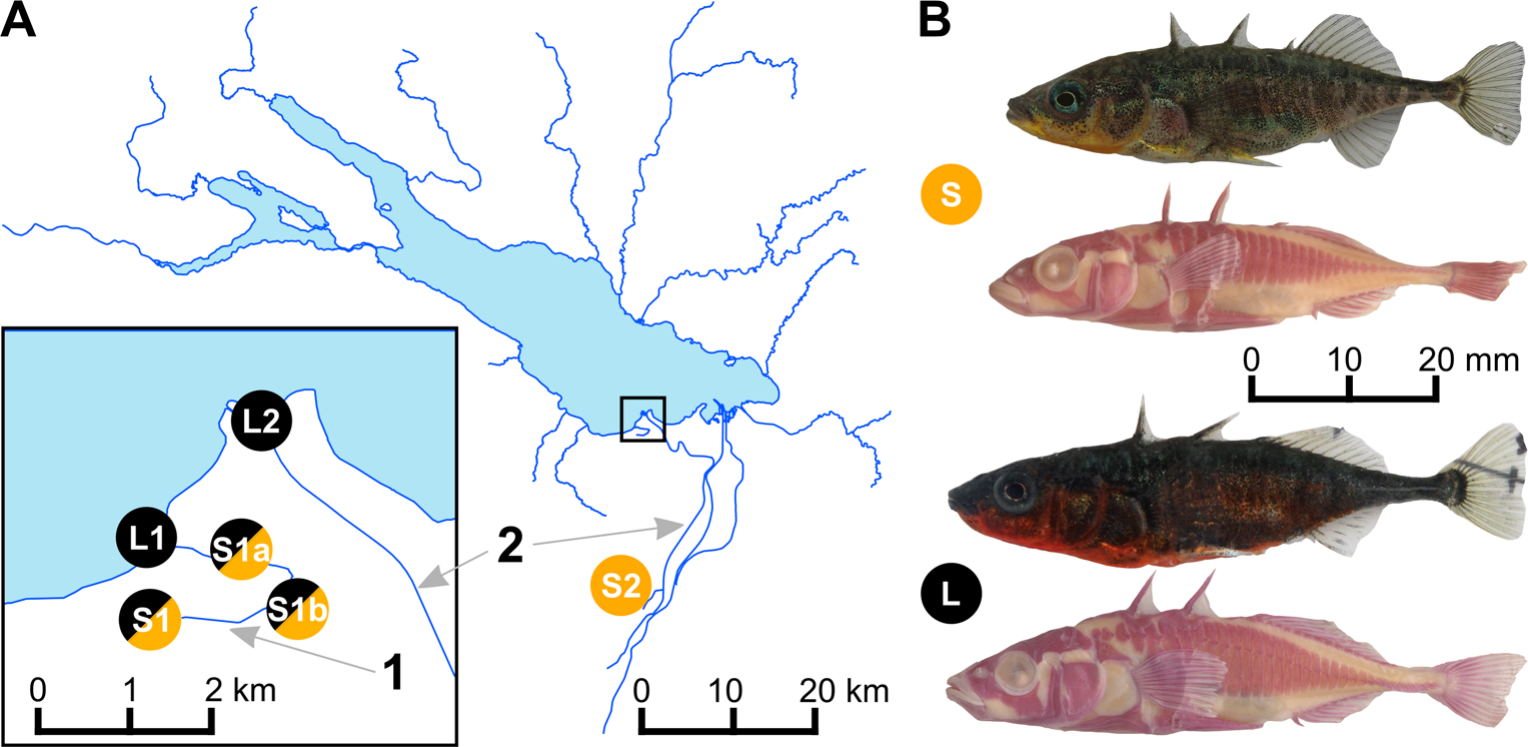
Sampling sites in the Lake Constance area and lake and stream ecotypes of threespine stickleback. **(A)** Map of Lake Constance. In stream 1, both ecotypes breed in sympatry and thus opportunity for gene flow among ecotypes is geographically unconstrained, while in stream 2, ecotypes breed in distant parapatry or effective allopatry, and geographical opportunity for gene flow is therefore strongly restricted. We sampled stickleback early in the breeding season, during the migration of the lake ecotype into streams, before site S1 in stream 1 was reached by lake stickleback, but when both migrant lake and resident stream stickleback were present at intermediate sites S1a and S1b along stream 1. **(B)** Pictures show representative males of both lake (L) and stream (S) ecotypes in full breeding colors, and alizarin-red stained to highlight skeletal features.

Despite the recent colonization of Lake Constance, distinct lake and stream ecotypes have already evolved in this system (cf. Fig 1B, [46,56]). Present day ecotypes differ in predator defense morphology, feeding-related morphology, male nuptial coloration, ecology, growth, and life history [56–59]. Stream stickleback are resident breeders in little streams around Lake Constance, they grow to a smaller adult size, reproduce earlier, die younger, and have shorter spines and smaller bony lateral plates than the lake ecotype [56–59]. Different from all previously studied lake-stream stickleback pairs, however, the lake stickleback that we study in Lake Constance are potamodromous, meaning that in spring they migrate into streams to breed in full sympatry with stream resident stickleback. Adults return to the lake after the breeding season as well as juveniles, where they spend most of their lives before returning to streams only as breeding adults. The adults of these potamodromous lake stickleback have a more pelagic diet than the stream resident fish, differ in feeding-related morphology, including longer gill rakers and a more torpedo-shaped body typical for pelagic fish, have longer spines, and are infested by more and a wider diversity of parasites [56–60].

Whether one of these ecotypes or a population of generalists was initially introduced to the Lake Constance system is unknown. Historically, stickleback were first recorded in isolated stream habitats [48,51]. From there they could have colonized the lake and adapted to this novel habitat before they entered other effluent streams and underwent renewed bouts of adaptation, now again to stream habitats. Ancestral stream stickleback may also have colonized other streams by long distance dispersal through the lake, before they adapted to and colonized the lake environment. Alternatively, as the stickleback populations from Lake Constance and the Eastern effluent streams are closely related to stickleback from catchments South of the Baltic Sea, where freshwater stickleback resemble typical marine stickleback in body armor [61,62], these fish may have been preadapted to living in large lakes with many gape-limited predators and might have adapted to stream habitats only subsequently. Finally, given the presence of other distinct lineages of stickleback in Switzerland and Germany immediately West of Lake Constance [46], it is possible that different sections of the Lake Constance catchment have been colonized independently by different stickleback lineages as is suggested by some phenotypic and genetic data. For instance, mtDNA haplotypes from the distinct Rhine and Rhone lineages of stickleback are abundant in inlet streams of the Northern, Western and South-Western shores of Lake Constance, alongside Baltic haplotypes [56]. Admixture with these Western European populations, which were isolated from Eastern lineages for several ten thousand years in ancient freshwater refugia [63,64] is also suggested by the presence of many fish with reduced body armor in the more Western effluents of Lake Constance [53,56]. In contrast, lake and stream stickleback populations from the South-Eastern effluents of Lake Constance (Fig 1A) that we studied here, have the Baltic mitochondrial haplotype, are predominately fully plated (S7B Fig, [46,59]), are very closely related in microsatellite and AFLP markers [46,55] and show little if any genomic introgression from Rhine and Rhone stickleback populations [55]. Yet they have evolved phenotypically distinctly different lake and stream ecotypes [58,59,65].

Here we study genomic differentiation among these young lake and stream ecotypes in two streams, each containing breeding populations of both resident stream and potamodromous lake ecotypes (Fig 1A). In one long stream, the breeding sites of the ecotypes are separated by many kilometers of less suitable habitat, which likely exceeds within-generation migration abilities of lake stickleback [cf. 56,66], such that this ecotype pair can be considered to breed in effective allopatry or, more conservatively, in distant parapatry. Parapatry or allopatry is typical of all lake and stream stickleback ecotypes studied to date [43,66–69], including previous work on Lake Constance [46,56,59], and also of many marine and freshwater ecotypes [43]. In the other, shorter stream, migratory lake stickleback breed alongside resident stream stickleback in full sympatry (Fig 1A) at the same time of the year (S1 Fig) and lake fish outnumber stream fish in large parts of the stream, providing ample opportunity for interbreeding, and thus potentially allowing high levels of gene flow between ecotypes. We took advantage of the migratory behavior of the lake ecotype and we sampled stickleback at different sites along this stream early in the breeding season, just after the spawning run of the lake ecotype had started and before the most upstream site was reached by lake stickleback. We were thus able to collect both ecotypes separately at the opposite ends of the stream gradient, and also at the same sites in the middle sections of the stream (Fig 1A).

### Frequent parapatry, rare sympatry

Previous population genomic studies of parapatric stickleback ecotypes have shown the presence of parallel genome-wide differentiation between marine and many independently derived freshwater ecotypes from around the Northern hemisphere [24–26,45]. In contrast, almost no genomic parallelism has been found in previous studies that compared parapatric, non-migratory lake and stream ecotypes from different river systems [32,36,70]. A recent natural experiment demonstrated that repeated marine-freshwater differentiation can emerge after only a few decades of adaptation in allopatry [45]. However, whether genomic divergence can emerge in sympatry (or close parapatry) on such a short timescale or be maintained in sympatry after just a few decades of divergence is unknown. The only known sympatric stickleback ecotypes, seven cases of largely reproductively isolated limnetic and benthic lake stickleback species from lakes in British Columbia [43,71], have diverged for a much longer time, several thousand years [25,72], and now show parallel genomic differentiation in sympatry that likely originated from double colonization of these lakes from the same marine source populations [25].

A case of sympatrically breeding lake and stream stickleback ecotypes has not been studied before and should thus, in comparison with a ‘standard’ parapatric contrast that we also investigated, provide insight into the effects of strong versus weak gene flow on the population genomics of ecotype divergence. We identify several regions in the genome that carry divergence islands which are robust to gene flow, suggesting that our sympatrically breeding ecotypes are indeed incipient species and not phenotypically plastic life history morphs. We ask if predictions from ecological speciation with gene flow models hold when we compare lake-stream ecotype pairs in different geographical settings. For instance, to the extent that speciation is constrained by gene flow, we expect lower average genomic differentiation, a smaller number of islands of differentiation and less heterogeneity in genomic differentiation in the sympatric than in the parapatric contrast. Furthermore, we predict that parallel divergent selection across multiple habitat transitions (i.e. between the lake and these two streams), acting on similar initial standing genetic variation present in the colonizing lineage, should lead to an overlap between the genomic islands of differentiation in both streams. Independent of what phenotype was ancestral and in what direction colonization of habitats happened (i.e. a transition first from a stream to a lake population followed by transition back from the lake to other streams, or multiple transitions from a lake population to different stream populations), such parallel genomic islands should reveal genomic regions under habitat-driven divergent selection. Our findings shed light on the interactions of divergent selection, gene flow, standing genetic variation and genomic organization at the earliest stage of ecological speciation.

## Results

### Genomic variation and differentiation

**Fig 2:**
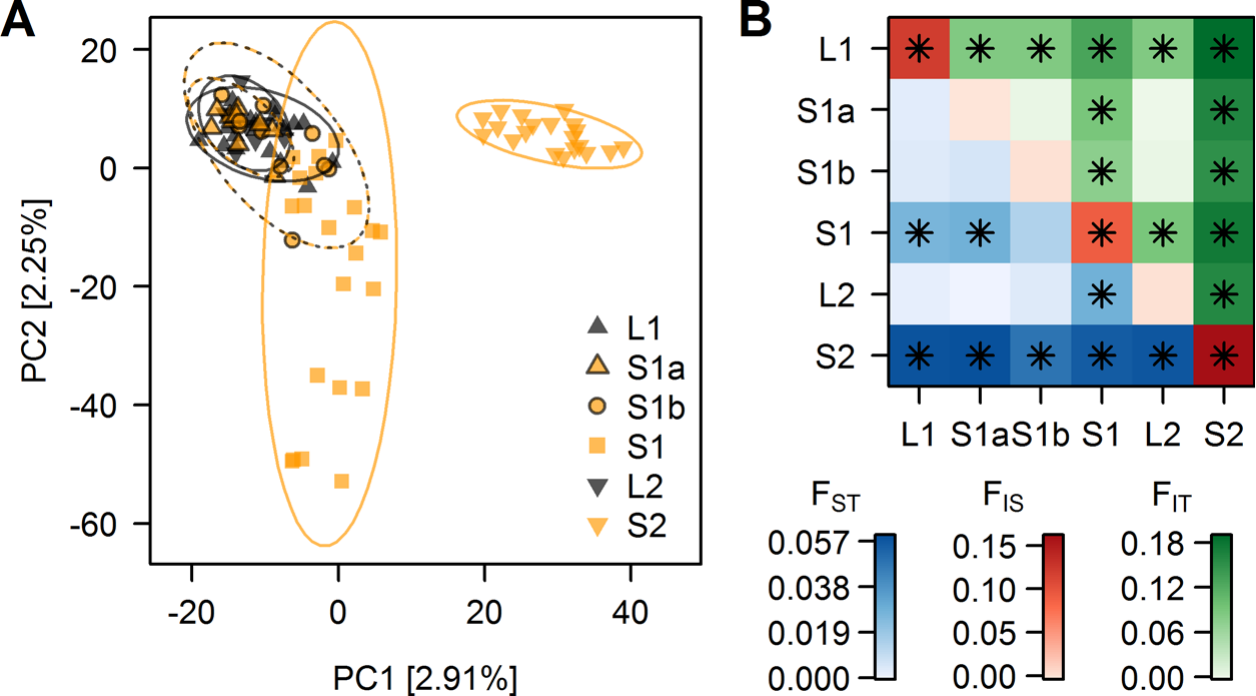
Genomic variation within and average differentiation between sampling sites. **(A)** Principal component analysis: PC1 separates site S2 individuals from the rest, while PC2 separates S1 individuals from the rest. Fill colors indicate the habitat in which individuals were caught, four stream habitat sites (orange) and two lake shore sites (black). PC analysis is based on the 24,784 SNP allele dataset with minor allele frequency >1%. **(B)** F-statistics: between sampling sites pairwise weighted average F_ST_ and F_IT_-statistics are shown below and above the diagonal respectively, F_IS_ for each sampling site on the diagonal. Stars indicate values significantly different from zero (permutation test, >16,000 permutations, p<0.001). F-statistics are based on the 34,756 SNP genotype dataset.

We sequenced restriction-site associated DNA (RAD) tags of 91 threespine stickleback collected at six sites along the two streams flowing into Lake Constance and at their inlets into the lake (Fig 1A, Table 1). After filtering for high-quality genotypes (see Materials and Methods), we obtained a genotype dataset of 3,183,890 bp nuclear DNA sequence containing 34,756 bi-allelic SNPs, including 15,092 SNPs with minor allele frequency greater than 1% at an average sequencing depth per individual ranging between 43 and 148x. We noticed increased mean F_IS_ estimates in populations L1, S1 and S2 (Fig 2B), suggesting an excess of homozygotes in these populations. This could be due to real inbreeding, but it is more likely caused by the presence of PCR duplicates (see Material and Methods) leading to an excess of apparently homozygous genotypes, a well-known feature of single-end RAD tag sequencing [73,74] mimicking inbreeding, and thus effectively reducing the number of sampled chromosomes [75]. We accounted for this excess of homozygotes by allowing for inbreeding in the estimation of F-statistics, and by explicitly incorporating F_IS_ estimates in the detection of outlier loci (see Material and Methods). Furthermore, instead of using genotypes, we used one randomly picked allele per individual and site for Bayesian clustering, PCA and nucleotide diversity analyses. Subsets of the genotype datasets outlined above thus included a 3,183,890 site allele dataset with one allele per individual and site as well as a SNP allele dataset containing 24,784 SNPs with minor allele frequency above 1%.

**Table 1:** Sampling site information.

Site	Habitat	Name	Catchment	Coordinates N / E	Year	N
L1	lake inlet	Altenrhein	Seegraben	47°29′08″ / 9°32′37″	2009/12	20
S1a	Stream	Seegraben airport	Seegraben	47°29′02″ / 9°33′29″	2009/12	10
S1b	Stream	Seegraben Pfaffenbrüggli	Seegraben	47°28′55″ / 9°33′50″	2012	10
S1	Stream	Seegraben Buriet	Seegraben	47°28′43″ / 9°33′30″	2012	21
L2	lake inlet	Marina Rheinhof	Rheintaler Binnenkanal	47°29′55″ / 9°33′25″	2013	10
S2	Stream	Oberriet	Rheintaler Binnenkanal	47°19′38″ / 9°34′24″	2007/09	20

The first and second axes of a principal component analysis (PCA, Fig 2A) separate the migratory lake and stream resident populations. The parapatric stream site S2 separates from the geographically nearest lake site along PC1 (ANOVA, F_1,89_ = 581.5, p < 0.001), whereas PC2 separates individuals of the other, shorter stream from the sympatrically breeding migratory lake fish (Fig 2A). In particular fish from the most upstream site S1 in this shorter stream were most distinct on PC2 (ANOVA, F_1,89_ = 106.9, p < 0.001) from the fish caught further downstream and those caught in the lake inlet (Fig 2A). These patterns translated into significant mean pairwise F_ST_ between the most upstream site in the sympatric stream S1 and the downstream stream sites as well as the lake inlet site L1, and also between the parapatric stream site S2 and its corresponding lake site L2 (Fig 2B). Stickleback from both upstream stream sites were also significantly differentiated from each other, while there was no significant differentiation either between the two lake sites or between these lake sites and the downstream sites in the sympatric stream (S1a and S1b, Fig 2B), suggesting that the migratory lake stickleback form a single population. The genetic resemblance of most S1a and S1b individuals to lake stickleback (Fig 2A) is in line with field observations: individuals collected at S1a and S1b were phenotypically mostly lake ecotypes caught during their upstream breeding migration, whereas resident stream ecotypes were relatively rare at these sites and were most common at site S1. Assignment of individuals by a Bayesian clustering algorithm implemented in STRUCTURE supported this presence of predominantly lake ecotypes but also revealed some stream ecotypes at sites S1b and S1a (S2 and S3 Figs). This analysis also showed that some intermediate individuals occur at L1, S1a, S1b and S1, indicative of ongoing gene flow.

### Distribution of genetic differentiation across the genome

**Fig 3:**
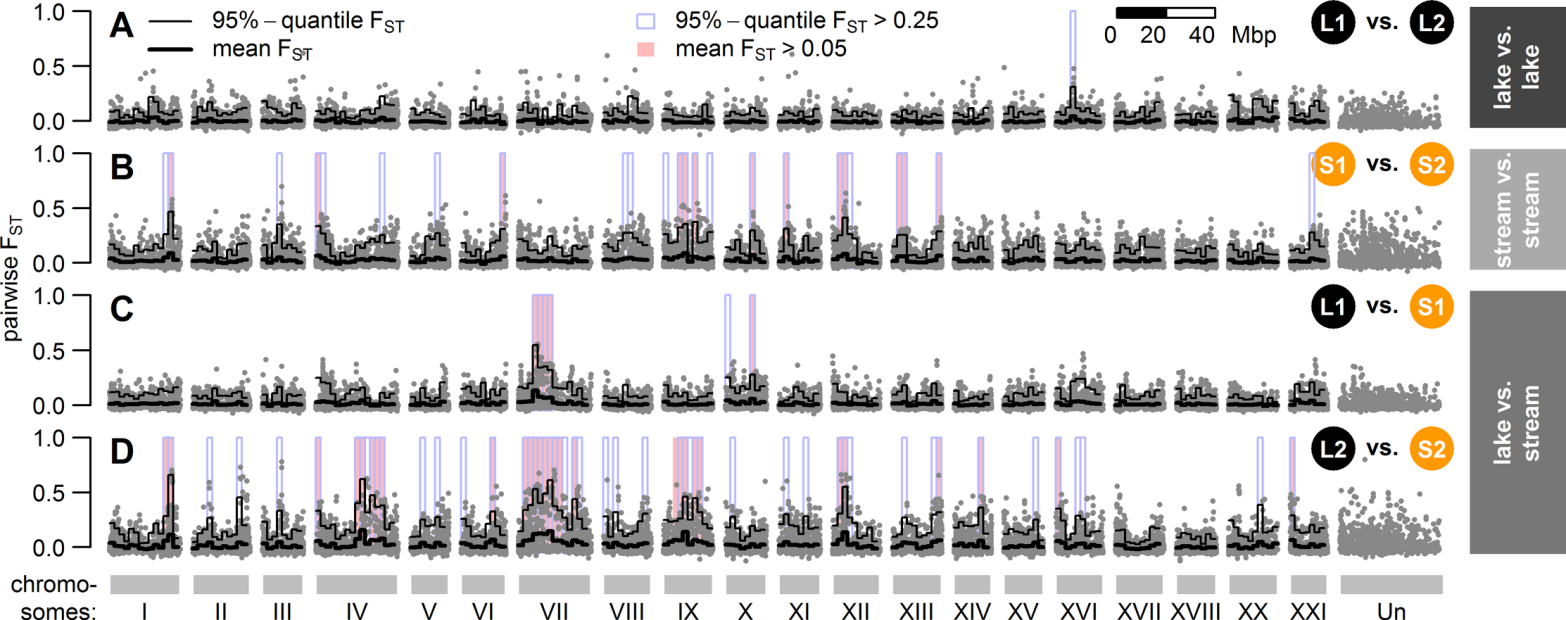
Distribution of pairwise differentiation (F_ST_) across the genome. Panels A and B show comparisons between sites with similar habitat **(A)** lake-lake (parapatric), **(B)** stream-stream (allopatric). Panels **(C)** and **(D)** show the two replicate lake-stream comparisons L1/S1 (sympatric breeding) and L2/S2 (parapatric breeding; see S4 Fig for other pairwise comparisons). Grey dots show single SNP pairwise F_ST_ estimates and black lines show F_ST_ means (bold) and 95%-quantiles (thin) in 2 Mb wide, non-overlapping windows across the genome. Windows with elevated differentiation are highlighted with blue background frames (mean F_ST_ > 0.05) and red background bars (95%-quantile F_ST_ > 0.25).

In the stream where breeding is sympatric (L1 vs. S1), we found a large region on chromosome VII and three smaller regions on chromosomes X and XI that show elevated differentiation between lake and stream ecotypes, while there was very little differentiation across the rest of the genome (mean pairwise F_ST_ in 2 Mb windows close to zero, Fig 3C). In contrast, our comparison of parapatric ecotypes (L2 vs. S2) revealed more genomic regions with elevated pairwise F_ST_ (Fig 3D), including the large region of elevated differentiation on chromosome VII that appeared in the sympatric lake-stream pair too, but was neither present in lake-lake nor stream-stream comparisons (Fig 3A and 3B). We measured heterogeneity in genome-wide differentiation by computing the coefficient of variation (CV) for pairwise F_ST_ in non-overlapping 2 Mb windows across the genome (see Materials and Methods). As expected, we found lower heterogeneity in genome-wide differentiation between lake and stream stickleback where breeding is sympatric (median CV_S1vsL1_ = 3.38) than where they breed in distant parapatry (median CV_S2vsL2_ = 4.03). A heterogeneous pattern of genome-wide differentiation was also found when the two most upstream stream sites were compared against each other (Fig 3B), whereas almost no genome-wide differentiation was seen between the two lake sites (Fig 3A).

**Fig 4:**
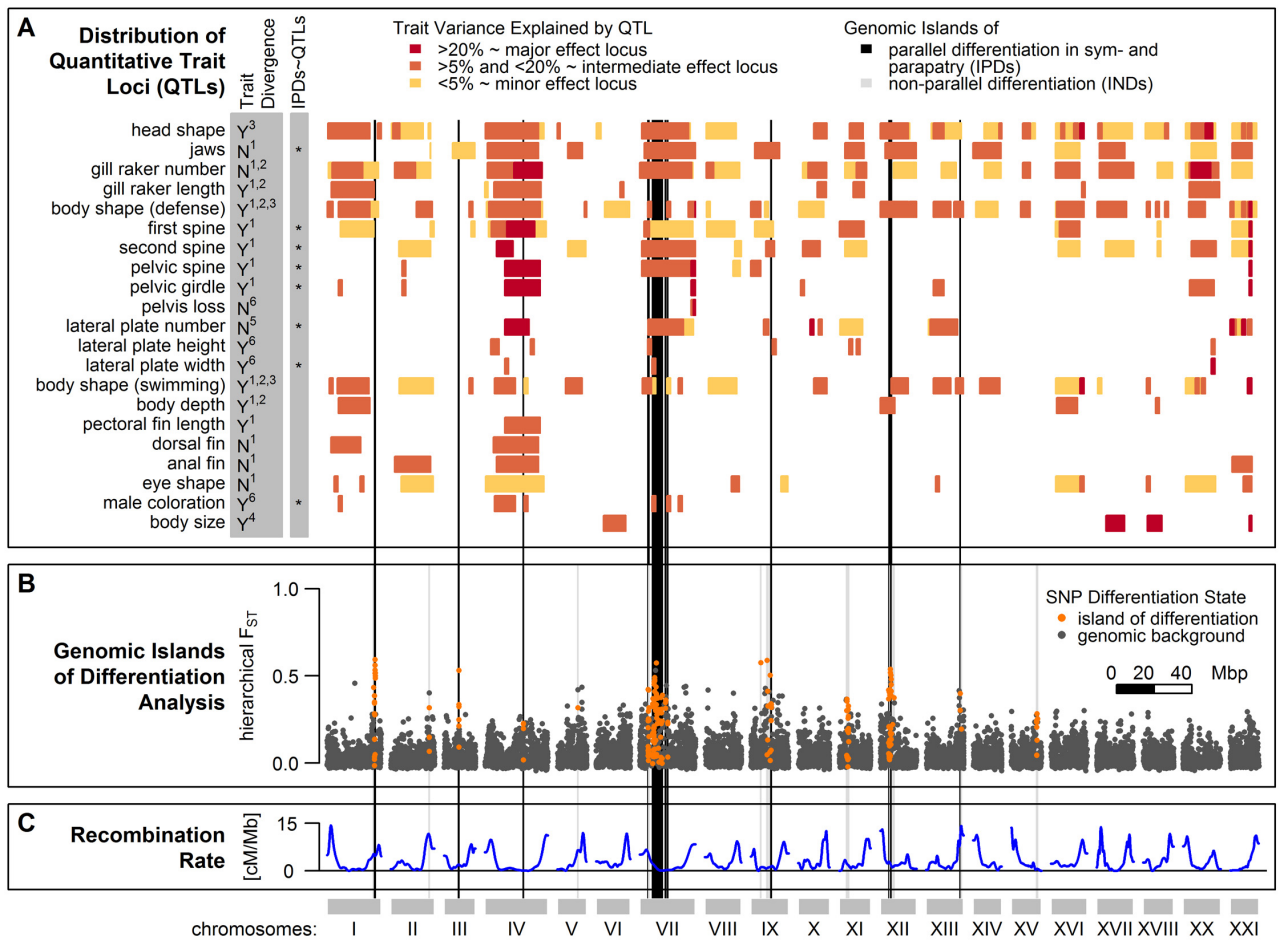
Genomic islands of differentiation among Lake Constance stickleback and distribution of Quantitative Trait Loci (QTL). **(B)** Of 37 genomic islands of differentiation identified in Lake Constance stickleback, 19 showed parallel differentiation between lake and stream ecotypes both in sympatry and parapatry (IPDs, black vertical bars), while non-parallel differentiation in 18 further islands (INDs, grey vertical bars) were mainly driven by differentiation between the parapatric ecotype comparison only. Dots show SNPs assigned to genomic islands of differentiation (orange) or the neutral genomic background (dark grey). **(A)** QTLs for traits previously studied among Lake Constance ecotypes and their overlap with parallel islands are shown. The left grey column indicates if traits have previously been found to be divergent among Lake Constance ecotypes (‘Y’ = yes) or not (‘N’ = no). Significant clustering of parallel islands inside QTLs for trait groups are indicated by asterisks in the right grey column. Blocks indicate 95% QTL confidence intervals (extent along x-axis) and effect sizes (color). References for phenotypic data: ^1^[59], ^2^[57], ^3^[65], ^4^[56] and S7B Fig, ^5^[46], ^6^S7A Fig. **(C)** Recombination rates across the stickleback genome estimated by Roesti et al. [77].

We defined ‘genomic islands of differentiation’ as genomic regions with an accumulation of unusually strongly differentiated SNPs (outlier loci; [76]) showing high differentiation measured over all populations grouped hierarchically (‘hierarchical F_ST_’, see Materials and Methods). We identified 1,251 SNPs (3.6%) as outliers in our dataset at the 5% alpha level and 242 SNPs (0.7%) at the 1% alpha level, close to what would be expected by chance. Importantly, however, these outliers were not randomly distributed across the genome and instead more clustered than expected even after accounting for variation in recombination rate (Ripley’s K function using genetic distances, S5 Fig). To infer the location and extent of ‘genomic islands of differentiation’, we followed a Hidden Markov Model (HMM) approach that assigns each SNP to one of three differentiation states, ‘genomic background’, regions of ‘exceptionally low’ and ‘exceptionally high’ differentiation ([76], see Materials and Methods). We identified 37 genomic regions of ‘exceptionally high’ differentiation considered here as ‘genomic islands of differentiation’ (Fig 4B). No regions of ‘exceptionally low’ differentiation remained significant after correcting for multiple tests (see Materials and Methods). These 37 genomic islands of differentiation were spread across 11 of the 20 autosomes, with a concentration on chromosome VII (Fig 4B). Each island consisted of 1 to 26 SNPs, spanning up to 990 kb in size (S1 Table). The presence of islands of differentiation was overall negatively associated with recombination rates (Fig 4C, S2 Table). This association was mostly driven by the accumulation of islands on chromosome VII, clustering in a genomic region showing low to intermediate levels of recombination (S6 Fig) and further islands falling into such regions on chromosomes IV, IX and XV (S2 Table, Fig 4). However, if the same test was repeated for each chromosome, the strength of this association varied and was even positive on chromosome II (S2 Table), where a genomic island falls into a high recombination region (Fig 4C). Moreover, some of the strongest localized reductions of recombination in the stickleback genome such as on chromosome I [77] are not differentiated among the studied populations (Fig 4C). Thus, genomic islands of differentiation identified in our study are not exclusively bound to low recombination regions.

**Fig 5:**
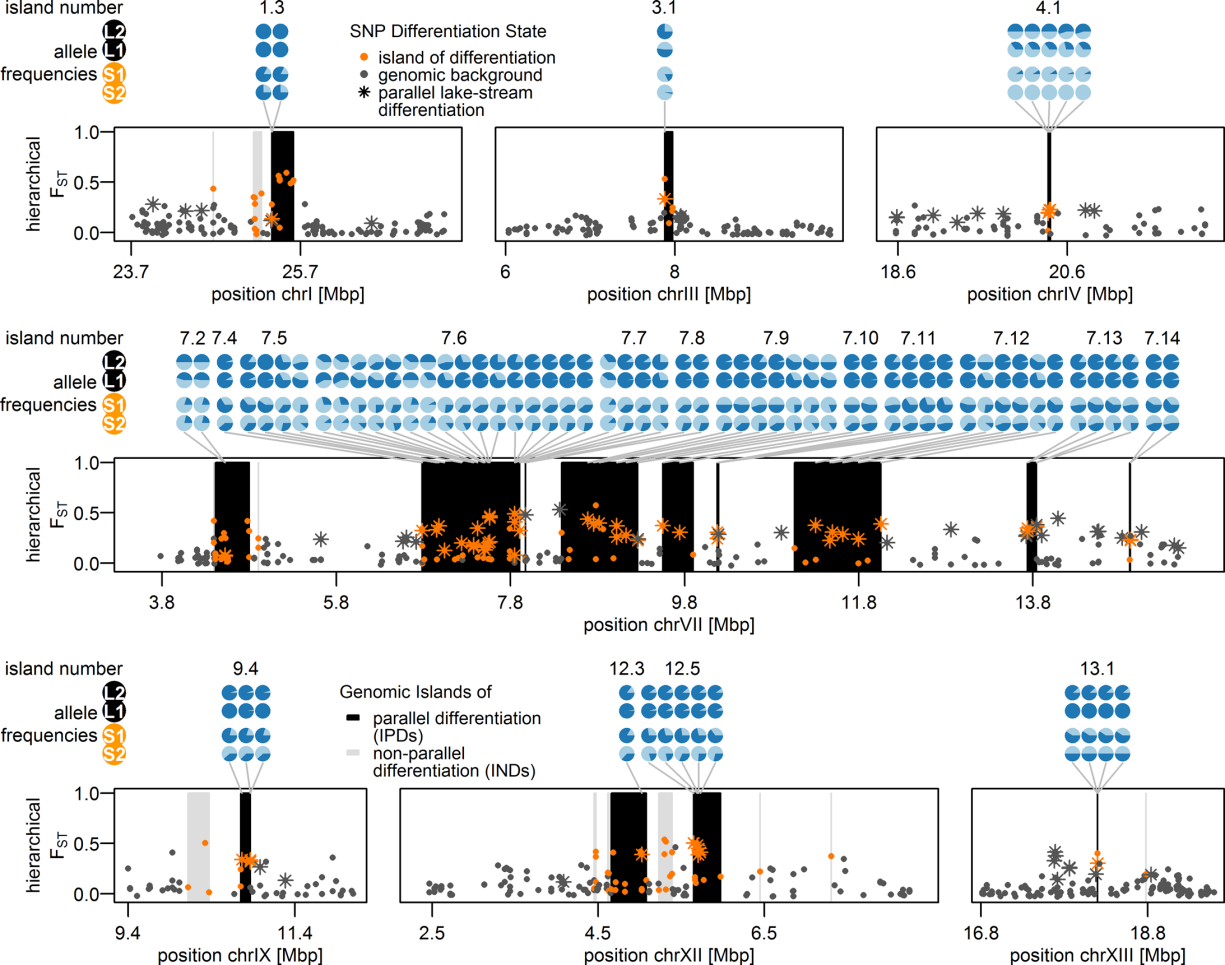
Allele frequencies of parallel lake-stream differentiation SNPs in 19 islands of parallel lake-stream differentiation. Pie charts represent allele frequencies at the sites S1, S2, L1 and L2 of parallel divergent SNPs within parallel islands. Light and dark blue segments show the respective proportions of stream-like and lake-like alleles at those sites. Star-like dots show SNPs indicative of parallel lake-stream differentiation, while color coding of dots and vertical bars are as in Fig 4.

We observed parallel allele frequency changes between the lake ecotype population and both resident stream ecotype populations from the two streams in 19 of the 37 genomic islands of differentiation (Figs 4B and 5). Importantly, very few of these 19 islands were differentiated between the two stream ecotype populations or among lake ecotypes sampled at sites L1 and L2 (Fig 3). These ‘islands of parallel differentiation’ are thus prime candidates for harboring genes involved in ecological speciation. Interestingly, 12 of the 19 parallel islands clustered in a 10.5 Mb stretch on chromosome VII with low to intermediate recombination (S6 Fig), and the highest levels of pairwise differentiation were observed in this region (Fig 3C). Furthermore, one other parallel island found on chromosome I is located in a region that has previously been described as an inversion segregating between marine and freshwater stickleback [25]. The remaining six parallel islands were each found on different chromosomes (III, IV, IX, XII and XIII, Figs 4B and 5, S1 Table). All but one parallel islands contained multiple SNPs differentiated among ecotypes breeding in sympatry (S1 Table).

**Fig 6:**
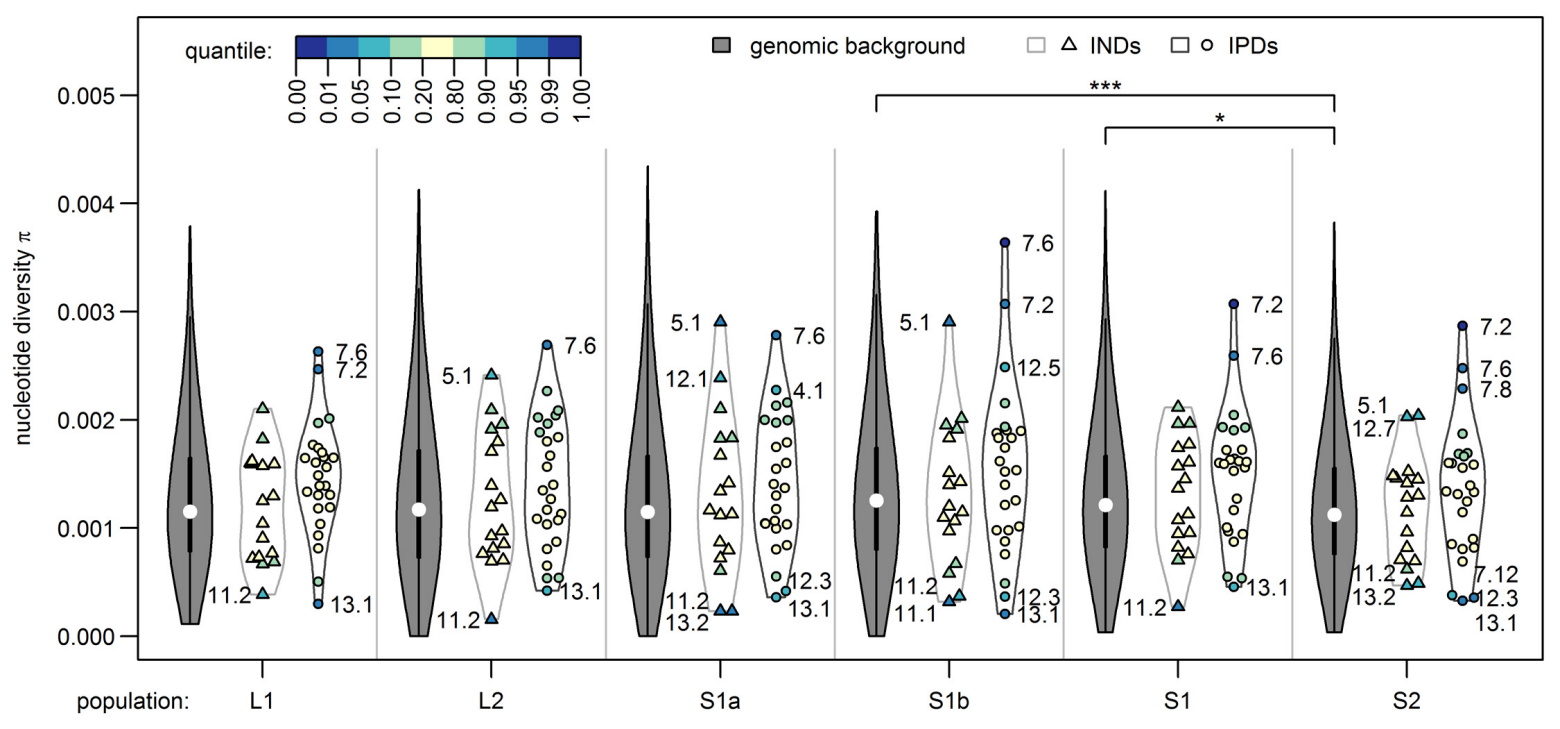
Nucleotide diversity inside and outside genomic islands for each population. Genomic islands of parallel differentiation (IPDs) show on average a slightly but not significantly higher diversity than both the genomic background and genomic islands of non-parallel differentiation (INDs) in all populations and diversity did not differ between populations. Nucleotide diversity was calculated in non-overlapping windows spanning multiple RAD-loci that together contained 2,500–2,685 sequenced bases. Windows were grouped into genomic background (n = 1,104 windows, filled violin plots), overlapping with non-parallel islands (n = 17, triangles) and with parallel islands (n = 25, circles) and tested for group differences in mean nucleotide diversity using t-tests (n.s.: not significant; *: Bonferroni-adjusted p-value < 0.05). Marker color indicates how extreme genomic island diversity is compared to the genomic background.

These 19 parallel islands appear to be rather robust to gene flow given the significant allele frequency differentials observed among the sympatric ecotypes. On the other hand, islands of non-parallel differentiation seem mainly driven by large frequency differentials only in the parapatric ecotype comparison (L2 vs. S2), which were not differentiated between ecotypes breeding in sympatry (S1 Table). Overall, parallel islands that are robust to gene flow were associated with regions of low to intermediate recombination rate, also including a single case within a known inversion polymorphism region [26], while the association between presence of islands with non-parallel differentiation and recombination was much weaker and the sign of this association varied across chromosomes (S2 Table). Islands with non-parallel differentiation showed on average slightly but not significantly lower diversity than both the genomic background and that found in parallel islands (Fig 6), which is compatible with the action of background selection, with a past selective sweep pre-dating the population splits or with multiple local selective sweeps leading to non-parallel differentiation between sampling sites. Parallel islands showed on average slightly higher levels of nucleotide diversity than the genomic background and diversity levels did not differ between sampling sites (Fig 6). The observed increase in diversity is compatible with selection on standing genetic variation and notably the highest diversity among parallel islands is found in chromosome VII islands 7.6 and 7.2, consistently across all sampling sites (Fig 6). The only parallel islands with reduced diversity show the same reduction in all populations (islands 13.1, 12.3 and 7.12, Fig 6), possibly due to background selection, a past sweep or multiple sweeps in each population with the same alleles favored in the respective habitat. We thus have little evidence for hard selective sweeps in parallel islands, although incomplete sweeps may not have led to a reduction in diversity yet. Rather, our data suggests that selection on standing genetic variation was acting in both stream and lake environments, or that sweeps have not been completed in either environment, as we observe similar levels of elevated diversity in both habitats.

**Fig 7:**
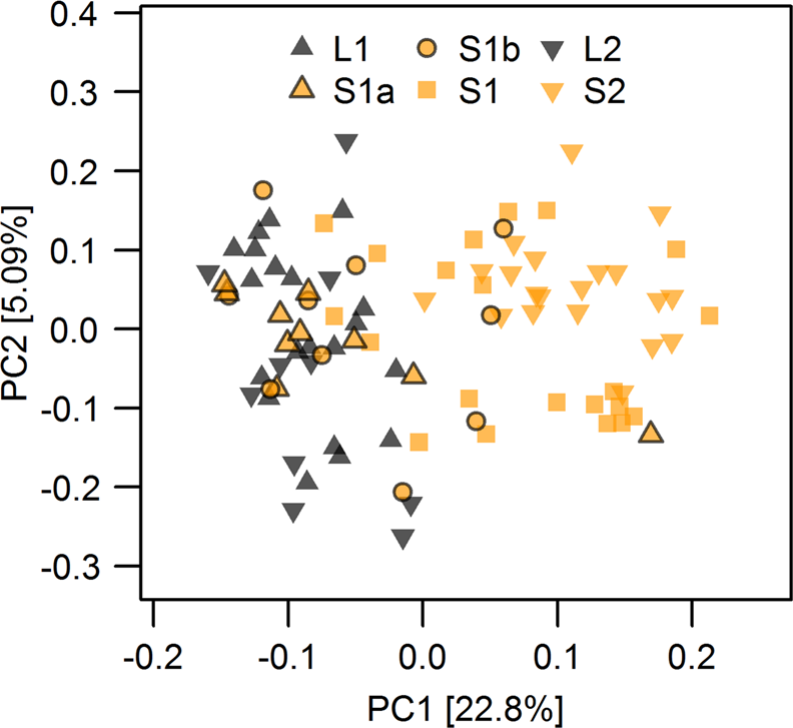
Principal component analysis of parallel lake-stream differentiation SNPs. PC1 separates migratory lake and resident stream ecotypes based on the SNPs found in parallel genomic islands of lake-stream differentiation shown in Figs 4B and 5(n = 75). Individuals with both lake-like and stream-like genomic signatures occur at stream sites S1a, S1b and S1, but lake ecotypes dominated at sites S1a and S1b, while stream ecotypes were most common at S1. Only stream ecotypes occurred at site S2 and only lake ecotypes at sites L1 and L2. Fill colors indicate the habitat in which individuals were caught, four stream habitat sites (orange) and two lake shore sites (black).

We classified the two alleles of SNPs showing parallel allele frequency changes between the lake ecotype and both populations of stream ecotypes either as lake-like or stream-like according to their major frequency (Figs 5 and S8). A PCA based on these SNPs only (Fig 7) recovered the distribution of ecotypes over sampling sites: most individuals from sympatric stream sites S1a and S1b showed a lake-like genomic signature, but one and three of ten individuals at sites S1a and S1b respectively did show a stream-like genomic signature (Fig 7). As expected, a stream-like genomic signature was shown by a majority of the fish at site S1, with only four of twenty individuals displaying lake-like genotypes (Fig 7). None of the 20 fish at site S2 showed a lake-like genomic signature, and none of the 30 fish at lake sites L1 and L2 showed stream-like genomic signatures. We observed increased levels of linkage disequilibrium (LD) among almost all chromosome VII islands at site S1a and to a lesser extent at S1b and S1, while stickleback from the lake sites L1, L2 and the parapatric stream site S2 revealed two haplotype blocks on chromosome VII (S9 Fig). These patterns of LD are in line with the presence of both ecotypes in sympatry at sites S1a, S1b and S1. There was overall little LD between islands located on different chromosomes, except for islands 1.4, 4.1 showing some LD with islands on chromosome VII at sites S1a and S1b, and islands 9.4 and 13.1 showing elevated LD with each other and with chromosome VII islands at sites S1 and S1b (S9 Fig), again in agreement with the presence of both ecotypes in sympatry at sites S1a, S1b and S1, and gene flow between them. Similarly, lake populations L1 and L2 displayed elevated LD between islands 12.3, 12.5 and islands on chromosome VII.

### Trait associations with islands of parallel differentiation robust to gene flow

The 19 parallel islands robust to gene flow overlap with 207 quantitative trait loci (QTLs) that have been previously identified in other stickleback populations (Fig 4A, S3 Table, [78–103]). Ten of these QTLs are major effect QTLs located on chromosomes IV and VII, while the other QTLs are reported to have minor to moderate effect sizes (Figs 4A and S6 and S3 Tables). We grouped QTLs into 32 phenotypic traits and tested if the 19 parallel islands clustered inside any of these traits more than expected by chance. For this, we permuted the positions of the 19 parallel islands across the genome, both on the physical and on the genetic map to account for recombination rate variation biasing confidence intervals of QTLs (see Materials and Methods). This test identified a significant clustering of parallel islands inside QTLs for 11 of 32 traits (Figs 4A, S10 and S11). We checked if our lake and stream ecotypes were phenotypically divergent in these traits [56–59,65]. Six of these 11 traits with clustering of parallel islands concerned divergent traits: male breeding coloration and most defense morphology related traits such as first and second dorsal spine, pelvic spine, pelvic girdle morphology and lateral plate width (Fig 4A). Three of the remaining five traits with clustering of parallel islands inside their QTLs have not been studied yet among Lake Constance ecotypes (S11 Fig), while the last two traits, jaw morphology and lateral plate number, are not divergent among Lake Constance ecotypes studied here (Figs 4A and S7B). 21 traits did not show significant clustering of parallel islands inside their QTLs, while many of them are still overlapping with parallel islands, including traits divergent among Lake Constance ecotypes such as head shape, body size, lateral plate height, body depth and body shape [56–59,65]. However, two of these traits without clustering, body depth and body shape, have been shown to be controlled largely by phenotypic plasticity in response to the environment among these Lake Constance ecotypes [65].

### Candidate targets of selection

We searched the 19 parallel islands for genes that might be candidate targets of divergent selection between ecotypes. They contained 243 Ensembl predicted genes, including 208 genes with a known ortholog in human or zebrafish (S4 Table, [104,105]). No enrichment of gene ontology terms was found in this gene set. However, a few of these genes might be candidate targets for divergent selection between habitats or life histories because they are involved in the development of traits that are divergent among ecotypes. For instance, *beta-1*,*3-glucuronyltransferase 3* (*b3gat3*), positioned in island 7.6, is involved in cartilage and gill structure morphogenesis in zebrafish [106–109]; *phospholipase C beta 3* (*plcb3*), in island 7.6, is involved in cartilage and viscerocranium morphogenesis, influencing gill raker and pharyngeal jaw development [110–113]. Similarly, *integrin alpha 5* (*itga5*, island 12.5) is involved in pharyngeal arch, head and eye development [104,114,115] and *claudin 7a* (*cldn7a*, island 7.9, [116]) and *phosphatidylinositol 4-kinase type 2 beta* (*pi4k2b*, island 9.4, [117]) are involved in head development. In addition, *ring finger protein 41* (*rnf41*, island 12.5) is involved in melanocyte differentiation [118], thus potentially influencing pigmentation and camouflage. *Fras1 related extracellular matrix 1a* (*frem1a*, island 7.12) is involved in morphogenesis of pectoral, caudal, anal and dorsal fin as well as pharyngeal jaw [110,119], and *meiosis 1 associated protein (M1AP*, island 7.7) is involved in spermatogenesis, thus possibly a target of sexual selection [104]. *H6 family homeobox 4* (*hmx4*, island 7.2) is involved in retinal cone development and retinoic acid biosynthesis and might thus be relevant to vision and thus possibly to adaptation to deeper water habitats in the lake versus shallow stream habitats and also mate choice [120,121]. While we lack full sequences of any gene in the stickleback genome, our RAD-sequencing data contained two non-synonymous SNPs in the genes *plcb3* and *M1AP*, that both show high and parallel lake-stream differentiation. A pairwise F_ST_ = 0.50 in the sympatric (L1 vs. S1) and F_ST_ = 0.43 in the parapatric comparison was estimated for the non-synonymous SNP within *plcb3* and F_ST_ = 0.35 and F_ST_ = 0.57 for sympatric and parapatric comparisons respectively for the non-synonymous SNP in *M1AP*.

## Discussion

### Genomic signatures of ecotype formation in the presence of gene flow

We characterized genomic differentiation among very young lake and stream stickleback ecotypes, breeding in sympatry and in distant parapatry in two different streams, to understand processes acting at what might be the onset of ecological speciation. Our first and perhaps most salient result is that ecotypes are genetically differentiated at multiple places in the genome, both in sympatry and in parapatry. Hence we can rule out that these very young ecotypes are maintained by adaptive phenotypic plasticity only. Instead, significant genomic differentiation has arisen within less than 150 generations of evolution since the arrival of stickleback in Lake Constance. Because differentiation is found not just in parapatry but also in sympatry, our results are consistent with the incipient stage of ecological speciation. In the following we will discuss the evidence and attempt inferences of evolutionary mechanisms from our genomic data.

Different from previous lake-stream stickleback studies, we investigated pairs of resident stream and potamodromous lake ecotype, the latter breeding in streams but spending most of its adult life in the lake. Combined with the migratory behavior of the latter, our sampling of both ecotypes from a short and a long stream gradient allowed us to compare phenotypically and ecologically very similar pairs of ecotypes where breeding is sympatric in one but parapatric in the other pair (Fig 1). Of the 37 genomic islands of differentiation identified in this system, 19 islands distributed across eleven chromosomes showed differentiation between the ecotypes breeding in sympatry. These islands thus persist in the face of gene flow (S1 Table). In contrast, where ecotypes breed in distant parapatry, all 37 genomic islands (Fig 3, S1 Table) show differentiation among the ecotypes, including the 19 islands also differentiated among the sympatrically breeding ecotypes. Both the heterogeneity of genome-wide differentiation and the average level of differentiation are higher in the parapatric comparison where there is much less opportunity for gene flow, in keeping with models of ecological speciation with gene flow [3–6]. Remarkably, all genomic islands with differentiation in sympatry thus showed differentiation in parapatry too, with the same alleles favored in the same ecotype (Fig 5). Some of these parallel islands, islands 1.3, 7.9, 7.10 and 7.13 (S3 Tab.), overlap with SNPs identified as divergent between the lake ecotype and stream ecotype populations North, West and South-West of Lake Constance [53]. While genetic drift, background selection, or local adaptation could all have created islands in a single contrast, islands that are repeatedly divergent between the lake ecotype and two stream ecotype populations, with the same alleles favored in the same habitat and with divergence persisting in the face of gene flow, suggest that habitat- and/or life-history-associated divergent selection have led to their emergence.

A striking feature of these islands of parallel differentiation that are found both in sympatry and in parapatry in Lake Constance stickleback is that they overlap with many QTLs and cluster in some QTLs for traits that are clearly differentiated between these ecotypes (Fig 4A) [56–59,65]. Although most QTLs have been identified in different populations, possibly with other causative mutations, the same genes might be involved in controlling the traits that differ among Lake Constance stickleback ecotypes. Many ecologically relevant traits controlling e.g. defense morphology and head shape are among these overlapping traits, as well as two traits, body size and male coloration, that are relevant to mate choice and thus possibly to pre-zygotic reproductive isolation. Body size often differs between migratory and resident stream fish life history morphs, not just in stickleback [122]. Lake Constance migratory lake fish are much larger than the stream residents [56,58] and body size is known to often mediate assortative mating in stickleback [123]. In addition, we identified a number of candidate genes within the islands of parallel differentiation that may underlie phenotypes under natural and sexual selection that diverge between the Lake Constance ecotypes. Phenotypic plasticity in some traits [65] might be responsible for additional differences between ecotypes and may also have reduced the power to detect associations between some of the phenotypic differences and genomic differences. The associations between islands of parallel differentiation and QTLs for divergent traits we observed support the view that divergent selection between migratory and resident life histories and lake and stream habitats underlies the genomic divergence persisting in sympatry.

That the genomic basis of various ecologically relevant traits is often highly clustered on a few chromosomes in stickleback [96] may have facilitated the simultaneous divergent evolution of multiple phenotypic traits: several feeding and defense morphology trait QTLs as well as male coloration QTLs are clustered on chromosomes IV and VII (Fig 4A). Divergent selection on any gene in these regions could then possibly have led to phenotypic divergence in several other traits, given sufficient standing genetic variation and linkage disequilibrium in that genomic region. Furthermore, given that both adaptation and reproductive isolation traits are located in these regions, divergent selection in these genomic regions may serve as a nucleus for ecological speciation. Most of the genomic islands of parallel differentiation are found in a region of low to intermediate recombination on chromosome VII, which shows the highest level of pairwise differentiation in sympatry (Fig 3C) and in parapatry in our populations (Fig 3D) and also among the lake population and two stream populations North and West of Lake Constance [53]. Furthermore, the parallel island 1.3 (S3 Table), also found divergent between three stream populations North, West and South-West of Lake Constance and the lake ecotype [53], overlaps with a region known to be polymorphic for an inversion that differentiates marine and freshwater stickleback [26], suggesting that this inversion could potentially be polymorphic and suppressing recombination in this pair too [53]. These observations are consistent with models and evidence that the recombination landscape may influence adaptation and ecological speciation the face of gene flow [6,15,26]. Nevertheless, genomic islands of differentiation in our sympatric stickleback ecotypes are not exclusive to regions of low recombination (S2 Table), suggesting that recombination rate variation alone cannot explain the overall differentiation patterns we observe. Rather, the interaction of life history-driven and/or habitat-driven divergent selection with recombination rate variation and gene flow seem to determine patterns of genomic differentiation. Furthermore, that several unlinked genomic regions beyond chromosome VII diverge in parallel suggests that either many genomic targets are under correlated divergent selection, that partial reproductive isolation has evolved or that a combination of both is maintaining the genomic differences between these ecotypes in sympatry, a situation that is thought to characterize the beginnings of ecological speciation [2]. This observation is consistent with the hypothesis that genomic islands with large and pleiotropic effects may act as seeds for ecological speciation with gene flow, when selection favors linkage disequilibrium between such a region and genes elsewhere in the genome [1].

Heterogeneous genomic divergence with islands of differentiation is also expected under scenarios of divergence without gene flow [14,40], but this could only occur if complete reproductive isolation had already evolved among now sympatrically breeding lake and stream stickleback. This seems rather unlikely: first, there is no evidence that any pair of stickleback ecotypes studied before has reached complete reproductive isolation after less than many thousand generations of divergence. Second, our results suggest ongoing gene flow as we observe that the geographical opportunity for gene flow is negatively related to the number of islands that show differentiation in the ecotype pairs (S1 Table) and to the magnitude of pairwise differentiation within islands (Fig 3). Furthermore, genetically intermediate individuals between lake and stream ecotypes occur where they breed in sympatry, as suggested by genome-wide variation (Figs 2A and S2) and by patterns of variation and LD in genomic islands of parallel differentiation (S8 and S9 Figs).

### Comparisons with older stickleback ecotypes and species

Although genomic changes associated with habitat-dependent adaptation in stickleback have been extensively studied [24–26,36,53,70,88,124–132], genomic differentiation that persists among sympatrically breeding stickleback species has only been demonstrated in a few small lakes at the Pacific Coast of British Columbia [25], perhaps the most classical cases of ecological speciation [133–135]. This repeated evolution of sympatric limnetic and benthic stickleback species has occurred over the past ~12,000 years and is thought to have included an allopatric phase, after which these lakes were colonized a second time from the ocean [25,72]. Despite the very different evolutionary histories and divergence times of the Canadian limnetic-benthic stickleback species pairs and the ecotype pairs from Lake Constance, the number of chromosomes containing genomic islands with parallel differentiation is remarkably similar between the two systems (Constance eight, versus Canadian limnetic-benthic ten chromosomes) and the number of such islands is even higher among Lake Constance ecotypes (19 versus 15 islands, but note that different methodologies to define islands were used in [25]). The number of divergent regions among sympatric Lake Constance ecotypes is also higher than that found among parapatric lake and stream ecotypes from several catchments on the Haida Gwaii archipelago, Canada [70]. The latter lake and stream ecotypes also evolved from a marine ancestor over the past ~12,000 years since the retreat of the ice sheets, or potentially even earlier and survived in ice age freshwater refugia [136–138]. The similarity in number of diverging chromosomes among these systems is surprising, as older, more diverged and more strongly reproductively isolated ecotypes are expected to accumulate divergence across much of the genome with time, due to background selection, selection unrelated to speciation itself (including divergent selection between species) and due to drift. However, the stream and lake ecotypes that we studied emerged in only 150 years [46], suggesting that genomic regions differing between older ecotypes or species might already have been involved at the onset of ecological speciation. Given the short time that was available for evolutionary divergence and the observed patterns of diversity in parallel islands, the adaptive variation differentiating Lake Constance ecotypes must have originated from older, standing genetic variation present in the colonizing linage from the Southern Baltic Sea catchments.

Despite high numbers of repeatedly diverging genomic regions among Lake Constance ecotypes, there is limited overlap in identity with such regions identified in lake-stream stickleback ecotype pairs from Canada, Alaska, Northern Germany and elsewhere [36,70,130], or with divergent regions identified among freshwater-marine ecotypes [24,26,45,139] or limnetic-benthic species [25,93]. Of the 19 genomic islands of parallel differentiation we identified in our study, only seven regions have been previously found as outlier regions between ecotypes or species outside the Lake Constance system (S3 Table). Most strikingly, island 1.3 has been identified as divergent between allopatric marine and freshwater stickleback populations [24–26,45,125,127] and between lake and stream ecotypes in Northern Germany (S3 Table, [36]), and has been shown to be an inversion for which alternative haplotypes are favored in one or the other environment [26,53]. Three other shared outlier regions on chromosome VII have previously been identified as outliers: Island 7.14 on chromosome VII is divergent between fully sympatric limnetic and benthic stickleback in one of three studied lakes in British Columbia, Canada [25], in eight out of nine parapatric lake-stream pairs on Haida Gwaii and Vancouver Island, Canada [70,130], as well as in an allopatric marine-freshwater comparison from Northern Scandinavia [127]. Island 7.11 on chromosome VII, is differentiated between multiple allopatric marine and freshwater populations from across the Northern hemisphere [26] and island 7.7 between parapatric lake and stream ecotype from Alaska [36]. Finally, islands 3.1, 12.3 and 12.5 are divergent between multiple marine and freshwater populations [24,125,127] and islands 12.3 and 12.5 both between lake and stream ecotypes from Alaska [36] and among Norwegian freshwater populations [125] (S3 Table).

There is a discrepancy between the widespread genomic parallelism among marine-freshwater ecotypes that have been studied around the Northern Hemisphere [24,26,45] and limited shared divergence among lake-stream ecotypes from different lakes [25,32,36,70,130]. One reason for this discrepancy could be the more diverse and complex evolutionary histories of stickleback populations living and diverging within freshwater bodies. Marine stickleback have larger effective population sizes resulting in large standing genetic variation, much of which is broadly shared among marine stickleback populations [43]. In contrast, standing genetic variation is smaller and less widely shared among isolated and geographically disjunct freshwater populations. The combination of these factors may explain the lack of parallelism in phenotypic [66,130,140–142] and genomic divergence [32,36,70,130], as well as the large phenotypic diversity [46,57,59,143–145] observed among lake ecotype stickleback and among stream ecotype stickleback from different systems.

### Models for rapid genomic lake and stream ecotype divergence

Contrary to the reported lack of phenotypic and genomic parallel evolution between lake-stream stickleback ecotype pairs from other regions of the world [25,32,36,70,130], we find patterns of parallel differentiation at the genomic level between a lake ecotype and stream ecotype populations in two streams of the recently colonized Lake Constance system. Multiple scenarios of colonization and ecotype formation could plausibly explain the observed parallel genomic differentiation. First, if lake-adapted stickleback were originally introduced, multiple streams may have been colonized independently and repeated recruitment of adaptive alleles could have occurred from the same initial standing genetic variation, resulting in parallel genomic differentiation. This ‘lake first’-scenario would be a true ‘parallel evolution’ scenario [146] and the marine-like phenotypic composition of Southern Baltic Sea catchment stickleback that colonized the Lake Constance system may be in favor of this scenario. Second, if stream-adapted stickleback were introduced into the system, ecotypic differentiation may have evolved once at the habitat transition to the lake. Under this ‘stream first’-scenario, colonization of other streams may have occurred after the evolution of a lake ecotype, either (a) through long-distance migration of stream genotypes through the lake to other streams or (b) through repeated adaptation from standing genetic variation retained in the lake ecotype. The former would require fortuitous, simultaneous long-distance dispersal of several stream-adapted stickleback to a new stream, possibly aided by active habitat selection [147], and would not be considered a case of parallel evolution. The latter would need allele combinations or haplotypes favored in the original stream ecotype to be added to the standing genetic variation of the lake ecotype via gene flow and then be recruited from the standing variation into newly evolving populations of stream ecotype in other streams. This mechanism, also referred to as ‘transporter hypothesis’, would be considered parallel evolution [146] and was proposed to explain the widespread genomic and phenotypic parallelism among marine and freshwater stickleback [148]. Long-distance dispersal and transporter mechanisms are not exclusive and a combination of dispersal between streams and transport of adaptive variants via standing variation in the lake population are possible. Third, a generalist could have been introduced into the system and rapidly expanded its range to both stream and lake environments, followed by adaptation to these habitats. Adaptation may have involved standing genetic variation spreading with the initial expansion or ‘transported’ to replicate stream habitats later, both ideas compatible with parallel evolution. A fourth scenario could be secondary contact between already divergent lake and stream ecotypes that independently colonized the lake and effluent streams, leading to parallel patterns of differentiation between lake and stream populations but no in-situ parallel evolution.

We think that the ‘generalist’ scenario is the most likely scenario, given the patterns of genomic variation observed in the populations studied here: genomic diversity levels in parallel genomic islands suggest that selection on standing variation occurred in both lake and stream ecotypes (Fig 6). ‘Lake first’ and ‘stream first’ scenarios may however lead to very similar genomic patterns of variation due to selection on standing genetic variation and thus are plausible alternatives we cannot reject. In contrast, we exclude a secondary contact scenario between already differentiated lake and stream stickleback. Such a model cannot explain that our two stream ecotype populations are genetically as distinct from each other as either is from the lake ecotype (Figs 2B and S2). Furthermore, phylogeographic reconstructions and population genetic analysis also clearly reveal our lake and stream ecotypes as closely related sister groups to the exclusion of other Swiss and central European populations [46,59] and suggest they have received only very little if any gene flow from outside the system [55]. This does not rule out that some of the standing variation on which selection acted could have arrived in the gene pool through contributions from outside, a hypothesis we are currently investigating. In contrast, we think that a secondary contact scenario likely applies to stream and lake populations from the North, West and South-West of Lake Constance: Mitochondrial haplotypes from Rhine (South-West) and Rhone (North) lineages are numerous in the streams of those regions, whereas the adjacent lake populations are nearly exclusively composed of Baltic Sea catchment haplotypes [56]. Similarly, fish with reduced body armor occur at high proportions in these more Western streams but not in the adjacent lake [53,56], whereas this contrast is completely lacking in the South-Eastern sections of the lake that we studied here and reduced armor is rare in Southern Baltic Sea catchment stickleback with the same haplotype as Lake Constance stickleback [46,55,61].

By studying sympatric ecotypes with ongoing gene flow, we show that adaptive genomic differentiation, reminiscent of incipient speciation, has arisen in a very short period of time (150 years or ~100 generations). Genomic and phenotypic divergence between a migratory lake ecotype and two populations of resident stream ecotypes possibly involved the re-use of standing genetic variation and resulted in the persistence of stream ecotype populations even where there is ample opportunity for gene flow between ecotypes in sympatry. We propose that the high levels of differentiation observed between ecotypes despite existing gene flow was facilitated by genomic properties such as reduced recombination and the genomic co-localization of genes controlling several phenotypic traits relevant to adaptation and mate choice.

## Materials and Methods

### Study site and collection

We sampled adult stickleback in spring 2007/09 and 2012/13 from six sites in two streams draining into Lake Constance and the lake shores close to the stream inlets (Fig 1A, Table 1). From each site, 10–21 individuals from the same year (except for site S2, for which fish from 2007 and 2009 were combined) with both sexes equally represented were randomly picked for genomic analyses.

### Ethics statement

Stickleback were caught using minnow traps and hand nets and subsequently anesthetized and euthanized in clove oil solution, in accordance with granted permits issued by the fishery authorities of the canton St. Gallen. Fish collection followed the Swiss veterinary legislation in concordance with the federal food safety and veterinary office (FSVO) and the cantonal veterinary office in St. Gallen (Veterinäramt Kanton St. Gallen).

### Morphological analysis

In addition to morphological, ecological and life history traits described earlier from the Lake Constance system [46,56,57,59,65], we quantified a previously unexplored morphological trait, lateral plate cover, that we observed to diverge among lake and stream ecotypes. We measured the height of the first 28 lateral plates after the pelvic girdle in all fully-plated stickleback following [94] and body depth at the first dorsal spine (‘BD1’, following [59]) from sites L1, L2, S1 and S2 using ImageJ v1.49 [149]. We performed a PCA on size-corrected plate heights, i.e. residuals from linear regressions of plate height against body depth at the first dorsal spine, and used an ANOVA to test for differences in PC1 between lake stickleback from L1 / L2 and stream stickleback from S1 / S2 (S7A Fig). Furthermore, we identified the plate morph of each fish by counting lateral plates following [150] and tested for differences between lake stickleback from L1 / L2 and stream stickleback from S1 / S2 (S7B Fig).

### RAD sequencing

We prepared three RAD libraries following Baird et al. [151] with slight modifications: We used 400 ng genomic DNA per sample and digested each for 12 hours with four units *SbfI*-HF (New England Biolabs). We multiplexed 98, 77 resp. 49 individuals per library, after the ligation step using P1 adapters (sensu [151]; synthesized by Microsynth) with custom six base pair barcodes with a minimal distance of two bases between any barcodes. The first two libraries were sheared using a Sonorex Super RK 102 P sonicator (Bandelin) for 2 minutes. The third library was sheared on an S220 series Adaptive Focused Acoustic (AFA) ultra-sonicator (Covaris) with the manufacturer’s settings for a 400 bp mean fragment size. Sheared fragments between 300–500 bp were size-selected on a 1.25% agarose gel. We carried out the enrichment step in four aliquots with 50 μl reaction volumes each, and combined these prior to the final size selection step. All three libraries were single-end sequenced on an Illumina HiSeq 2000 platform, yielding 136, 200 and 166 million 100 bp long reads, respectively. We sequenced each library on a single lane together with 7–20% bacteriophage PhiX genomic DNA (Illumina Inc.) to increase complexity at the first 10 sequenced base pairs. Sequencing was performed at the Center of Integrative Genomics (CIG), University of Lausanne and at the Next Generation Sequencing (NGS) Platform, University of Bern, Switzerland.

### Sequence data preparation, variant and genotype calling

We filtered raw sequencing reads from each lane and library for an intact *SbfI* restriction sites, de-multiplexed and barcode-trimmed them using the FASTX toolkit v.0.0.13 (http://hannonlab.cshl.edu/fastx_toolkit/) and custom python scripts. We aligned reads for each individual and library against the October 2013 re-assembly version of the threespine stickleback reference genome [26,77] using end-to-end alignment in Bowtie 2 v2.0.0 with default parameters [152]. SAMtools v0.1.19 [153] was used to convert alignments to binary format. We recalibrated base quality scores of aligned stickleback reads using empirical error rate estimations derived from bacteriophage PhiX reads. Raw sequencing reads from each lane were aligned against the PhiX 174 reference genome (accession: NC_001422; [154]), known variation was masked and PhiX-alignments were used to create a base quality score recalibration table for each lane and library combination using BaseRecalibrator from GATK v.2.7 [155]. We obtained between 0.9–2.5 billion base pairs of PhiX-reads per lane, sufficient to ensure good recalibration results. Using the GATK-tool PrintReads and PhiX-based recalibration tables, we then recalibrated base quality scores in stickleback alignments from the respective lanes.

We used the GATK tool UnifiedGenotyper to call variants and genotypes in a combined fashion for all individuals, using the following parameters: minimal phred-scaled base quality score threshold of 20, genotype likelihood model calling both SNPs and insertions/deletions (indels) and assumed contamination rate of 3%. Using custom python scripts and vcftools v0.1.12 [156], all genotypes with quality < 30 or depth < 10 were set to missing. Variants with quality < 30 or > 50% missing genotypes per sampling site, monomorphic sites, SNPs with > 2 alleles, indels and SNPs 10 bp around indels as well as SNPs from the sex chromosome XIX were removed from the dataset, the latter due to mapping and calling uncertainty in males. RAD-sequencing datasets contain PCR duplicate reads for a locus and individual, a well-known caveat of this technology [73–75,157,158], that cannot be identified in single-end sequencing data and can cause a bias towards calling homozygote genotypes when one allele of a heterozygote was by chance over-amplified [75]. We therefore additionally removed all sites that showed an excess of homozygotes, as measured by a significant deviation from Hardy-Weinberg equilibrium (p < 0.01) within any of the six populations using Arlequin v3.5.1.4 [159]. We noticed a higher prevalence of PCR duplicates in the first two libraries containing populations S1, L1 and S2, likely due to different shearing device used in the library preparation step. This is visible in elevated mean F_IS_ in these populations (see results section, Fig 2B). To reduce noise introduced by these PCR duplicates, we therefore randomly picked one allele per high-quality filtered genotype and used this ‘allele dataset’ in some of the analyses, while the high-quality filtered genotype dataset was used in analyses where we could account for an excess of homozygotes, i.e. for inbreeding. We used PGDSpider v2.0.5.0 [160] for conversion from VCF format to other formats.

### Population genomic analyses

We partitioned genomic variation in the allele dataset into principal components using adegenet [161], for sites with a minor allele frequency > 1%. We also performed Bayesian clustering assignment of individuals into one to five clusters using STRUCTURE v2.34. 10 [162], using the allele dataset with sites of greater than 1% minor allele frequency, following [163]. We ran 10 replicates assuming one to five clusters with 100,000 steps burn-in and 200,000 sampling steps and checked convergence of replicates visually. We identified the most likely number of clusters by the highest delta K statistics among the tested clusters [164].

We studied the genome-wide distribution of genetic differentiation by computing for each SNP F_ST_ estimates between pairs of sampling sites (‘pairwise F_ST_’, Fig 3) and among all sampling sites grouped hierarchically (‘hierarchical F_ST_’, S12A Fig). We used pairwise F_ST_ to characterize levels and heterogeneity of differentiation across the genome between pairs of populations, but we identified genomic islands of differentiation based on hierarchical F_ST_ in order to maximize the power to detect outlier SNPs, which were used to identify genomic islands of differentiation. SNP-level F-statistics (F_ST_, F_IT_ and F_IS_) were estimated in a locus-by-locus AMOVA in Arlequin v3.5.1.4 [159]. We characterized heterogeneity in genome-wide differentiation by calculating the mean, 95%-quantile and standard deviation of pairwise F_ST_’s in non-overlapping, 2 Mb-wide adjacent windows across the genome containing at least 20 SNPs. We defined heterogeneity in differentiation as the absolute coefficient of variation of these pairwise mean window F_ST_’s.

Single SNP hierarchical F_ST_ was estimated in a locus-by-locus AMOVA analysis in Arlequin, with populations grouped into three groups (stream 1, stream 2, lake) while maintaining the six sampling sites as separate populations. The grouping was based on genetic similarity between the sampling sites, assessed from genomic PCA (Fig 2A), mean weighted pairwise F_ST_ results (Fig 2B) and Bayesian clustering of individuals (S2 Fig). The first two, stream-like groups thus contained sites S1 and S2 respectively, and the third, lake-like group sites L1, L2, S1a and S1b (S2 Fig). In order to detect loci putatively under selection, we performed an outlier analysis based on a hierarchical island model [165]. This approach identified outlier SNPs by comparing observed hierarchical F_ST_ and heterozygosity values against a null distribution from a hierarchical island model, derived from 500,000 simulations of 10 groups with 100 demes each, as implemented in a modified version (v3.5.2.3) of Arlequin [165] (S12 Fig). Significantly positive population-specific F_IS_, potentially due to RAD sequencing PCR duplicates and leading to an apparent excess of homozygotes, were taken into account in the simulations used to build the joint null distribution of heterozygosity and F_ST_. In brief, for each simulated diploid individual the population-specific F_IS_ coefficient was used as the probability that the two gene copies present on homologous chromosomes were identical by descent or not. This procedure amounts at reducing the sample size by a factor 1-F_IS_ in the simulations, and thus to correctly take into account measured levels of inbreeding, which could either be due to true inbreeding or to PCR duplicates of a single chromosome. Our choices of group and deme size for simulating null distributions followed the recommendations of [165], who showed that reliable outlier probability estimation is obtained from simulations performed with numbers of groups and numbers of demes per group that exceed the actual (but unknown) numbers. We also ran the outlier analysis with different group / deme size combinations (3 groups / 4 demes, 3 / 10, 5 / 10, 50 / 10, 50 / 50) and found highly congruent outlier probabilities for each SNP (correlation coefficient r > 0.9999). We tested if outlier loci were randomly distributed on each chromosome by calculating Ripley’s K function following the approach by Flaxman et al. [7] accounting for recombination rate bias by using SNP positions on a genetic map (see section ‘genetic distances and recombination rates‘ below), with one modification: The null distribution of Ripley’s K was simulated by 10,000 times sampling n SNPs among all the SNPs in our dataset for the respective chromosome, not by drawing them from random positions in the genome [7], with n being the number of outliers on a chromosome. This was to avoid a bias in estimating expected values for Ripley’s K due to the non-random location of RAD-sequencing derived SNPs biased towards G/C-rich regions in the genome [151].

### Genomic islands of differentiation

We identified ‘genomic islands of differentiation’ following the approach of Hofer et al. [76] (S12 Fig). The HMM is based on three underlying and unobserved states, corresponding to ‘genomic background’ (assumed to be neutral under a hierarchical island model), regions of ‘exceptionally low’ differentiation, and regions of ‘exceptionally high’ differentiation. We refer to exceptionally high differentiation regions as ‘genomic islands of differentiation’. All three types of regions can consist of single SNPs or of several consecutive SNPs, depending on how outlier loci are clustered in the genome. The most likely state for each SNP is inferred from the HMM, based on its observed probability to be an outlier from the hierarchical F_ST_ analysis outlined above [76]. Subsequently, we retained only exceptional regions after multiple-testing correction with a false discovery rate of 0.001 for outlier loci [76]. Our approach differs in two aspects from [76]. First, we used only SNPs with minor allele frequencies > 1%. This minor allele frequency cutoff was not necessary for the data used by Hofer et al. [76], because they used ascertained SNPs. We found very low frequency allele SNPs to disrupt the detection of high differentiation levels, because they can never reach high differentiation and are thus less informative [166], even though they are naturally very abundant in unascertained sequence data. Second, we ran the HMM method for the concatenated SNP dataset instead of modeling every chromosome separately. This increased information for parameter estimation and did not affect the identification of islands of differentiation (i.e. no spurious islands of differentiation extending across chromosomes were identified).

Among genomic islands of differentiation identified by the HMM, we distinguished between islands showing parallel differentiation between both lake and stream stickleback breeding in sympatry and lake and stream stickleback breeding in parapatry and between islands of differentiation without parallel differentiation. We inferred parallel differentiation for each SNP by comparing allele counts between lake site L1 and the stream endpoint S1 as well as between lake site L2 and stream site S2. A parallel differentiation SNP had to show (a) parallel allele frequency change between habitats, i.e. the same allele had to be found at higher frequency in the same habitat in both comparisons and (b) the allele frequencies had to be significantly different in both lake-stream comparisons as assessed by a significant pairwise F_ST_ estimated in an AMOVA accounting for inbreeding levels as described above. We defined islands of parallel differentiation as islands containing at least one parallel differentiation SNP and computed a PCA with only those SNPs as described above. For all pairs of parallel differentiation SNPs, we estimated the extent of linkage disequilibrium within each sampling site from the absolute of the correlation coefficient between pairs of loci (|r|) based on genotype counts using PLINK v1.07 [167]. For all genomic islands of differentiation, we counted the number of SNPs showing significantly different allele frequencies in sympatry (L1 vs. S1) and in parapatry (L2 vs. S2) also assessed by a significant pairwise F_ST_ between these populations (S1 Table).

Nucleotide diversity in each population was calculated using one allele per high-quality genotype with quality > 30, depth > 10 and maximal 50% missing data, excluding sites located within 10 bp from indels or sites on the sex chromosome XIX. These filtered sites were partitioned into windows of variable size containing at least 2,500 sequenced sites, without splitting single RAD sequence reads, resulting in a mean window size of 324,800 bp (median 302,900 bp, range 58,960–1,036,000 bp). Arlequin v3.5.2.3 [165] was used to calculate nucleotide diversity for each window in each population. Windows were checked for the presence of parallel and non-parallel islands and labelled as ‘genomic background’, ‘parallel island’ and ‘non-parallel island’ windows accordingly (Fig 6). Within each population, we tested for differences in mean nucleotide diversity between parallel island, non-parallel island and genomic background windows using t-tests and Bonferroni-based multiple comparison adjusted p-values.

### Genetic distances and recombination rate

We derived genetic distances and recombination rates from a previously published recombination map based on a cross between threespine stickleback from Lake Constance and Lake Geneva, Switzerland [77]. Position along the genetic map for each SNP was estimated by linear interpolation of genetic vs. physical positions as published in [77]. We estimated the regional recombination rate around each SNP in our dataset by smoothing the genetic vs. physical map [77] with cubic splines and a spline parameter of 0.7 for each chromosome and calculating the smoothed curve’s first derivate [168]. We used non-parametric tests to find correlations between recombination rate and the presence of islands of differentiation (Kruskal-Wallis test), hierarchical, and pairwise differentiation (F_ST_, Spearman-rank correlations) and assessed significance with a Bonferroni-corrected alpha level of 0.05.

### Identification of putative targets of selection

We studied the overlap of islands of parallel differentiation and previously identified QTL, candidate genes, expression outliers and outlier regions: We assembled a database of previously identified QTL in threespine stickleback from the literature published up to mid-2015 [78–103]. If reported, 95% confidence intervals were directly taken from the literature or the markers in the genetic map of the study adjacent to the ‘peak LOD score minus 1.5’ boundaries on both sides of the LOD peak were used as 95% confidence intervals. In studies where only the highest-scoring markers were reported, we used the marker ± 1 Mb as approximate QTL confidence intervals. Physical positions of QTL and confidence interval estimates were transformed into October 2013 stickleback re-assembly coordinates [77] using the UCSC tool liftOver [169] and corresponding positions along the genetic map were calculated as for SNPs (see above). We then tested if QTLs grouped into 32 traits and genomic islands of parallel differentiation overlap, using a buffer of ±10 kbp on both sides of genomic islands to alleviate effects of sparse SNP sampling by RAD sequencing (also applied in all following overlap analyses). We tested if overlaps were expected by chance by permuting the physical and genetic positions of these islands 100,000 times randomly across the genome, re-calculating overlaps and deriving empirical null distributions and p-values for the observed number of overlaps with a Bonferroni-corrected alpha level of 0.05, based on the repeated testing for overlaps with 32 traits.

We further examined gene content of genomic islands of differentiation and their overlap with previously identified candidate genes for divergent adaptation [88,126,128,132] and expression outliers [129,131], for which full gene lengths and a buffer of ± 10 kbp sequence on both sides of each gene were used. The set of overlapping genes was tested for enrichment in gene ontology (GO) terms for the GO categories ‘biological processes’ and ‘molecular functions’ using the STRING v9.1 database [170], applying a Bonferroni-corrected alpha level of 0.05. Finally, we overlapped genomic islands of parallel differentiation from our study with previously identified outlier markers [25,53,70,124,125,127,130] or outlier regions [24,26,36], of which physical locations were publicly available. We used either the exact outlier region if reported [26,36], an approximation of an outlier region based on its reported content ± 100 kbp sequence on both sides [24], the ± 100 kbp region surrounding a reported outlier marker for high-density SNP data [53,70] or the ± 1 Mb region surrounding an outlier marker for low-density microsatellite datasets [25,124,125,127,130] for comparison with our genomic islands of differentiation. Statistical analyses and plotting was done using R v3.0.1 [171]. Data analysis was conducted using the bioinformatics infrastructure of the Genetic Diversity Centre (GDC), ETH Zurich/Eawag.

## Supporting Information

S1 Fig Timing of threespine stickleback breeding season at Lake Site L1 and Stream Site S1 in 2009. Stickleback start breeding at the same time at sites L1 and S1, preliminarily suggesting synchronous reproduction in sympatry and thus the absence of temporal isolation. Note however that both lake and stream ecotypes not distinguished in this dataset may occur at site S1. Furthermore, we lack information on the length of breeding seasons of lake and stream ecotypes each at these sites, leaving the possibility for partial temporal isolation.
(TIF)

S2 Fig Bayesian clustering of Lake Constance threespine stickleback. Assignment of all 91 individuals to 2–5 clusters, based on the 13,509 SNP allele dataset with a minor allele frequency of > 1%, using the Bayesian clustering algorithm STRUCTURE [162]. According to the optimality criterion developed by Evanno et al. [164], three clusters best fit the data. Grey boxes around x-axis labels show the grouping of sampling sites used for the hierarchical outlier analysis (see Materials & Methods).
(TIF)

S3 Fig Optimal number of clusters from Bayesian clustering. Estimated likelihoods and likelihood derivatives for different numbers of clusters based on 10 replicate runs per cluster number of the Bayesian clustering algorithm STRUCTURE [162]. Three clusters best fit the data according to Evanno et al. [164].
(TIF)

S4 Fig Genome-wide distribution of pairwise differentiation (F_ST_). Pairwise F_ST_ distributions across the genome for the comparisons between pairs of sampling sites not already shown in Fig 3. Note striking differentiation on chromosome VII between sites dominated by stream ecotypes versus sites with mostly lake ecotypes **(A, B, D, E, H, I)** and the absence of differentiation between sites both dominated by lake ecotypes **(C, F, G, J)**. Grey dots show single SNP pairwise F_ST_ estimates and black lines show F_ST_ means (bold) and 95%-quantiles (thin) in 2 Mb wide, non-overlapping windows across the genome. Windows with elevated differentiation are highlighted with blue background frames (mean F_ST_ > 0.05) and red background bars (95%-quantile F_ST_ > 0.25).
(TIF)

S5 Fig Test for clustering of outlier SNPs per chromosome. For each separate chromosome with more than 2 outlier SNPs, Ripley’s K function is plotted, for outlier SNPs (red line, alpha-level 5%) and for the neutral model of loci without clustering, where median, 95% and 99% confidence intervals are shown (blue lines, see Materials & Methods). Chromosomes for which the red line crosses blue confidence intervals show evidence for clustering of outliers beyond expectations from recombination rate.
(TIF)

S6 Fig Detail view of genomic islands of differentiation and QTLs on chromosome VII. **(B)** Chromosome VII contains 12 genomic islands of parallel differentiation (IPDs, black vertical bars) and two islands of non-parallel differentiation (INDs, grey vertical bars). **(A)** QTLs for traits previously studied among Lake Constance ecotypes and their overlap with parallel islands are shown. The left grey column indicates if traits have previously been found to be divergent among Lake Constance ecotypes (‘Y’ = yes) or not (‘N’ = no). Significant clustering of parallel islands inside QTLs for trait groups are indicated by asterisks in the right grey column. Blocks indicate 95% QTL confidence intervals (extent along x-axis) and effect sizes (color). References for phenotypic data: ^1^[59], ^2^[57], ^3^[65], ^4^[56] and S7B Fig, ^5^[46], ^6^S7A Fig. **(C)** Recombination rates across the stickleback genome as estimated by Roesti et al. [77] are visualized.
(TIF)

S7 Fig Ecotype differences in lateral plate cover but not lateral plate morph among Lake Constance ecotypes. **(A)** First axis of a PCA of size-corrected lateral plate height data from lake and stream ecotypes from sampling sites S1, S2, L1 and L2, showing that lateral plate height differs among lake and stream ecotypes in Lake Constance (ANOVA, F_1,50_ = 7.52, p < 0.009), with lake ecotypes having higher lateral plate cover (Fig 1B). **(B)** Lake and stream ecotypes from sampling sites S1, S2, L1 and L2 however do not differ in plate morph (χ^2^_2_ = 1.76, p = 0.41), with most fish being fully-plated (FP) and few individuals being partially plated (PP) and low plated (LP).
(TIF)

S8 Fig Distribution of genotypes in genomic islands of parallel lake-stream differentiation across the six sampling sites. In the sites S1a and S1b, both individuals with lake-like genotypes and others with stream-like genotypes occur, as well as more intermediate / admixed individuals. Columns show the same parallel lake-stream differentiation SNPs in islands of parallel differentiation as in Fig 5, with the color code for stream- (light blue) and lake-like alleles (dark blue). The grey left column shows the Bayesian clustering assignment of individuals to K = 3 clusters (see S2 Fig).
(TIF)

S9 Fig Heat map for linkage disequilibrium (LD) within sampling sites between parallel lake-stream differentiation SNPs. The pattern of LD between SNPs found in genomic islands of lake-stream differentiation and showing parallel changes in allele frequencies is revealed by the absolute value of the correlation coefficient r, a classical measure of LD. Different islands of differentiation are divided by either white or black vertical and horizontal lines, the black lines also dividing different chromosomes. SNPs are grouped by parallel islands as in Figs 5 and S8.
(TIF)

S10 Fig Probability of overlap between genomic islands of parallel differentiation and 32 QTL categories. Probability distributions from 100’000 random permutations of the 19 islands of parallel differentiation on the genetic map and their overlap with QTL from different trait categories (grey histograms), as well as the observed overlap between islands and QTL and associated p-values (black arrows). P-values significant after Bonferroni correction for multiple testing are shown in bold.
(TIF)

S11 Fig Genomic islands of parallel differentiation and their overlap with QTLs. QTLs for traits that have not yet been studied in Lake Constance ecotypes and their overlap with genomic islands of parallel differentiation. Significant overlap with parallel genomic islands is indicated by asterisks in the right grey column. Blocks indicate 95% QTL confidence intervals (range along x-axis) and effect sizes (color) respectively.
(TIF)

S12 Fig Islands of differentiation identification using a hierarchical outlier analysis and Hidden Markov Model (HMM) approach. **(A)** Results from an outlier analysis under a hierarchical island model [165] showing all SNPs colored according to their associated p-value, i.e. the probability of the observed F_ST_ under neutrality. The SNP p-value color coding is the same in all three plots, and the scale is shown in pane B. H_BP_: observed heterozygosity between populations. **(B)** Z-transformed p-values from the outlier analysis (z-scores, see histogram) of SNPs with minor allele frequency > 1% are used in parameter estimation for an HMM with three states of genomic differentiation [76]: genomic background differentiation (grey line), exceptionally low differentiation (green line) and exceptionally high differentiation (orange line). The lines show the normally distributed emission probabilities in the HMM for each state (see Materials and Methods). **(C)** Example for the inference of genomic islands of differentiation: regions identified as genomic background differentiation are shown with a grey background and regions of genomic islands of differentiation (i.e. regions with exceptionally high differentiation) are shown with an orange background.
(TIF)

S1 Table Genomic islands of differentiation (DIFF) and parallel differentiation (PARDIFF) and SNP counts. (DOCX)

S2 Table Associations between recombination rate and genomic islands of differentiation, parallel (IPD), non-parallel (IND) islands and SNP F_ST_ estimates. (DOCX)

S3 Table Previously identified QTLs and outlier regions overlapping with genomic islands of parallel differentiation. (DOCX)

S4 Table Ensembl predicted genes overlapping with islands of parallel differentiation among Lake Constance lake and stream ecotypes. (DOCX)
