# Supplementary figures and images for: Genomics of Rapid Incipient Speciation in Sympatric Threespine Stickleback
Source: PLoS Genet. 2016 Feb 29;12(2):e1005887. doi: 10.1371/journal.pgen.1005887 (PMC4771382; doi:10.1371/journal.pgen.1005887)

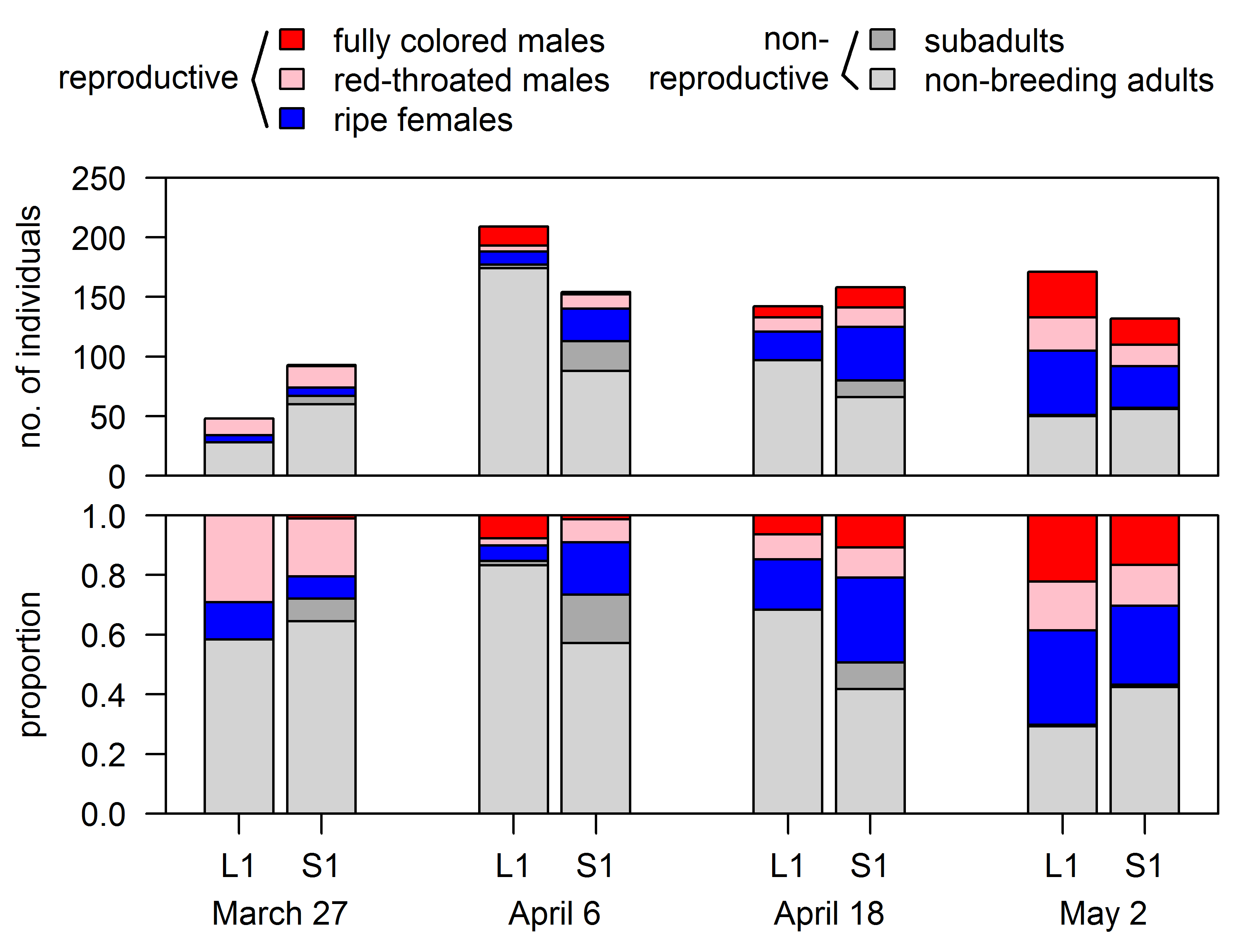

Supplement: S1 Fig — Stickleback start breeding at the same time at sites L1 and S1, preliminarily suggesting synchronous reproduction in sympatry and thus the absence of temporal isolation. Note however that both lake and stream ecotypes not distinguished in this dataset may occur at site S1. Furthermore, we lack information on the length of breeding seasons of lake and stream ecotypes each at these sites, leaving the possibility for partial temporal isolation. (TIF) [file pgen.1005887.s001.tif]

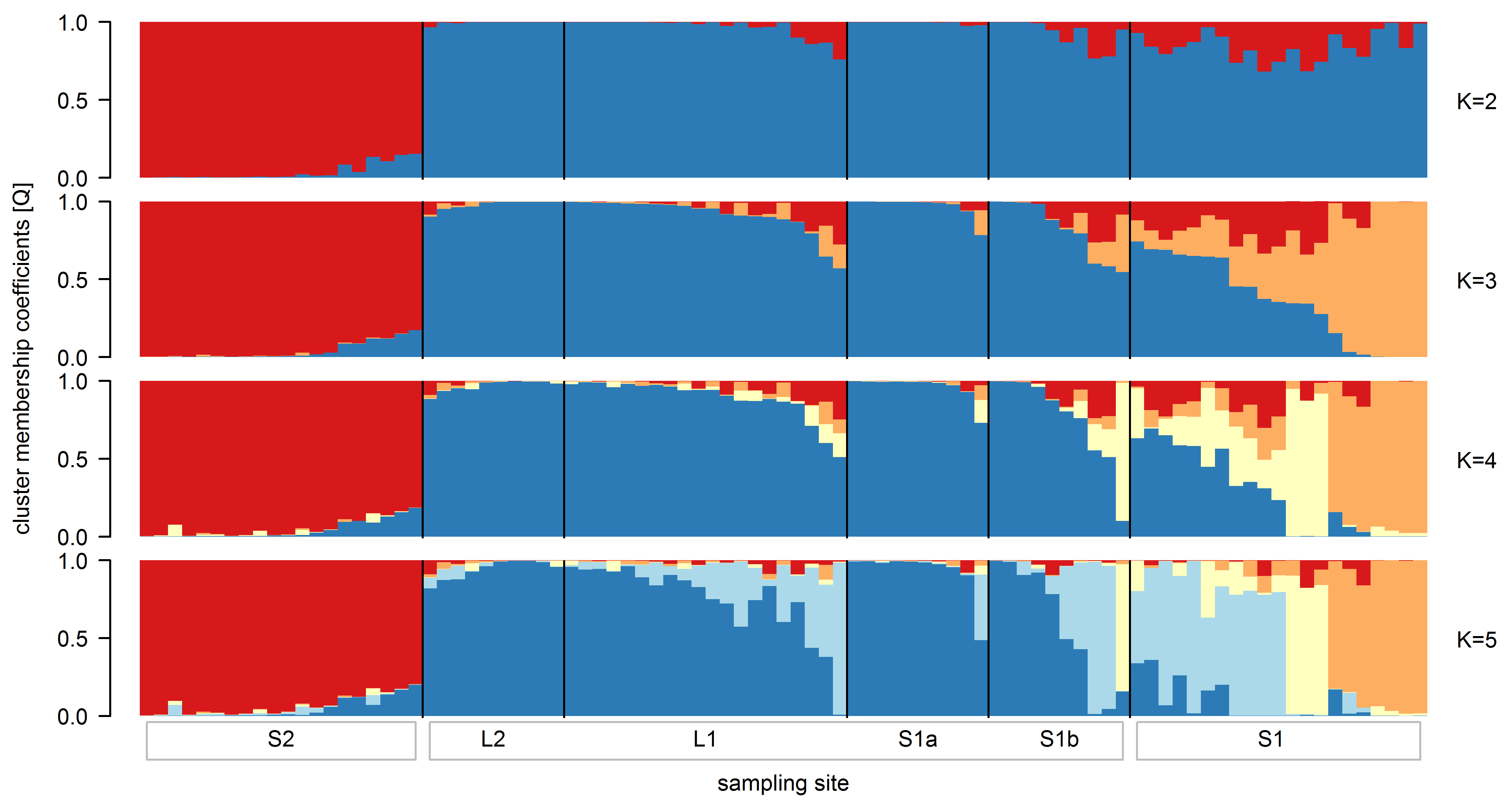

Supplement: S2 Fig — Assignment of all 91 individuals to 2–5 clusters, based on the 13,509 SNP allele dataset with a minor allele frequency of > 1%, using the Bayesian clustering algorithm STRUCTURE [162]. According to the optimality criterion developed by Evanno et al. [164], three clusters best fit the data. Grey boxes around x-axis labels show the grouping of sampling sites used for the hierarchical outlier analysis (see Materials & Methods). (TIF) [file pgen.1005887.s002.tif]

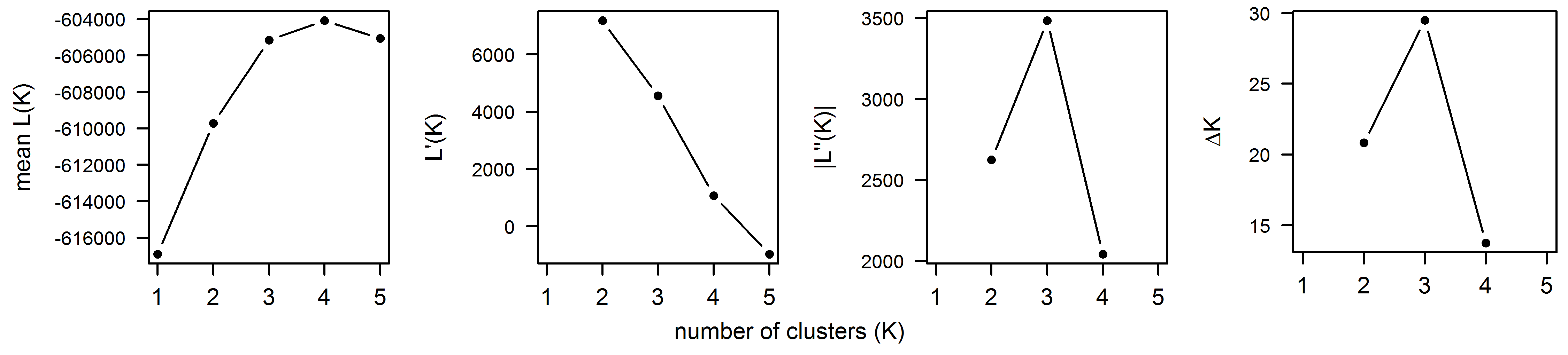

Supplement: S3 Fig — Estimated likelihoods and likelihood derivatives for different numbers of clusters based on 10 replicate runs per cluster number of the Bayesian clustering algorithm STRUCTURE [162]. Three clusters best fit the data according to Evanno et al. [164]. (TIF) [file pgen.1005887.s003.tif]

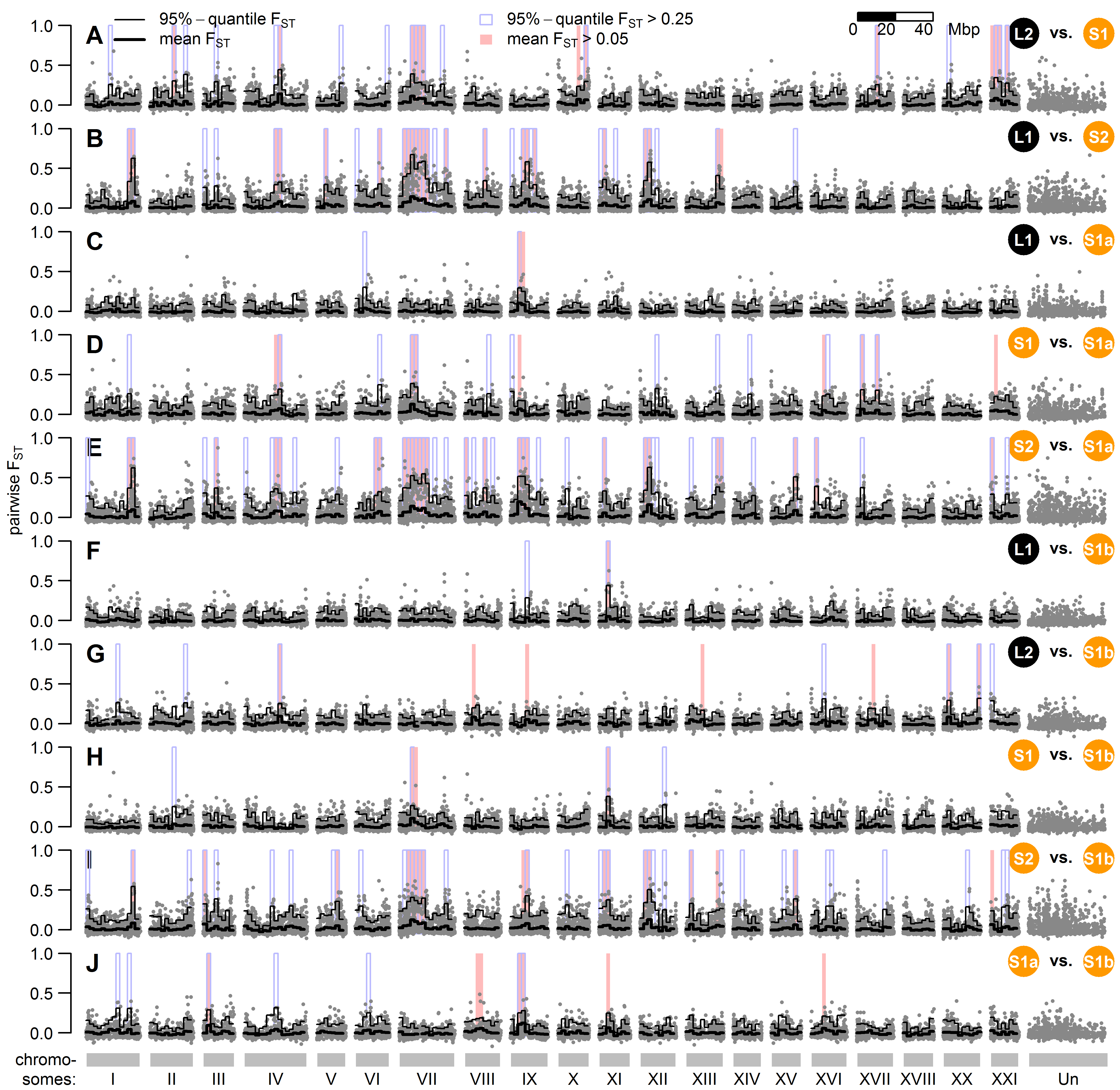

Supplement: S4 Fig — Pairwise FST distributions across the genome for the comparisons between pairs of sampling sites not already shown in Fig 3. Note striking differentiation on chromosome VII between sites dominated by stream ecotypes versus sites with mostly lake ecotypes (A, B, D, E, H, I) and the absence of differentiation between sites both dominated by lake ecotypes (C, F, G, J). Grey dots show single SNP pairwise FST estimates and black lines show FST means (bold) and 95%-quantiles (thin) in 2 Mb wide, non-overlapping windows across the genome. Windows with elevated differentiation are highlighted with blue background frames (mean FST > 0.05) and red background bars (95%-quantile FST > 0.25). (TIF) [file pgen.1005887.s004.tif]

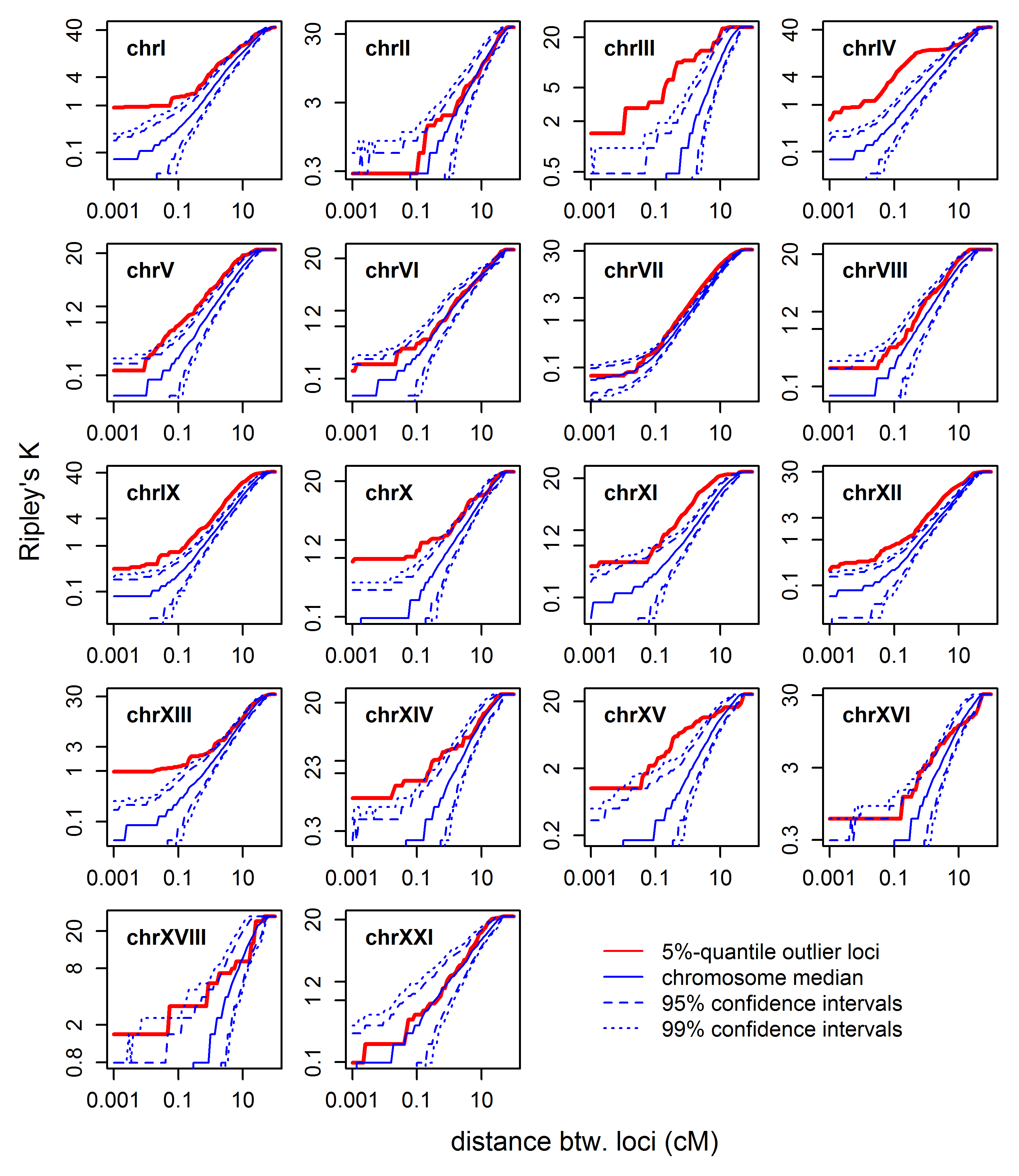

Supplement: S5 Fig — For each separate chromosome with more than 2 outlier SNPs, Ripley’s K function is plotted, for outlier SNPs (red line, alpha-level 5%) and for the neutral model of loci without clustering, where median, 95% and 99% confidence intervals are shown (blue lines, see Materials & Methods). Chromosomes for which the red line crosses blue confidence intervals show evidence for clustering of outliers beyond expectations from recombination rate. (TIF) [file pgen.1005887.s005.tif]

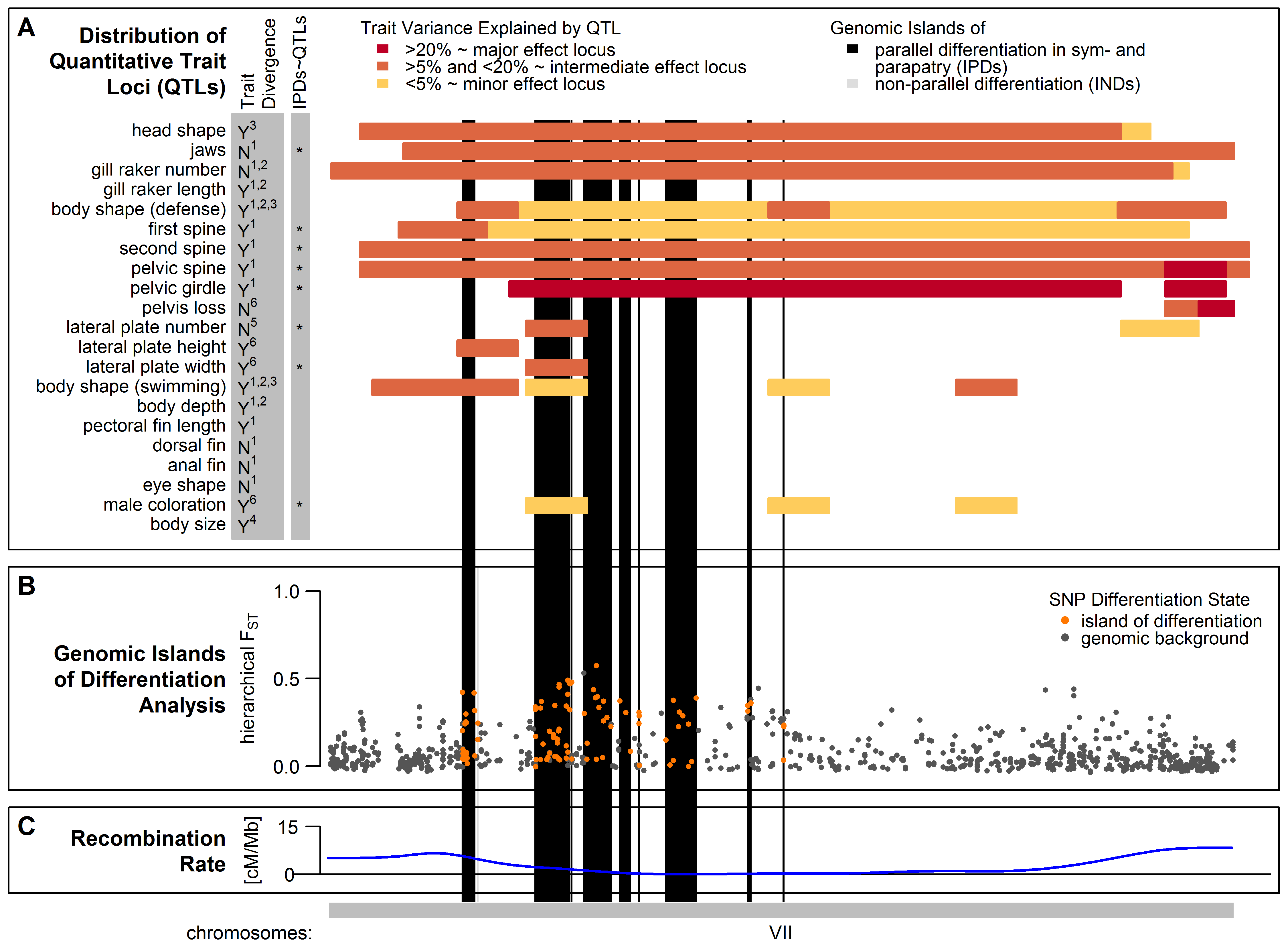

Supplement: S6 Fig — (B) Chromosome VII contains 12 genomic islands of parallel differentiation (IPDs, black vertical bars) and two islands of non-parallel differentiation (INDs, grey vertical bars). (A) QTLs for traits previously studied among Lake Constance ecotypes and their overlap with parallel islands are shown. The left grey column indicates if traits have previously been found to be divergent among Lake Constance ecotypes (‘Y’ = yes) or not (‘N’ = no). Significant clustering of parallel islands inside QTLs for trait groups are indicated by asterisks in the right grey column. Blocks indicate 95% QTL confidence intervals (extent along x-axis) and effect sizes (color). References for phenotypic data: 1[59], 2[57], 3[65], 4[56] and S7B Fig, 5[46], 6S7A Fig. (C) Recombination rates across the stickleback genome as estimated by Roesti et al. [77] are visualized. (TIF) [file pgen.1005887.s006.tif]

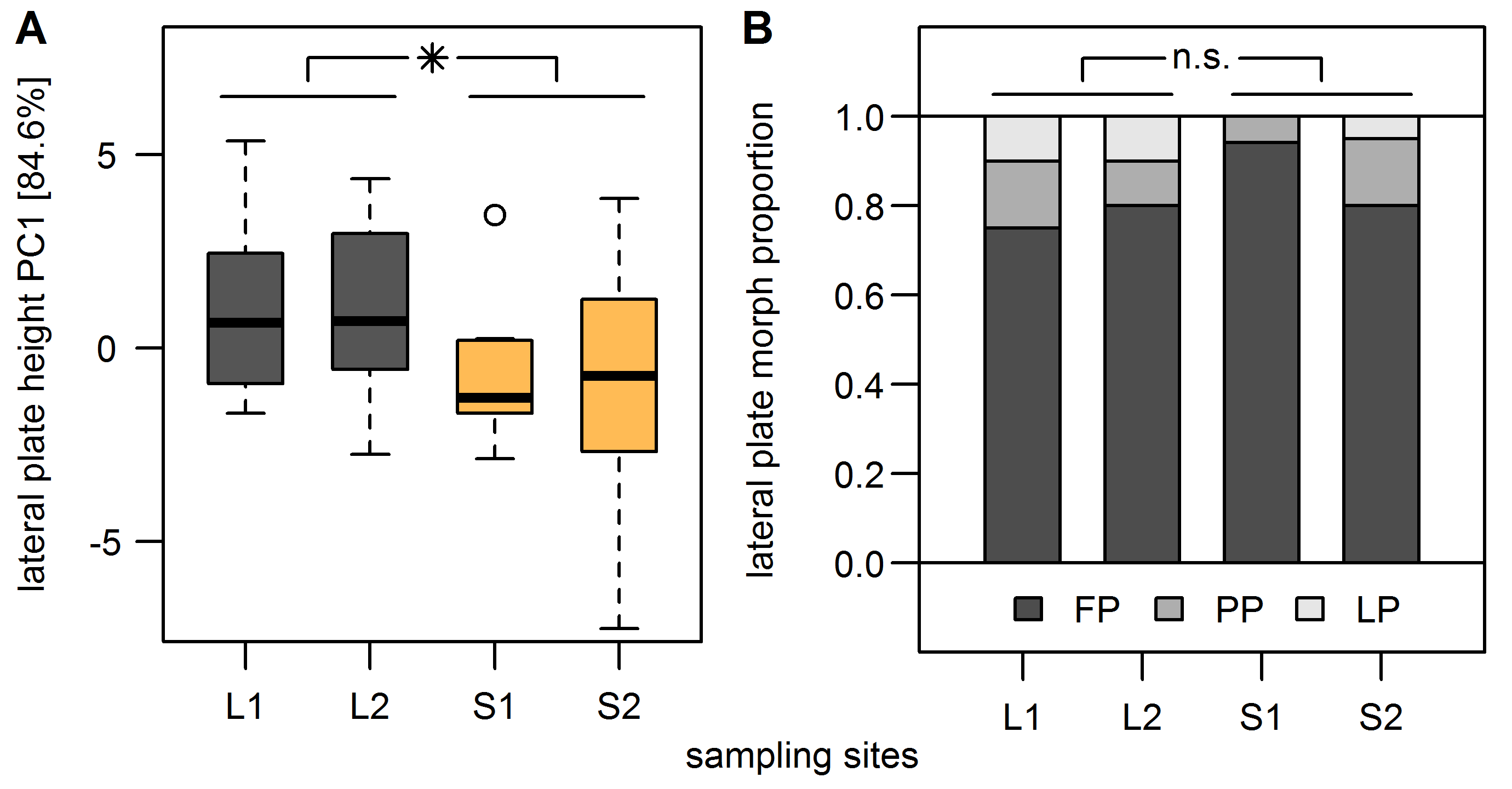

Supplement: S7 Fig — (A) First axis of a PCA of size-corrected lateral plate height data from lake and stream ecotypes from sampling sites S1, S2, L1 and L2, showing that lateral plate height differs among lake and stream ecotypes in Lake Constance (ANOVA, F1,50 = 7.52, p < 0.009), with lake ecotypes having higher lateral plate cover (Fig 1B). (B) Lake and stream ecotypes from sampling sites S1, S2, L1 and L2 however do not differ in plate morph (χ22 = 1.76, p = 0.41), with most fish being fully-plated (FP) and few individuals being partially plated (PP) and low plated (LP). (TIF) [file pgen.1005887.s007.tif]

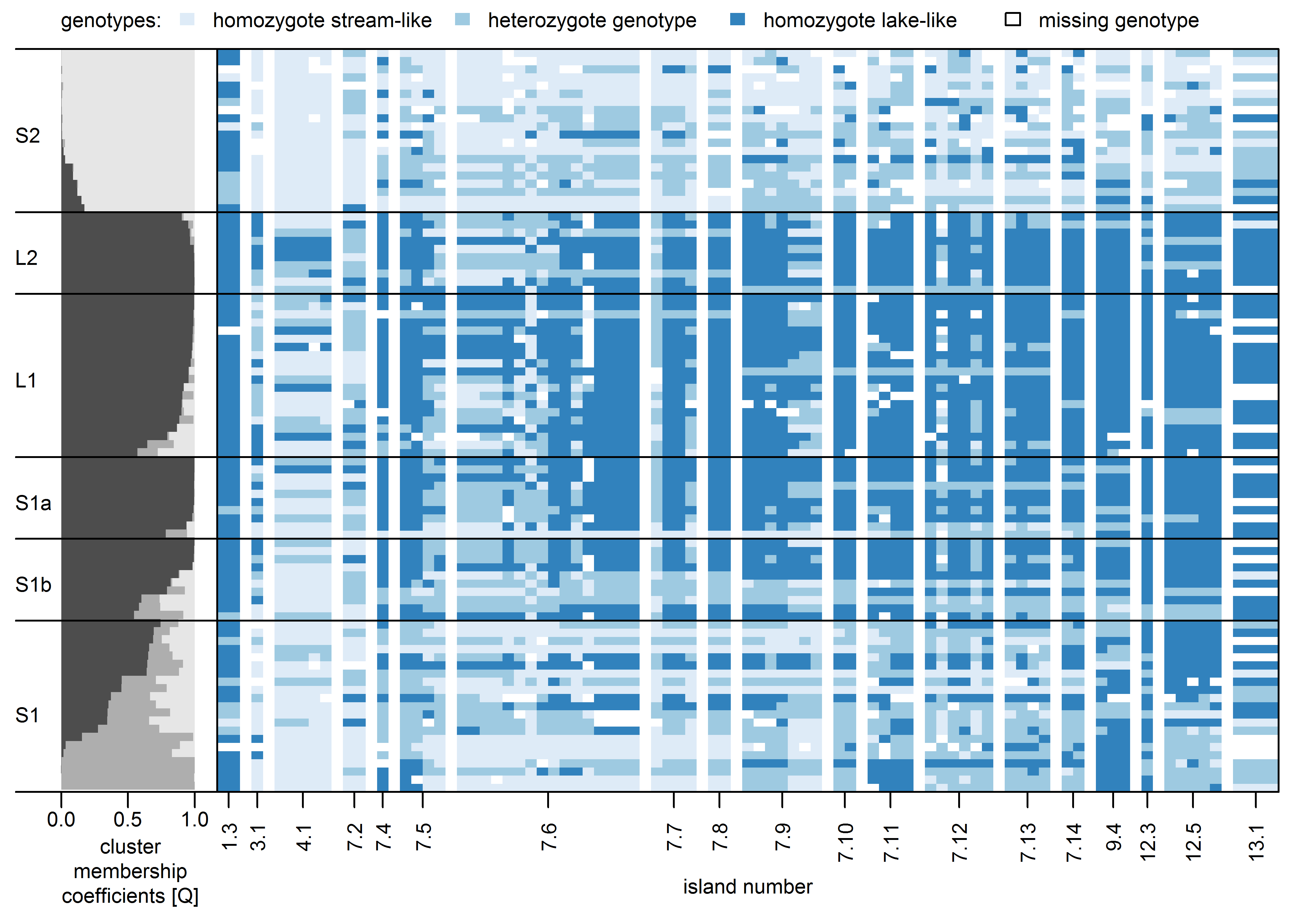

Supplement: S8 Fig — In the sites S1a and S1b, both individuals with lake-like genotypes and others with stream-like genotypes occur, as well as more intermediate / admixed individuals. Columns show the same parallel lake-stream differentiation SNPs in islands of parallel differentiation as in Fig 5, with the color code for stream- (light blue) and lake-like alleles (dark blue). The grey left column shows the Bayesian clustering assignment of individuals to K = 3 clusters (see S2 Fig). (TIF) [file pgen.1005887.s008.tif]

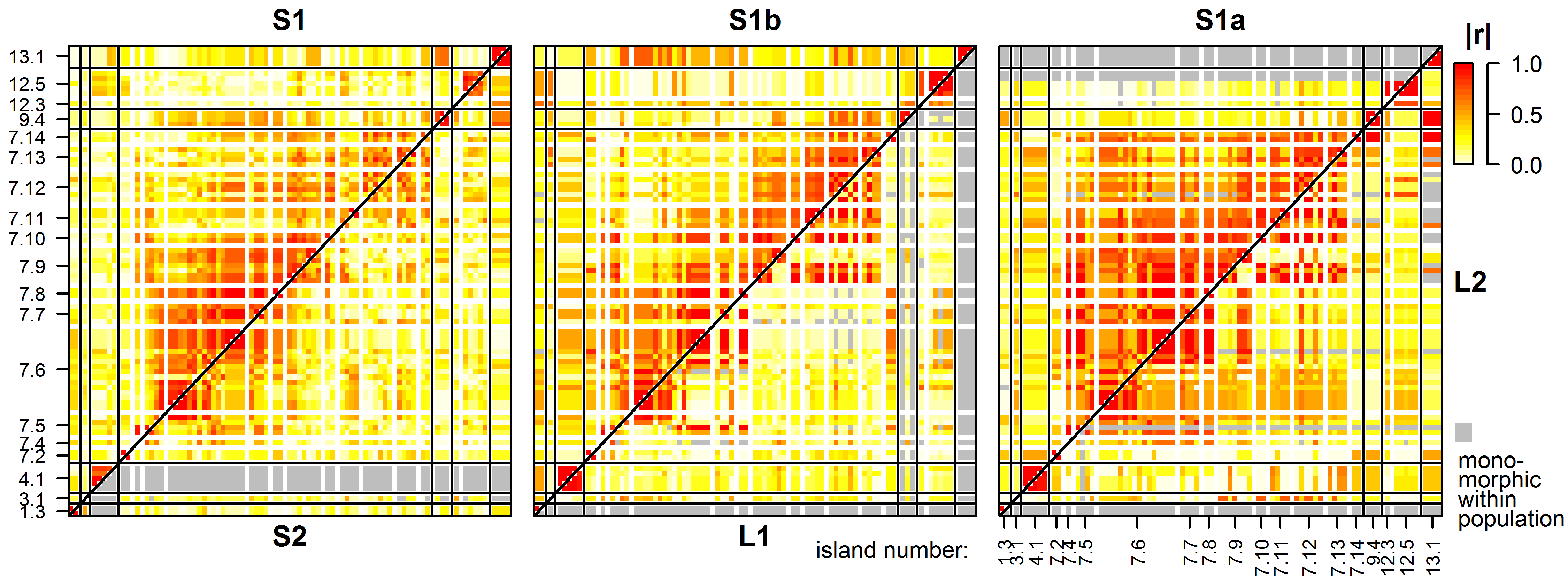

Supplement: S9 Fig — The pattern of LD between SNPs found in genomic islands of lake-stream differentiation and showing parallel changes in allele frequencies is revealed by the absolute value of the correlation coefficient r, a classical measure of LD. Different islands of differentiation are divided by either white or black vertical and horizontal lines, the black lines also dividing different chromosomes. SNPs are grouped by parallel islands as in Figs 5 and S8. (TIF) [file pgen.1005887.s009.tif]

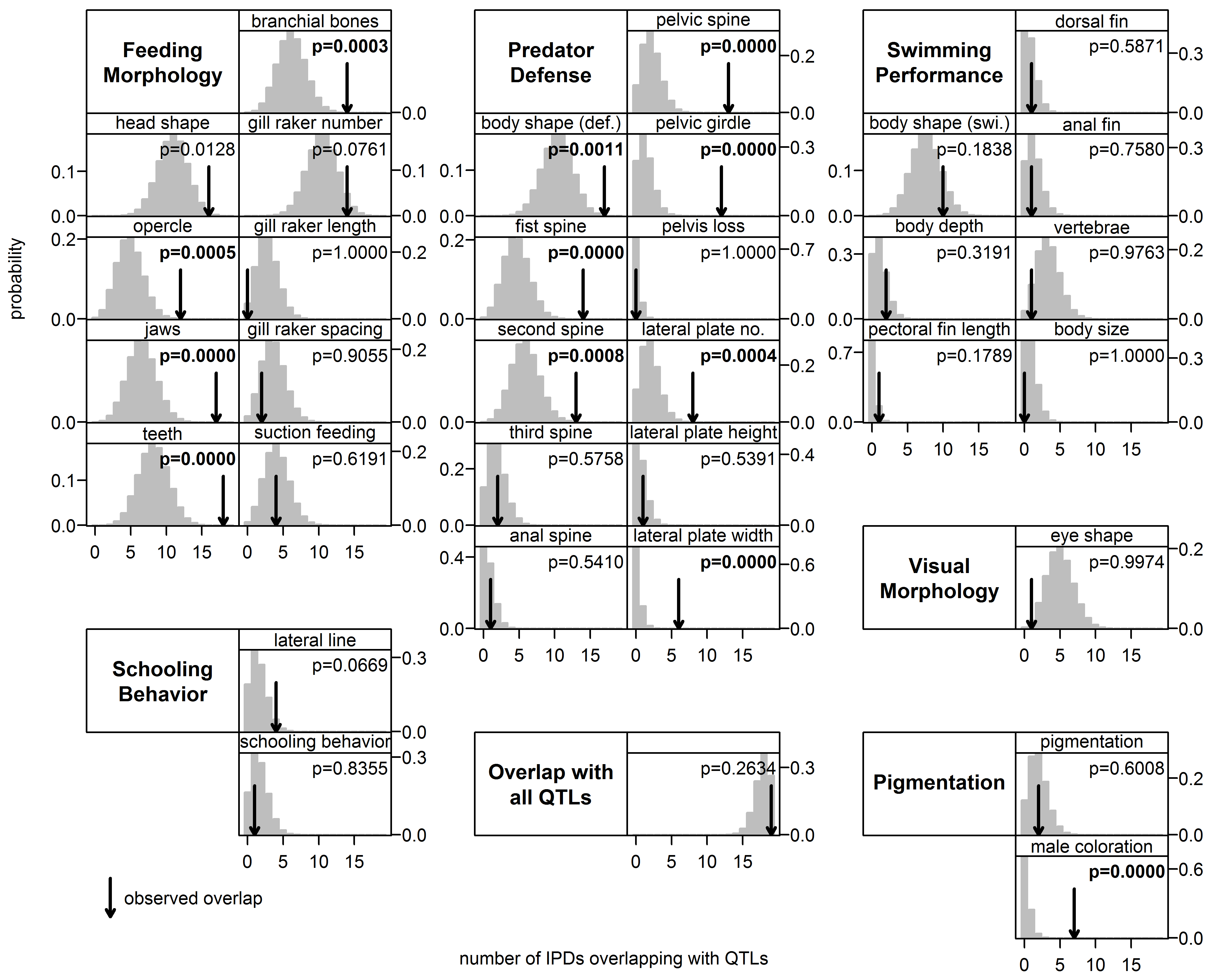

Supplement: S10 Fig — Probability distributions from 100’000 random permutations of the 19 islands of parallel differentiation on the genetic map and their overlap with QTL from different trait categories (grey histograms), as well as the observed overlap between islands and QTL and associated p-values (black arrows). P-values significant after Bonferroni correction for multiple testing are shown in bold. (TIF) [file pgen.1005887.s010.tif]

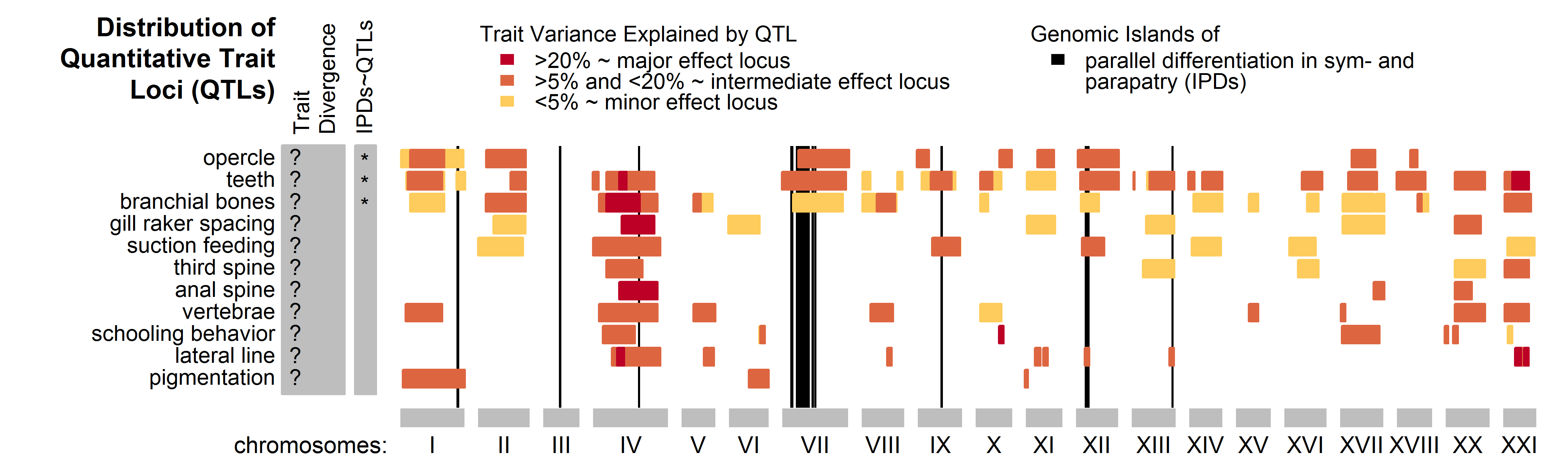

Supplement: S11 Fig — QTLs for traits that have not yet been studied in Lake Constance ecotypes and their overlap with genomic islands of parallel differentiation. Significant overlap with parallel genomic islands is indicated by asterisks in the right grey column. Blocks indicate 95% QTL confidence intervals (range along x-axis) and effect sizes (color) respectively. (TIF) [file pgen.1005887.s011.tif]

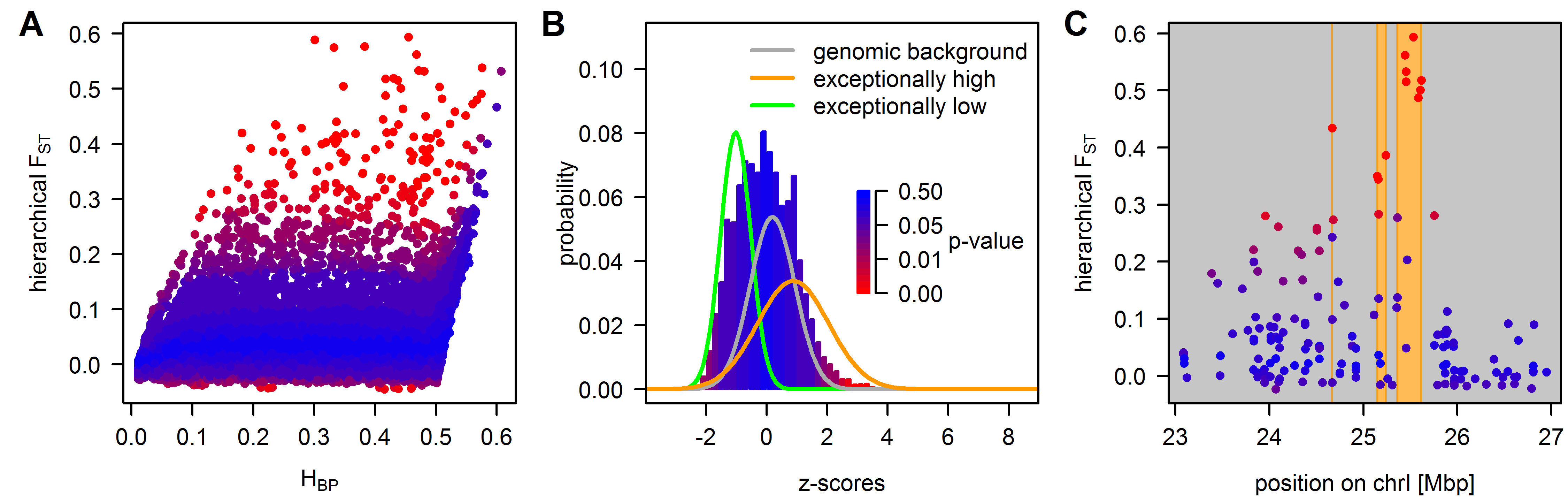

Supplement: S12 Fig — (A) Results from an outlier analysis under a hierarchical island model [165] showing all SNPs colored according to their associated p-value, i.e. the probability of the observed FST under neutrality. The SNP p-value color coding is the same in all three plots, and the scale is shown in pane B. HBP: observed heterozygosity between populations. (B) Z-transformed p-values from the outlier analysis (z-scores, see histogram) of SNPs with minor allele frequency > 1% are used in parameter estimation for an HMM with three states of genomic differentiation [76]: genomic background differentiation (grey line), exceptionally low differentiation (green line) and exceptionally high differentiation (orange line). The lines show the normally distributed emission probabilities in the HMM for each state (see Materials and Methods). (C) Example for the inference of genomic islands of differentiation: regions identified as genomic background differentiation are shown with a grey background and regions of genomic islands of differentiation (i.e. regions with exceptionally high differentiation) are shown with an orange background. (TIF) [file pgen.1005887.s012.tif]
