# Supplementary material for: Genomics of Rapid Incipient Speciation in Sympatric Threespine Stickleback
Source: PLoS Genet. 2016 Feb 29;12(2):e1005887. doi: 10.1371/journal.pgen.1005887 (PMC4771382; doi:10.1371/journal.pgen.1005887)
Supplement: S1 Table — (DOCX) [file pgen.1005887.s013.docx]

| ID | Region type | Chrom. | Start | End | Length | Number of SNPs | | | |
| --- | --- | --- | --- | --- | --- | --- | --- | --- | --- |
|  |  |  |  |  |  | All | Parallel | Sympat. | Parapat. |
| 1.3 | PARDIFF | chrI | 25,362,663 | 25,616,857 | 254,195 | 17 | 2 | 2 | 16 |
| 3.1 | PARDIFF | chrIII | 7,882,254 | 7,974,761 | 92,508 | 6 | 1 | 2 | 3 |
| 4.1 | PARDIFF | chrIV | 20,376,166 | 20,400,006 | 23,841 | 6 | 5 | 5 | 5 |
| 7.2 | PARDIFF | chrVII | 4,413,074 | 4,799,436 | 386,363 | 18 | 2 | 3 | 10 |
| 7.4 | PARDIFF | chrVII | 6,786,309 | 6,790,366 | 4,058 | 3 | 1 | 1 | 2 |
| 7.5 | PARDIFF | chrVII | 6,790,375 | 7,240,394 | 450,020 | 11 | 4 | 5 | 4 |
| 7.6 | PARDIFF | chrVII | 7,251,861 | 7,848,833 | 596,973 | 26 | 16 | 19 | 16 |
| 7.7 | PARDIFF | chrVII | 7,848,863 | 7,907,522 | 58,660 | 4 | 4 | 4 | 4 |
| 7.8 | PARDIFF | chrVII | 7,974,059 | 7,974,111 | 53 | 3 | 2 | 2 | 2 |
| 7.9 | PARDIFF | chrVII | 8,390,237 | 9,261,527 | 871,291 | 15 | 7 | 8 | 11 |
| 7.10 | PARDIFF | chrVII | 9,549,829 | 9,899,121 | 349,293 | 3 | 2 | 3 | 2 |
| 7.11 | PARDIFF | chrVII | 10,175,075 | 10,190,161 | 15,087 | 5 | 4 | 4 | 4 |
| 7.12 | PARDIFF | chrVII | 11,063,706 | 12,054,129 | 990,424 | 12 | 6 | 6 | 6 |
| 7.13 | PARDIFF | chrVII | 13,737,353 | 13,841,257 | 103,905 | 4 | 4 | 4 | 4 |
| 7.14 | PARDIFF | chrVII | 14,912,234 | 14,919,831 | 7,598 | 3 | 2 | 2 | 2 |
| 9.4 | PARDIFF | chrIX | 10,751,244 | 10,865,023 | 113,780 | 5 | 3 | 3 | 5 |
| 12.3 | PARDIFF | chrXII | 4,659,618 | 5,081,461 | 421,844 | 13 | 1 | 2 | 5 |
| 12.5 | PARDIFF | chrXII | 5,650,741 | 5,974,745 | 324,005 | 12 | 5 | 6 | 11 |
| 13.1 | PARDIFF | chrXIII | 18,199,528 | 18,199,671 | 144 | 5 | 4 | 5 | 4 |
| 1.1 | DIFF | chrI | 24,667,909 | 24,667,915 | 7 | 2 | 0 | 0 | 2 |
| 1.2 | DIFF | chrI | 25,146,091 | 25,238,004 | 91,914 | 9 | 0 | 0 | 6 |
| 2.1 | DIFF | chrII | 20,380,662 | 20,380,740 | 79 | 4 | 0 | 0 | 3 |
| 5.1 | DIFF | chrV | 10,909,516 | 10,909,520 | 5 | 2 | 0 | 0 | 2 |
| 7.1 | DIFF | chrVII | 4,395,898 | 4,399,876 | 3,979 | 3 | 0 | 1 | 2 |
| 7.3 | DIFF | chrVII | 4,905,227 | 4,905,271 | 45 | 2 | 0 | 0 | 1 |
| 9.1 | DIFF | chrIX | 5,363,550 | 5,363,550 | 1 | 1 | 0 | 0 | 1 |
| 9.2 | DIFF | chrIX | 8,658,452 | 9,074,143 | 415,692 | 5 | 0 | 0 | 4 |
| 9.3 | DIFF | chrIX | 10,120,516 | 10,372,039 | 251,524 | 4 | 0 | 0 | 3 |
| 11.1 | DIFF | chrXI | 4,051,398 | 4,913,659 | 862,262 | 16 | 0 | 0 | 8 |
| 11.2 | DIFF | chrXI | 4,950,911 | 5,068,446 | 117,536 | 3 | 0 | 0 | 1 |
| 12.1 | DIFF | chrXII | 4,452,809 | 4,473,331 | 20,523 | 4 | 0 | 0 | 2 |
| 12.2 | DIFF | chrXII | 4,616,721 | 4,627,756 | 11,036 | 3 | 0 | 0 | 1 |
| 12.4 | DIFF | chrXII | 5,233,072 | 5,389,222 | 156,151 | 8 | 0 | 0 | 6 |
| 12.6 | DIFF | chrXII | 6,451,608 | 6,451,608 | 1 | 1 | 0 | 0 | 1 |
| 12.7 | DIFF | chrXII | 7,309,056 | 7,309,056 | 1 | 1 | 0 | 0 | 1 |
| 13.2 | DIFF | chrXIII | 18,778,264 | 18,778,264 | 1 | 1 | 0 | 0 | 1 |
| 15.1 | DIFF | chrXV | 13,517,612 | 14,034,673 | 517,062 | 9 | 0 | 1 | 6 |

The last three columns indicate the number of SNPs that show parallel differentiation between lake and both replicate stream sites (“Parallel”), the number of SNPs with significant differentiation among ecotypes breeding in sympatry (“Sympat”, L1 vs. S1) and in parapatry (“Parapat.”, L2 vs. S2). Note that the genomic coordinates are based on the re-assembly reference genome [77].
