# Supplementary material for: Genomics of Rapid Incipient Speciation in Sympatric Threespine Stickleback
Source: PLoS Genet. 2016 Feb 29;12(2):e1005887. doi: 10.1371/journal.pgen.1005887 (PMC4771382; doi:10.1371/journal.pgen.1005887)
Supplement: S2 Table — (DOCX) [file pgen.1005887.s014.docx]

| Test: | islands of differentiation vs. recombination rate | | IPDs vs. recombination rate | | INDs vs. recombination rate | | hierarchical F_ST_ vs. recombination rate | pairwise F_ST_ vs. recombination rate |
| --- | --- | --- | --- | --- | --- | --- | --- | --- |
|  | Kruskal-Wallis-χ2 | Association | Kruskal-Wallis-χ2 | Association | Kruskal-Wallis-χ2 | Association | Spearman’s correlation | Only significant comparisons (+ positive / – negative association) |
| Full dataset | 50.20 | negative *** | 41.87 | negative *** | 9.75 | negative * | 0.000 n.s. | S1a vs L1 (−), S1a vs. L2 (−), S1b vs. L2 (−), S1b vs. S1 (+), S1b vs. S1a (−) |
| Chr I | 2.12 | n.s. | 0.44 | n.s. | 2.32 | n.s. | 0.077 n.s. |  |
| Chr II | 8.65 | positive * | n.a. | − | 8.65 | positive * | 0.152 n.s. | S1b vs. L2 (+), S1b vs. S1 (+), S1b vs. S2 (+), S1b vs. S1a (+) |
| Chr III | 0.48 | n.s. | 0.48 | n.s. | n.a. | − | −0.024 n.s. s | S1 vs. L2 (+) |
| Chr IV | 15.19 | negative ** | 15.19 | negative *** | n.a. | − | −0.011 ** s | S2 vs. S1 (+), S1a vs. L1 (+), S1b vs. L1 (+) |
| Chr V | 3.37 | n.s. | n.a. | − | 3.37 | n.s. | −0.150 n.s. s | L2 vs. L1 (−), S1 vs. L1 (+) |
| Chr VI | n.a. | − | n.a. | − | n.a. | − | 0.053 n.s. | S2 vs. S1 (+), S1a vs. L2 (−) |
| Chr VII | 210.65 | negative *** | 202.74 | negative *** | 10.07 | negative * | −0.255 *** s | S1 vs. L2 (−), S2 vs. L2 (−), S1a vs. S2 (−), S1b vs. S1 (−) |
| Chr VIII | n.a. | − | n.a. | − | n.a. | − | −0.032 n.s.s | S1a vs. L1 (−), S1a vs. L2 (−), S1b vs. L2 (−), S1b vs. S1a (−) |
| Chr IX | 28.01 | negative *** | 12.63 | negative ** | 16.00 | negative *** | −0.188 ***s | S2 vs. L1 (−), S1a vs. S1 (−), S1a vs. S2 (−), S1b vs. S1 (+) |
| Chr X | n.a. | − | n.a. | − | n.a. | − | −0.041 n.s.s |  |
| Chr XI | 1.21 | n.s. | n.a. | − | 1.21 | n.s. | −0.164 n.s.s | S1b vs. S1 (−) |
| Chr XII | 3.55 | n.s. | 2.50 | n.s. | 1.19 | n.s. | 0.137 n.s. | S1 vs. L1 (+), S1 vs. L2 (+), S2 vs. S1 (+) |
| Chr XIII | 7.76 | n.s. | 5.25 | n.s. | 2.93 | n.s. | 0.199 n.s. | S2 vs. L2 (+), S2 vs. S1 (+), S1a vs. L2 (−) |
| Chr XIV | n.a. | - | n.a. | − | n.a. | − | −0.022 n.s. s | S2 vs. L1 (−) |
| Chr XV | 14.09 | negative ** | n.a. | − | 14.09 | negative ** | −0.097 **s s | S2 vs. L2 (−), S2 vs. S1 (−), S1b vs. S2 (−) |
| Chr XVI | n.a. | − | n.a. | − | n.a. | − | 0.073 n.s.s |  |
| Chr XVII | n.a. | − | n.a. | − | n.a. | − | −0.034 n.s. s | S1b vs. S1 (−) |
| Chr XVIII | n.a. | − | n.a. | − | n.a. | − | −0.033 n.s. s | S1a vs. L1 (+), S1b vs. L1 (−) |
| Chr XX | n.a. | − | n.a. | − | n.a. | − | 0.117 n.s.s | S2 vs. L1 (+), S2 vs. S1 (+), S1b vs. S1 (+) |
| Chr XXI | n.a. | − | n.a. | − | n.a. | − | −0.086 n.s. s |  |

Bonferroni-corrected significance levels are indicated with asterisk: * α < 0.05, ** α < 0.01, *** α < 0.001, n.s. = not significant, n.a. = not available (impossible test due to the lack of island of differentiation, IPDs or INDs on the respective chromosome).
