# Supplementary material for: Genomics of Rapid Incipient Speciation in Sympatric Threespine Stickleback
Source: PLoS Genet. 2016 Feb 29;12(2):e1005887. doi: 10.1371/journal.pgen.1005887 (PMC4771382; doi:10.1371/journal.pgen.1005887)
Supplement: S3 Table — (DOCX) [file pgen.1005887.s015.docx]

| Type | Chrom. | Start | End | Confidence interval | Trait category | Trait | PVE | Overlapping island ID | Ref. |
| --- | --- | --- | --- | --- | --- | --- | --- | --- | --- |
| QTL | chrI | 675889 | 27833078 | 1.5-LOD region | Feeding morphology | Opercle width | 4.43 | 1.3 | [96] |
| QTL | chrI | 2220911 | 26363078 | 1.5-LOD region | Feeding morphology | Even row raker number | 3.17 | 1.3 | [96] |
| QTL | chrI | 3310077 | 27266031 | 1.5-LOD region | Feeding morphology | Number of short gill rakers | 3.1 | 1.3 | [93] |
| QTL | chrI | 7545826 | 27266031 | 1.5-LOD region | Predator defense | Posterodorsal corner ectocoracoid (x) | 2.61 | 1.3 | [93] |
| QTL | chrI | 11963492 | 28438531 | 1.5-LOD region | Camouflage | Ventral melanophores | 8.9 | 1.3 | [88] |
| QTL | chrI | 24933125 | 28635278 | 1.5-LOD region | Feeding morphology | Dorsal toothplate 2 length | 3.74 | 1.3 | [96] |
| QTL | chrIII | 4622006 | 16391681 | 1.5-LOD region | Feeding morphology | Premaxilla length | 3.69 | 3.1 | [96] |
| QTL | chrIV | 339710 | 29763654 | 1.5-LOD region | Feeding morphology | Posteroventral extent maxilla (y) | 2.75 | 4.1 | [93] |
| QTL | chrIV | 339710 | 31350187 | 1.5-LOD region | Schooling behavior | Ventral margin orbit (y) | 4.76 | 4.1 | [93] |
| QTL | chrIV | 1283448 | 28557932 | 1.5-LOD region | Feeding morphology | In-lever 1 of articular length | 5.66 | 4.1 | [96] |
| QTL | chrIV | 1926094 | 24823118 | 1.5-LOD region | Feeding morphology | Row 2 epi raker number | 5.8 | 4.1 | [96] |
| QTL | chrIV | 1926094 | 28557932 | 1.5-LOD region | Feeding morphology | Supraoccipital crest length | 5.58 | 4.1 | [96] |
| QTL | chrIV | 1942641 | 31350187 | 1.5-LOD region | Feeding morphology | Posterior extent supraoccipital along dorsal silhouette (y) | 3.56 | 4.1 | [93] |
| QTL | chrIV | 1942641 | 32592491 | 1.5-LOD region | Predator defense | First dorsal spine development | 2.55 | 4.1 | [93] |
| QTL | chrIV | 1942641 | 29034665 | 1.5-LOD region | Predator defense | Posteroventral corner ectocoracoid (y) | 3.62 | 4.1 | [93] |
| QTL | chrIV | 2045971 | 30568387 | 1.5-LOD region | Schooling behavior | Anterior margin orbit (x) | 4.8 | 4.1 | [93] |
| QTL | chrIV | 3004386 | 24823118 | 1.5-LOD region | Feeding morphology | Ceratobranichial 4 | 5.08 | 4.1 | [99] |
| QTL | chrIV | 3004386 | 28557932 | 1.5-LOD region | Swimming performance | Third predorsal pterygiophore position | 9.11 | 4.1 | [96] |
| QTL | chrIV | 4034002 | 30568387 | 1.5-LOD region | Predator defense | Posterodorsal corner ectocoracoid (y) | 3.83 | 4.1 | [93] |
| QTL | chrIV | 4455356 | 28557932 | 1.5-LOD region | Feeding morphology | Row 4 joint raker number | 8.61 | 4.1 | [96] |
| QTL | chrIV | 4455356 | 28557932 | 1.5-LOD region | Feeding morphology | Joint raker number | 8.91 | 4.1 | [96] |
| QTL | chrIV | 4455356 | 21320930 | 1.5-LOD region | Feeding morphology | Premaxilla height | 3.97 | 4.1 | [96] |
| QTL | chrIV | 4455356 | 24823118 | 1.5-LOD region | Feeding morphology | Ceratobranichial 4 | 9.03 | 4.1 | [99] |
| QTL | chrIV | 4455356 | 20798777 | 1.5-LOD region | Swimming performance | Dorsal fin ray number | 6.05 | 4.1 | [96] |
| QTL | chrIV | 4859295 | 29823254 | 1.5-LOD region | Feeding morphology | Middle gill raker length | 7.6 | 4.1 | [102] |
| QTL | chrIV | 5165268 | 29763654 | 1.5-LOD region | Feeding morphology | Anterior epaxial muscle width (residual) | 8.88 | 4.1 | [93] |
| QTL | chrIV | 6107609 | 28557932 | 1.5-LOD region | Feeding morphology | Row 4 cerato raker number | 5.21 | 4.1 | [96] |
| QTL | chrIV | 6107609 | 28557932 | 1.5-LOD region | Feeding morphology | Row 3 hypo raker number | 5.45 | 4.1 | [96] |
| QTL | chrIV | 6107609 | 28557932 | 1.5-LOD region | Feeding morphology | Row 4 raker number | 7.56 | 4.1 | [96] |
| QTL | chrIV | 6107609 | 28557932 | 1.5-LOD region | Feeding morphology | Branchial arch 4 raker number | 5.4 | 4.1 | [96] |
| QTL | chrIV | 6107609 | 20798777 | 1.5-LOD region | Feeding morphology | Dorsal toothplate 1 tooth number | 14.88 | 4.1 | [96] |
| QTL | chrIV | 6107609 | 27175470 | 1.5-LOD region | Feeding morphology | Ventral toothplate tooth number | 7.35 | 4.1 | [96] |
| QTL | chrIV | 6107609 | 20798777 | 1.5-LOD region | Feeding morphology | Dorsal toothplate 2 length | 6.11 | 4.1 | [96] |
| QTL | chrIV | 6107609 | 28557932 | 1.5-LOD region | Feeding morphology | Epibranchial 1 length | 11.8 | 4.1 | [96] |
| QTL | chrIV | 6107609 | 28557932 | 1.5-LOD region | Feeding morphology | Ceratobranchial 1 length | 13.58 | 4.1 | [96] |
| QTL | chrIV | 6107609 | 28557932 | 1.5-LOD region | Feeding morphology | Ceratobranchial 2 length | 14.36 | 4.1 | [96] |
| QTL | chrIV | 6107609 | 20798777 | 1.5-LOD region | Feeding morphology | Ceratobranchial 3 length | 20.71 | 4.1 | [96] |
| QTL | chrIV | 6107609 | 28557932 | 1.5-LOD region | Feeding morphology | Premaxilla length | 4.6 | 4.1 | [96] |
| QTL | chrIV | 6107609 | 21890975 | 1.5-LOD region | Predator defense | Dorsal spine 3 length | 17.73 | 4.1 | [96] |
| QTL | chrIV | 6107609 | 28557932 | 1.5-LOD region | Swimming performance | Anal fin ray number | 7.17 | 4.1 | [96] |
| QTL | chrIV | 6107609 | 28557932 | 1.5-LOD region | Swimming performance | Total postdorsal pterygiophore number | 7.7 | 4.1 | [96] |
| QTL | chrIV | 6107609 | 28557932 | 1.5-LOD region | Swimming performance | Total dorsal pterygiophore number | 7.43 | 4.1 | [96] |
| QTL | chrIV | 8579158 | 29763654 | 95% confidence interval | Mate choice | Lateral line: neuromast number supratemporal | 11.8 | 4.1 | [91] |
| QTL | chrIV | 10585867 | 29457182 | associated marker ± 1 Mb | Predator defense | Ectocorocoid length | 13.57 | 4.1 | [90] |
| QTL | chrIV | 10585867 | 29457182 | associated marker ± 1 Mb | Predator defense | Pelvic girdle length | 28.87 | 4.1 | [90] |
| QTL | chrIV | 10585867 | 29457182 | associated marker ± 1 Mb | Predator defense | Pelvic spine length | 40.56 | 4.1 | [90] |
| QTL | chrIV | 10585867 | 29457182 | associated marker ± 1 Mb | Predator defense | Anterior insertion of first dorsal spine (x) | 14.68 | 4.1 | [90] |
| QTL | chrIV | 10585867 | 29457182 | associated marker ± 1 Mb | Predator defense | Anterior extent of ectocorocoid (x) | 11.16 | 4.1 | [90] |
| QTL | chrIV | 10585867 | 29457182 | associated marker ± 1 Mb | Predator defense | Dorsal extent of the ascending branch of the pelvis (y) | 18.35 | 4.1 | [90] |
| QTL | chrIV | 10585867 | 29457182 | associated marker ± 1 Mb | Swimming performance | Pectoral fin length | 15.41 | 4.1 | [90] |
| QTL | chrIV | 11585867 | 23504625 | 95% confidence interval | Predator defense | Number of plates | 74.4 | 4.1 | [95] |
| QTL | chrIV | 11585867 | 26512456 | 1.5-LOD region | Predator defense | Dorsal spine 1 length | 31.58 | 4.1 | [96] |
| QTL | chrIV | 11742882 | 28557932 | 1.5-LOD region | Feeding morphology | Row 6 raker number | 5.13 | 4.1 | [96] |
| QTL | chrIV | 11742882 | 28557932 | 1.5-LOD region | Predator defense | Anal spine length | 21 | 4.1 | [96] |
| QTL | chrIV | 12809576 | 28557932 | 1.5-LOD region | Feeding morphology | Row 2 joint raker number | 7.87 | 4.1 | [96] |
| QTL | chrIV | 12809576 | 28557932 | 1.5-LOD region | Feeding morphology | Row 2 cerato raker number | 14.22 | 4.1 | [96] |
| QTL | chrIV | 12809576 | 26512456 | 1.5-LOD region | Feeding morphology | Row 6 cerato raker number | 5.82 | 4.1 | [96] |
| QTL | chrIV | 12809576 | 28557932 | 1.5-LOD region | Feeding morphology | Hypo raker number | 6.23 | 4.1 | [96] |
| QTL | chrIV | 12809576 | 28557932 | 1.5-LOD region | Feeding morphology | Row 1 raker number | 4.05 | 4.1 | [96] |
| QTL | chrIV | 12809576 | 26512456 | 1.5-LOD region | Feeding morphology | Row 3 raker number | 13.6 | 4.1 | [96] |
| QTL | chrIV | 12809576 | 27175470 | 1.5-LOD region | Feeding morphology | Branchial arch 2 raker number | 13.34 | 4.1 | [96] |
| QTL | chrIV | 12809576 | 21320930 | 1.5-LOD region | Feeding morphology | Lateral raker spacing | 22.63 | 4.1 | [96] |
| QTL | chrIV | 12809576 | 21890975 | 1.5-LOD region | Feeding morphology | Middle raker spacing | 22.3 | 4.1 | [96] |
| QTL | chrIV | 12809576 | 27175470 | 1.5-LOD region | Feeding morphology | Medial raker spacing | 20.49 | 4.1 | [96] |
| QTL | chrIV | 14232047 | 29457182 | associated marker ± 1 Mb | Predator defense | Pelvic girdle length | 8.24 | 4.1 | [90] |
| QTL | chrIV | 14232047 | 29457182 | associated marker ± 1 Mb | Predator defense | Pelvic spine length | 11.91 | 4.1 | [90] |
| QTL | chrIV | 14232047 | 29457182 | associated marker ± 1 Mb | Predator defense | Anterior insertion of first dorsal spine (x) | 10.63 | 4.1 | [90] |
| QTL | chrIV | 14232047 | 29457182 | associated marker ± 1 Mb | Predator defense | Anterior insertion of first dorsal spine (y) | 9.02 | 4.1 | [90] |
| QTL | chrIV | 14933780 | 26512456 | 1.5-LOD region | Feeding morphology | Row 3 cerato raker number | 11.39 | 4.1 | [96] |
| QTL | chrIV | 14933780 | 28557932 | 1.5-LOD region | Feeding morphology | Cerato raker number | 11.94 | 4.1 | [96] |
| QTL | chrIV | 14933780 | 28557932 | 1.5-LOD region | Feeding morphology | Branchial arch 3 raker number | 5.63 | 4.1 | [96] |
| QTL | chrIV | 14933780 | 28557932 | 1.5-LOD region | Feeding morphology | Odd row raker number | 9.17 | 4.1 | [96] |
| QTL | chrIV | 14933780 | 28557932 | 1.5-LOD region | Swimming performance | Total postanal pterygiophore number | 11.05 | 4.1 | [96] |
| QTL | chrIV | 15232047 | 28557932 | 1.5-LOD region | Feeding morphology | Row 5 cerato raker number | 7.96 | 4.1 | [96] |
| QTL | chrIV | 15232047 | 28557932 | 1.5-LOD region | Feeding morphology | Row 2 raker number | 17.09 | 4.1 | [96] |
| QTL | chrIV | 15232047 | 28557932 | 1.5-LOD region | Swimming performance | Last postdorsal pterygiophore position | 5.93 | 4.1 | [96] |
| QTL | chrIV | 15232047 | 28557932 | 1.5-LOD region | Swimming performance | Last postanal pterygiophore position | 8.55 | 4.1 | [96] |
| QTL | chrIV | 15366117 | 30500157 | 1.5-LOD region | Feeding morphology | Gill raker number | 12.5 | 4.1 | [98] |
| QTL | chrIV | 15366117 | 30500157 | 1.5-LOD region | Feeding morphology | Gill raker number | 20.7 | 4.1 | [98] |
| QTL | chrIV | 15625777 | 28557932 | 1.5-LOD region | Feeding morphology | Even row raker number | 12.08 | 4.1 | [96] |
| QTL | chrIV | 15625777 | 28557932 | 1.5-LOD region | Feeding morphology | All raker number | 10.22 | 4.1 | [96] |
| QTL | chrIV | 16331734 | 28557932 | 1.5-LOD region | Feeding morphology | Row 1 cerato raker number | 11.69 | 4.1 | [96] |
| QTL | chrIV | 16331734 | 28557932 | 1.5-LOD region | Feeding morphology | Branchial arch 1 raker number | 8.89 | 4.1 | [96] |
| QTL | chrIV | 19798777 | 21799032 | associated marker ± 1 Mb | Feeding morphology | Tooth number | 6 | 4.1 | [97] |
| QTL | chrVII | 88297 | 5222584 | 1.5-LOD region | Feeding morphology | Hypo raker number | 5.7 | 7.2 | [96] |
| QTL | chrVII | 338787 | 26856360 | 1.5-LOD region | Feeding morphology | Row 3 raker number | 3.69 | 7.2,7.4-7.14 | [96] |
| QTL | chrVII | 338787 | 28197556 | 1.5-LOD region | Feeding morphology | Branchial arch 3 raker number | 3.79 | 7.2,7.4-7.14 | [96] |
| QTL | chrVII | 338787 | 5222584 | 1.5-LOD region | Feeding morphology | Dorsal toothplate 1 tooth number | 5.08 | 7.2 | [96] |
| QTL | chrVII | 1038649 | 29685623 | 95% confidence interval , to chromosome end | Predator defense | Second spine length | 7.26 | 7.2,7.4-7.14 | [95] |
| QTL | chrVII | 1038649 | 29685623 | 95% confidence interval , to chromosome end | Predator defense | Pelvic spine length | 6.32 | 7.2,7.4-7.14 | [95] |
| QTL | chrVII | 1038766 | 7478687 | 1.5-LOD region | Feeding morphology | Odd row raker number | 4.54 | 7.2,7.4-7.6 | [96] |
| QTL | chrVII | 1038766 | 11075956 | 1.5-LOD region | Feeding morphology | All raker number | 3.98 | 7.2,7.4-7.12 | [96] |
| QTL | chrVII | 1038766 | 28197556 | 1.5-LOD region | Feeding morphology | Dorsal toothplate 2 tooth number | 4.43 | 7.2,7.4-7.14 | [96] |
| QTL | chrVII | 1038766 | 25978258 | 1.5-LOD region | Feeding morphology | Frontal width | 5.38 | 7.2,7.4-7.14 | [96] |
| QTL | chrVII | 1440718 | 6222584 | associated marker ± 1 Mb | Feeding morphology | Snout length | 6.87 | 7.2 | [90] |
| QTL | chrVII | 1440718 | 6222584 | associated marker ± 1 Mb | Swimming performance | Posterior insertion of anal fin (y) | 8.9 | 7.2 | [90] |
| QTL | chrVII | 1481322 | 26941261 | 1.5-LOD region | Feeding morphology | Dorsalmost extent preopercle (x) | 3.02 | 7.2,7.4-7.14 | [93] |
| QTL | chrVII | 2300505 | 5222127 | 95% confidence interval | Predator defense | First spine length | 9.05 | 7.2 | [95] |
| QTL | chrVII | 2440718 | 26856360 | 1.5-LOD region | Feeding morphology | Row 1 cerato raker number | 6.99 | 7.2,7.4-7.14 | [96] |
| QTL | chrVII | 2440718 | 28197556 | 1.5-LOD region | Feeding morphology | Row 1 raker number | 5 | 7.2,7.4-7.14 | [96] |
| QTL | chrVII | 2440718 | 7478687 | 1.5-LOD region | Feeding morphology | Branchial arch 1 raker number | 5.39 | 7.2,7.4-7.6 | [96] |
| QTL | chrVII | 2440718 | 26856360 | 1.5-LOD region | Feeding morphology | Branchial arch 2 raker number | 4.2 | 7.2,7.4-7.14 | [96] |
| QTL | chrVII | 2440718 | 25978258 | 1.5-LOD region | Feeding morphology | Dorsal toothplate 1 length | 11.77 | 7.2,7.4-7.14 | [96] |
| QTL | chrVII | 2440718 | 26856360 | 1.5-LOD region | Feeding morphology | Ventral toothplate width | 4.76 | 7.2,7.4-7.14 | [96] |
| QTL | chrVII | 2440718 | 26856360 | 1.5-LOD region | Feeding morphology | Articular height | 5.79 | 7.2,7.4-7.14 | [96] |
| QTL | chrVII | 2440718 | 26856360 | 1.5-LOD region | Feeding morphology | In-lever 1 of articular length | 5.02 | 7.2,7.4-7.14 | [96] |
| QTL | chrVII | 2440718 | 28197556 | 1.5-LOD region | Predator defense | Dorsal spine 1 length | 4.5 | 7.2,7.4-7.14 | [96] |
| QTL | chrVII | 2559099 | 26941261 | 1.5-LOD region | Feeding morphology | Dorsal edge opercle–hyomandibular boundary (x) | 4.22 | 7.2,7.4-7.14 | [93] |
| QTL | chrVII | 2559099 | 27658403 | 1.5-LOD region | Feeding morphology | Number of short gill rakers | 5.15 | 7.2,7.4-7.14 | [93] |
| QTL | chrVII | 4222423 | 6222584 | associated marker ± 1 Mb | Predator defense | Adjusted plate height | 10.3 | 7.2 | [79] |
| QTL | chrVII | 4222423 | 6222584 | associated marker ± 1 Mb | Predator defense | Anterior extent of ectocorocoid (y) | 9.4 | 7.2 | [90] |
| QTL | chrVII | 4367333 | 23218289 | 1.5-LOD region | Predator defense | Plate number (Eda heterozygotes) | 13.3 | 7.2,7.4-7.14 | [102] |
| QTL | chrVII | 5222423 | 26856360 | 1.5-LOD region | Feeding morphology | Epibranchial 1 length | 4.23 | 7.4-7.14 | [96] |
| QTL | chrVII | 5936068 | 25965786 | 1.5-LOD region | Feeding morphology | Number of long gill rakers | 11.7 | 7.4-7.14 | [93] |
| QTL | chrVII | 6478502 | 8478687 | associated marker ± 1 Mb | Predator defense | Plate number in AA F2s | 3.1 | 7.4-7.9 | [79] |
| QTL | chrVII | 6478502 | 8478687 | associated marker ± 1 Mb | Predator defense | Plate number in Aa F2s | 11.3 | 7.4-7.9 | [79] |
| QTL | chrVII | 6478502 | 8478687 | associated marker ± 1 Mb | Predator defense | Plate number in aa F2s | 11.4 | 7.4-7.9 | [79] |
| QTL | chrVII | 6478502 | 8478687 | associated marker ± 1 Mb | Predator defense | Plate number in all F2s | 3.7 | 7.4-7.9 | [79] |
| QTL | chrVII | 6478502 | 8478687 | associated marker ± 1 Mb | Predator defense | Adjusted plate width | 11.1 | 7.4-7.9 | [79] |
| QTL | chrVII | 6478502 | 8478687 | associated marker ± 1 Mb | Swimming performance | Shape males | 4.75 | 7.4-7.9 | [89] |
| QTL | chrVII | 6478502 | 8478687 | associated marker ± 1 Mb | Visual morphology | Color males | 20 | 7.4-7.9 | [89] |
| QTL | chrVII | 7478502 | 28197556 | 1.5-LOD region | Feeding morphology | Dorsal toothplate 2 width | 9.2 | 7.6-7.14 | [96] |
| QTL | chrVII | 7478502 | 26856360 | 1.5-LOD region | Feeding morphology | Dentary length | 4.21 | 7.6-7.14 | [96] |
| QTL | chrVII | 7478502 | 26856360 | 1.5-LOD region | Feeding morphology | In-lever 2 of articular length | 11.93 | 7.6-7.14 | [96] |
| QTL | chrVII | 7478502 | 26856360 | 1.5-LOD region | Feeding morphology | Opercle length | 11.12 | 7.6-7.14 | [96] |
| QTL | chrVII | 11075487 | 26856360 | 1.5-LOD region | Feeding morphology | Dorsal toothplate 2 length | 13.73 | 7.12-7.14 | [96] |
| QTL | chrVII | 14411863 | 16412432 | associated marker ± 1 Mb | Feeding morphology | Ventral extent of operculum (x) | 7.2 | 7.14 | [86] |
| QTL | chrVII | 14411863 | 16412432 | associated marker ± 1 Mb | Feeding morphology | Posteriodorsal extent of operculum (x) | 3.2 | 7.14 | [86] |
| QTL | chrVII | 14411863 | 16412432 | associated marker ± 1 Mb | Feeding morphology | Posterior extent of maxilla (x) | 5.3 | 7.14 | [86] |
| QTL | chrVII | 14411863 | 16412432 | associated marker ± 1 Mb | Feeding morphology | Anterior extent of maxilla (x) | 3.6 | 7.14 | [86] |
| QTL | chrVII | 14411863 | 16412432 | associated marker ± 1 Mb | Feeding morphology | Posterior extent of maxilla (y) | 5.6 | 7.14 | [86] |
| QTL | chrVII | 14411863 | 16412432 | associated marker ± 1 Mb | Feeding morphology | Lachrymal at nasal capsule (y) | 6.8 | 7.14 | [86] |
| QTL | chrVII | 14411863 | 16412432 | associated marker ± 1 Mb | Predator defense | Posterior extent of ectocorocoid (x) | 9.7 | 7.14 | [86] |
| QTL | chrVII | 14411863 | 16412432 | associated marker ± 1 Mb | Predator defense | Dorsal extent of ectocorocoid (x) | 8.9 | 7.14 | [86] |
| QTL | chrVII | 14411863 | 16412432 | associated marker ± 1 Mb | Predator defense | Anterior insertion of first dorsal spine (x) | 10.1 | 7.14 | [86] |
| QTL | chrVII | 14411863 | 16412432 | associated marker ± 1 Mb | Predator defense | Anterior extent of ectocorocoid (y) | 7.5 | 7.14 | [86] |
| QTL | chrVII | 14411863 | 16412432 | associated marker ± 1 Mb | Swimming performance | Anterior insertion of anal fin (x) | 3.8 | 7.14 | [86] |
| QTL | chrVII | 14411863 | 16412432 | associated marker ± 1 Mb | Swimming performance | Shape males | 4.64 | 7.14 | [89] |
| QTL | chrVII | 14411863 | 16412432 | associated marker ± 1 Mb | Visual morphology | Color males | 20 | 7.14 | [89] |
| QTL | chrIX | 2131739 | 16873242 | 1.5-LOD region | Feeding morphology | Dorsal toothplate 1 length | 4.74 | 9.4 | [96] |
| QTL | chrIX | 2131739 | 15365128 | 1.5-LOD region | Feeding morphology | Articular length | 5.65 | 9.4 | [96] |
| QTL | chrIX | 2131739 | 11977493 | 1.5-LOD region | Predator defense | Dorsal spine 1 length | 4.35 | 9.4 | [96] |
| QTL | chrIX | 6007326 | 15225711 | 1.5-LOD region | Feeding morphology | Ventral tooth plate intertooth spacing | 11.3 | 9.4 | [100] |
| QTL | chrIX | 6727850 | 18826248 | 1.5-LOD region | Feeding morphology | Upper jaw protrusion length (residual) | 8.82 | 9.4 | [93] |
| QTL | chrIX | 8075888 | 12668311 | 1.5-LOD region | Predator defense | Dorsal spine 2 length | 7 | 9.4 | [96] |
| QTL | chrIX | 8351078 | 18826248 | 1.5-LOD region | Feeding morphology | Buccal cavity length (size-corrected residual) | 3.05 | 9.4 | [93] |
| QTL | chrIX | 8351078 | 18942598 | 1.5-LOD region | Feeding morphology | Neurocranium outlever length (residual) | 2.97 | 9.4 | [93] |
| QTL | chrXII | 1 | 7703283 | associated marker ± 1 Mb | Feeding morphology | Dorsal extent of preopercular (y) | 8.95 | 12.3,12.5 | [90] |
| QTL | chrXII | 1 | 7703283 | associated marker ± 1 Mb | Predator defense | Posterior extent of ectocorocoid (y) | 10.97 | 12.3,12.5 | [90] |
| QTL | chrXII | 1 | 7703283 | associated marker ± 1 Mb | Swimming performance | Body depth | 9.04 | 12.3,12.5 | [90] |
| QTL | chrXII | 548804 | 15583756 | 1.5-LOD region | Feeding morphology | Dorsal edge opercle–hyomandibular boundary (y) | 3.56 | 12.3,12.5 | [93] |
| QTL | chrXII | 2224031 | 19065992 | 1.5-LOD region | Feeding morphology | Dorsal toothplate 1 width | 5.24 | 12.3,12.5 | [96] |
| QTL | chrXII | 2224031 | 13826422 | 1.5-LOD region | Feeding morphology | Opercle neck width | 8.4 | 12.3,12.5 | [96] |
| QTL | chrXII | 2526062 | 10447269 | 1.5-LOD region | Feeding morphology | Ceratobranchial 4 length | 3.46 | 12.3,12.5 | [96] |
| QTL | chrXII | 2526062 | 19065992 | 1.5-LOD region | Feeding morphology | Dentary height | 6.94 | 12.3,12.5 | [96] |
| QTL | chrXII | 3050245 | 12713072 | 1.5-LOD region | Feeding morphology | Anterior epaxial muscle width (residual) | 6.32 | 12.3,12.5 | [93] |
| QTL | chrXII | 3050245 | 15583756 | 1.5-LOD region | Feeding morphology | Anteroventral corner opercle (x) | 3.99 | 12.3,12.5 | [93] |
| QTL | chrXII | 3247640 | 5247781 | associated marker ± 1 Mb | Predator defense | Anterior insertion of second dorsal spine (x) | 11.07 | 12.3 | [90] |
| QTL | chrXII | 4247775 | 13826422 | 1.5-LOD region | Feeding morphology | Premaxilla length | 3.7 | 12.3,12.5 | [96] |
| QTL | chrXII | 4272748 | 6272748 | associated marker ± 1 Mb | Mate choice | Lateral line: neuromast number supratemporal | 11.7 | 12.3,12.5 | [91] |
| QTL | chrXII | 4280118 | 6280118 | associated marker ± 1 Mb | Mate choice | Lateral line: neuromast number supratemporal | 11.7 | 12.3,12.5 | [91] |
| QTL | chrXII | 5703283 | 7703880 | associated marker ± 1 Mb | Feeding morphology | Ventral extent of operculum (x) | 4.8 | 12.5 | [86] |
| QTL | chrXII | 5703283 | 7703880 | associated marker ± 1 Mb | Feeding morphology | Posterioventral extent of preopercular (x) | 11.6 | 12.5 | [86] |
| QTL | chrXII | 5703283 | 7703880 | associated marker ± 1 Mb | Predator defense | Insertion point of pelvic spine into the pelvic girdle (x) | 5.2 | 12.5 | [86] |
| QTL | chrXII | 5703283 | 7703880 | associated marker ± 1 Mb | Predator defense | Dorsal extent of the ascending branch of the pelvis (x) | 10.3 | 12.5 | [86] |
| QTL | chrXII | 5703283 | 7703880 | associated marker ± 1 Mb | Predator defense | Anterior insertion of third dorsal spine (x) | 7.7 | 12.5 | [86] |
| QTL | chrXII | 5703283 | 7703880 | associated marker ± 1 Mb | Predator defense | Insertion point of pelvic spine into the pelvic girdle (y) | 7.7 | 12.5 | [86] |
| QTL | chrXII | 5703283 | 7703880 | associated marker ± 1 Mb | Predator defense | Dorsal extent of the ascending branch of the pelvis (y) | 4.1 | 12.5 | [86] |
| QTL | chrXII | 5703283 | 7703880 | associated marker ± 1 Mb | Predator defense | Posterior extent of ectocorocoid (y) | 10 | 12.5 | [86] |
| QTL | chrXII | 5703283 | 7703880 | associated marker ± 1 Mb | Predator defense | Dorsal extent of ectocorocoid (y) | 12.2 | 12.5 | [86] |
| QTL | chrXII | 5703283 | 7703880 | associated marker ± 1 Mb | Predator defense | Anterior insertion of first dorsal spine (y) | 10 | 12.5 | [86] |
| QTL | chrXII | 5703283 | 7703880 | associated marker ± 1 Mb | Predator defense | Anterior insertion of second dorsal spine (y) | 12.4 | 12.5 | [86] |
| QTL | chrXII | 5703283 | 7703880 | associated marker ± 1 Mb | Predator defense | Anterior insertion of third dorsal spine (y) | 13.5 | 12.5 | [86] |
| QTL | chrXII | 5703283 | 7703880 | associated marker ± 1 Mb | Predator defense | Posterior extent of ectocorocoid (x) | 7.72 | 12.5 | [90] |
| QTL | chrXII | 5703880 | 14826422 | associated marker ± 1 Mb | Feeding morphology | Anteriodorsal extent of operculum (x) | 7.57 | 12.5 | [90] |
| QTL | chrXII | 5703880 | 14826422 | associated marker ± 1 Mb | Feeding morphology | Dorsal extent of preopercular (x) | 9.6 | 12.5 | [90] |
| QTL | chrXII | 5703880 | 14826422 | associated marker ± 1 Mb | Predator defense | Anterior insertion of third dorsal spine (x) | 9.15 | 12.5 | [90] |
| QTL | chrXII | 5703880 | 14826422 | associated marker ± 1 Mb | Predator defense | Dorsal extent of the ascending branch of the pelvis (x) | 9.66 | 12.5 | [90] |
| QTL | chrXII | 5703880 | 14826422 | associated marker ± 1 Mb | Swimming performance | Posterior insertion of anal fin (y) | 10.05 | 12.5 | [90] |
| QTL | chrXII | 5800080 | 13775690 | 95% confidence interval | Swimming performance | Base of the first anal ray on ventral midline (y) | 9.1 | 12.5 | [95] |
| QTL | chrXII | 5800080 | 8217033 | 95% confidence interval | Swimming performance | Insertion of dorsal fin membrane on dorsal midline (x) | 9.34 | 12.5 | [95] |
| QTL | chrXIII | 2822352 | 18821497 | 1.5-LOD region | Feeding morphology | Anteroventral corner opercle (x) | 2.12 | 13.1 | [93] |
| QTL | chrXIII | 2822352 | 18821497 | 1.5-LOD region | Feeding morphology | Suture between nasal and frontal bones along dorsal silhouette (x) | 2.96 | 13.1 | [93] |
| QTL | chrXIII | 5298608 | 18957152 | 1.5-LOD region | Predator defense | Dorsal spine 3 length | 4.65 | 13.1 | [96] |
| QTL | chrXIII | 6754994 | 18957152 | 1.5-LOD region | Feeding morphology | Middle raker spacing | 3.64 | 13.1 | [96] |
| QTL | chrXIII | 8142821 | 18957152 | 1.5-LOD region | Feeding morphology | Dorsal toothplate 1 width | 5.19 | 13.1 | [96] |
| QTL | chrXIII | 14905199 | 19957335 | associated marker ± 1 Mb | Predator defense | Ectocorocoid length | 10.5 | 13.1 | [90] |
| QTL | chrXIII | 15836768 | 19957335 | associated marker ± 1 Mb | Predator defense | Posterior extent of ectocorocoid (y) | 10.67 | 13.1 | [90] |
| QTL | chrXIII | 15836768 | 19957335 | associated marker ± 1 Mb | Swimming performance | Posterior insertion of dorsal fin (x) | 8.8 | 13.1 | [90] |
| QTL | chrXIII | 16896505 | 18896505 | associated marker ± 1 Mb | Mate choice | Lateral line: neuromast number main trunk line anterior | 13.9 | 13.1 | [91] |
| Outlier | chrI | 23932975 | 25933125 | outlier microsat ± 1 Mb | Outlier | Marine-freshwater, freshwater-freshwater | 0 | 1.3 | [125] |
| Outlier | chrI | 24482352 | 26512879 | outlier microsat ± 1 Mb | Outlier | Marine-freshwater | 0 | 1.3 | [127] |
| Outlier | chrI | 24911041 | 26029570 | gene content in outlier region ± 100 kb | Outlier | Marine-freshwater | 0 | 1.3 | [24] |
| Outlier | chrI | 25221825 | 25377711 | exact region | Outlier | Lake-stream | 0 | 1.3 | [24] |
| Outlier | chrI | 25252411 | 25724532 | exact region | Outlier | Marine-freshwater | 0 | 1.3 | [45] |
| Outlier | chrI | 25347228 | 25365153 | exact region | Outlier | Marine-freshwater | 0 | 1.3 | [26] |
| Outlier | chrI | 25357530 | 25363530 | exact region | Outlier | Marine-freshwater | 0 | 1.3 | [26] |
| Outlier | chrI | 25423238 | 25623238 | outlier SNP ± 100 kb | Outlier | Marine-freshwater | 0 | 1.3 | [25] |
| Outlier | chrI | 25448552 | 25648552 | outlier SNP ± 100 kb | Outlier | Marine-freshwater | 0 | 1.3 | [25] |
| Outlier | chrI | 25560530 | 25686530 | exact region | Outlier | Marine-freshwater | 0 | 1.3 | [26] |
| Outlier | chrI | 25597030 | 25617530 | exact region | Outlier | Marine-freshwater | 0 | 1.3 | [26] |
| Outlier | chrI | 25606006 | 25806007 | outlier SNP ± 100 kb | Outlier | Lake-stream | 0 | 1.3 | [53] |
| Outlier | chrIII | 7925000 | 9936000 | outlier microsat ± 1 Mb | Outlier | Marine-freshwater | 0 | 3.1 | [127] |
| Outlier | chrVII | 7903928 | 7940621 | exact region | Outlier | Lake-stream | 0 | 7.7 | [24] |
| Outlier | chrVII | 9129793 | 9329794 | outlier SNP ± 100 kb | Outlier | Lake-stream | 0 | 7.9 | [53] |
| Outlier | chrVII | 9501816 | 9701817 | outlier SNP ± 100 kb | Outlier | Lake-stream | 0 | 7.10 | [53] |
| Outlier | chrVII | 10174500 | 10178000 | exact region | Outlier | Marine-freshwater | 0 | 7.11 | [26] |
| Outlier | chrVII | 13698466 | 13898467 | outlier SNP ± 100 kb | Outlier | Lake-stream | 0 | 7.13 | [53] |
| Outlier | chrVII | 14411863 | 16412432 | outlier microsat ± 1 Mb | Outlier | Lake-stream | 0 | 7.14 | [130] |
| Outlier | chrVII | 14411863 | 16412432 | outlier microsat ± 1 Mb | Outlier | Marine-freshwater | 0 | 7.14 | [127] |
| Outlier | chrVII | 14854157 | 15054157 | outlier SNP ± 100 kb | Outlier | Limnetic-benthic | 0 | 7.14 | [25] |
| Outlier | chrVII | 14854157 | 15054157 | outlier SNP ± 100 kb | Outlier | Lake-stream | 0 | 7.14 | [70] |
| Outlier | chrXII | 3025194 | 17340959 | exact region | Outlier | Lake-stream | 0 | 12.3,12.5 | [24] |
| Outlier | chrXII | 4850508 | 6852214 | outlier microsat ± 1 Mb | Outlier | Freshwater-freshwater | 0 | 12.3,12.5 | [125] |
| Outlier | chrXII | 5081291 | 5110290 | exact region | Outlier | Lake-stream | 0 | 12.3 | [24] |
| Outlier | chrXII | 5835018 | 7397188 | gene content in outlier region ± 100 kb | Outlier | Marine-freshwater | 0 | 12.5 | [25] |

PVE: percent variance explained by a single QTL locus. Note that the genomic coordinates are based on the re-assembly reference genome [61].
