# Supplementary material for: Genomics of Rapid Incipient Speciation in Sympatric Threespine Stickleback
Source: PLoS Genet. 2016 Feb 29;12(2):e1005887. doi: 10.1371/journal.pgen.1005887 (PMC4771382; doi:10.1371/journal.pgen.1005887)
Supplement: S4 Table — (DOCX) [file pgen.1005887.s016.docx]

| Island No. | Ensembl Gene ID | Gene Name | Short Name |
| --- | --- | --- | --- |
| 1.3 | ENSGACG00000014275 | *insulin-like growth factor binding protein 5b* | *igfbp5b* |
| 1.3 | ENSGACG00000014280 | *insulin-like growth factor binding protein 2a* | *igfbp2a* |
| 1.3 | ENSGACG00000014289 | *serine/threonine kinase 11 interacting protein* | *stk11ip* |
| 1.3 | ENSGACG00000014294 | *si:dkey-91f15.8* | *si:dkey-91f15.8* |
| 1.3 | ENSGACG00000014296 | *inhibin, alpha* | *inha* |
| 1.3 | ENSGACG00000014299 | *SPEG complex locus* | *speg* |
| 1.3 | ENSGACG00000014313 | *CTD (carboxy-terminal domain, RNA polymerase II, polypeptide A) small phosphatase 1* | *CTDSP1 (2 of 2)* |
| 1.3 | ENSGACG00000014321 | *novel gene* |  |
| 1.3 | ENSGACG00000014323 | *obscurin-like 1a* | *obsl1a* |
| 1.3 | ENSGACG00000014324 | *ATPase, Na+/K+ transporting, alpha 1a polypeptide, tandem duplicate 4* | *atp1a1a.4* |
| 1.3 | ENSGACG00000014345 | *novel gene* |  |
| 1.3 | ENSGACG00000014349 | *coiled-coil domain containing 28A* | *ccdc28a* |
| 1.3 | ENSGACG00000021310 | *novel gene* |  |
| 3.1 | ENSGACG00000015397 | *dynein, axonemal, heavy polypeptide 9 like* | *dnah9l* |
| 3.1 | ENSGACG00000015407 | *novel gene* |  |
| 3.1 | ENSGACG00000015410 | *Rap guanine nucleotide exchange factor (GEF) 4* | *RAPGEF4 (2 of 2)* |
| 3.1 | ENSGACG00000015414 | *si:ch211-269i23.2* | *si:ch211-269i23.2* |
| 3.1 | ENSGACG00000015416 | *novel gene* |  |
| 4.1 | ENSGACG00000019519 | *solute carrier family 38, member 4* | *slc38a4* |
| 7.2 | ENSGACG00000019411 | *G protein-coupled receptor 78* | *GPR78* |
| 7.2 | ENSGACG00000019414 | *carboxypeptidase Z* | *CPZ (2 of 2)* |
| 7.2 | ENSGACG00000019415 | *H6 family homeobox 4* | *hmx4 (2 of 2)* |
| 7.2 | ENSGACG00000019416 | *novel gene* |  |
| 7.2 | ENSGACG00000019417 | *adrenoceptor alpha 2C* | *ADRA2C (2 of 2)* |
| 7.2 | ENSGACG00000019418 | *docking protein 7* | *DOK7 (2 of 2)* |
| 7.2 | ENSGACG00000019423 | *HGF activator* | *HGFAC* |
| 7.2 | ENSGACG00000019425 | *regulator of G-protein signaling 12a* | *rgs12a* |
| 7.2 | ENSGACG00000019426 | *Myb/SANT-like DNA-binding domain containing 1* | *MSANTD1* |
| 7.2 | ENSGACG00000019427 | *deltex 4, E3 ubiquitin ligase* | *DTX4 (2 of 2)* |
| 7.2 | ENSGACG00000019428 | *si:ch211-119e14.1* | *si:ch211-119e14.1* |
| 7.2 | ENSGACG00000019429 | *coronin, actin binding protein, 1B* | *coro1b* |
| 7.2 | ENSGACG00000019430 | *claudin domain containing 1a* | *cldnd1a* |
| 7.2 | ENSGACG00000019431 | *novel gene* |  |
| 7.2 | ENSGACG00000019432 | *novel gene* |  |
| 7.2 | ENSGACG00000019433 | *novel gene* |  |
| 7.5 | ENSGACG00000019618 | *La ribonucleoprotein domain family, member 7* | *larp7* |
| 7.5 | ENSGACG00000019629 | *ADAMTS-like 1* | *ADAMTSL1 (2 of 2)* |
| 7.5 | ENSGACG00000019638 | *perilipin 2* | *plin2* |
| 7.5 | ENSGACG00000019649 | *cell division cycle associated 9* | *cdca9* |
| 7.5 | ENSGACG00000019650 | *DENN/MADD domain containing 4C* | *dennd4c* |
| 7.5 | ENSGACG00000019655 | *novel gene* |  |
| 7.5 | ENSGACG00000019656 | *HAUS augmin-like complex, subunit 6* | *haus6* |
| 7.5 | ENSGACG00000019658 | *phospholipase A2-activating protein* | *plaa* |
| 7.5 | ENSGACG00000019672 | *zinc finger, DHHC-type containing 21* | *ZDHHC21* |
| 7.5 | ENSGACG00000019677 | *nuclear factor I/B* | *NFIB* |
| 7.5 | ENSGACG00000019686 | *B-cell scaffold protein with ankyrin repeats 1* | *BANK1* |
| 7.5 | ENSGACG00000019688 | *protein phosphatase 3, catalytic subunit, alpha isozyme* | *PPP3CA* |
| 7.5 | ENSGACG00000019699 | *novel gene* |  |
| 7.5 | ENSGACG00000019700 | *DNA-damage-inducible transcript 4-like* | *DDIT4L* |
| 7.5 | ENSGACG00000019701 | *sperm associated antigen 17* | *SPAG17* |
| 7.5 | ENSGACG00000019708 | *transmembrane protein 151A* | *tmem151a* |
| 7.5 | ENSGACG00000022860 | *Small Cajal body specific RNA 8* | *SCARNA8* |
| 7.5 | ENSGACG00000022883 | *Small Cajal body specific RNA 8* | *SCARNA8* |
| 7.6 | ENSGACG00000019710 | *pyruvate carboxylase b* | *pcxb* |
| 7.6 | ENSGACG00000019743 | *leucine rich repeat and fibronectin type III domain containing 4a* | *lrfn4a* |
| 7.6 | ENSGACG00000019745 | *solute carrier family 3 (amino acid transporter heavy chain), member 2a* | *slc3a2a* |
| 7.6 | ENSGACG00000019747 | *sorting nexin 15* | *snx15* |
| 7.6 | ENSGACG00000019750 | *prospero homeobox 1b* | *prox1b* |
| 7.6 | ENSGACG00000019751 | *transmembrane protein 179B* | *TMEM179B* |
| 7.6 | ENSGACG00000019753 | *breast cancer metastasis suppressor 1* | *brms1* |
| 7.6 | ENSGACG00000019757 | *novel gene* |  |
| 7.6 | ENSGACG00000019758 | *protein phosphatase 1, regulatory (inhibitor) subunit 14Bb* | *ppp1r14bb* |
| 7.6 | ENSGACG00000019760 | *phospholipase C, beta 3 (phosphatidylinositol-specific)* | *plcb3* |
| 7.6 | ENSGACG00000019763 | *novel gene* |  |
| 7.6 | ENSGACG00000019764 | *solute carrier family 8 (sodium/calcium exchanger), member 4b* | *slc8a4b* |
| 7.6 | ENSGACG00000019768 | *BCL2-associated agonist of cell death b* | *badb* |
| 7.6 | ENSGACG00000019770 | *novel gene* |  |
| 7.6 | ENSGACG00000019790 | *novel gene* |  |
| 7.6 | ENSGACG00000019794 | *novel gene* |  |
| 7.6 | ENSGACG00000019799 | *glycoprotein hormone alpha 2* | *gpha2* |
| 7.6 | ENSGACG00000019802 | *glycoprotein hormone beta 5* | *gphb5* |
| 7.6 | ENSGACG00000019803 | *protein phosphatase 2, regulatory subunit B', beta* | *PPP2R5B* |
| 7.6 | ENSGACG00000019812 | *ADP-ribosylation factor-like 2* | *arl2* |
| 7.6 | ENSGACG00000019819 | *novel gene* |  |
| 7.6 | ENSGACG00000019824 | *beta-1,3-glucuronyltransferase 3 (glucuronosyltransferase I)* | *b3gat3* |
| 7.6 | ENSGACG00000019828 | *N(alpha)-acetyltransferase 40, NatD catalytic subunit, homolog (S. cerevisiae)* | *naa40* |
| 7.6 | ENSGACG00000019834 | *REST corepressor 2* | *rcor2* |
| 7.6 | ENSGACG00000019840 | *MAP/microtubule affinity-regulating kinase 2b* | *mark2b* |
| 7.6 | ENSGACG00000019844 | *kelch repeat-containing protein* | *krcp* |
| 7.6 | ENSGACG00000019847 | *chaperonin containing TCP1, subunit 7 (eta)* | *cct7* |
| 7.6 | ENSGACG00000022330 | *Small nucleolar RNA SNORD31* | *SNORD31* |
| 7.6 | ENSGACG00000022377 | *Small nucleolar RNA SNORD31* | *SNORD31* |
| 7.6 | ENSGACG00000022390 | *Small nucleolar RNA SNORD31* | *SNORD31* |
| 7.6 | ENSGACG00000022420 | *Small nucleolar RNA SNORD31* | *SNORD31* |
| 7.6 | ENSGACG00000022446 | *Small nucleolar RNA SNORD31* | *SNORD31* |
| 7.6 | ENSGACG00000022602 | *Small nucleolar RNA SNORD29* | *SNORD29* |
| 7.6 | ENSGACG00000022650 | *Small nucleolar RNA SNORD29* | *SNORD29* |
| 7.6 | ENSGACG00000022678 | *Small nucleolar RNA SNORD29* | *SNORD29* |
| 7.6 | ENSGACG00000022711 | *Small nucleolar RNA SNORD22* | *SNORD22* |
| 7.6 | ENSGACG00000022730 | *Small nucleolar RNA SNORD31* | *SNORD31* |
| 7.6 | ENSGACG00000022735 | *Small nucleolar RNA SNORD22* | *SNORD22* |
| 7.6 | ENSGACG00000022786 | *Small nucleolar RNA SNORD22* | *SNORD22* |
| 7.6,7.7 | ENSGACG00000019861 | *heat shock protein 12B* | *hspa12b* |
| 7.7 | ENSGACG00000019867 | *poly(A) binding protein interacting protein 2B* | *paip2b* |
| 7.7 | ENSGACG00000019870 | *N-acetylglucosamine kinase* | *nagk* |
| 7.7 | ENSGACG00000019876 | *docking protein 1b* | *dok1b* |
| 7.7 | ENSGACG00000019877 | *meiosis 1 associated protein* | *m1ap* |
| 7.8 | ENSGACG00000019881 | *sema domain, immunoglobulin domain (Ig), transmembrane domain (TM) and short cytoplasmic domain, (semaphorin) 4F* | *SEMA4F* |
| 7.8 | ENSGACG00000019883 | *novel gene* |  |
| 7.9 | ENSGACG00000019922 | *ephrin-B3b* | *efnb3b* |
| 7.9 | ENSGACG00000019923 | *WD repeat containing, antisense to TP53* | *wrap53 (1 of 2)* |
| 7.9 | ENSGACG00000019924 | *WD repeat containing, antisense to TP53* | *wrap53 (2 of 2)* |
| 7.9 | ENSGACG00000019925 | *GRB10 interacting GYF protein 1* | *GIGYF1* |
| 7.9 | ENSGACG00000019927 | *endonuclease domain containing 1* | *ENDOD1 (4 of 5)* |
| 7.9 | ENSGACG00000019928 | *UFM1-specific peptidase 1 (non-functional)* | *ufsp1* |
| 7.9 | ENSGACG00000019930 | *erythropoietin* | *epo* |
| 7.9 | ENSGACG00000019931 | *POP7 homolog, ribonuclease P/MRP subunit* | *pop7* |
| 7.9 | ENSGACG00000019933 | *novel gene* |  |
| 7.9 | ENSGACG00000019935 | *neuroligin 2a* | *nlgn2a* |
| 7.9 | ENSGACG00000019937 | *transmembrane protein 256* | *tmem256* |
| 7.9 | ENSGACG00000019941 | *transmembrane protein 102* | *TMEM102* |
| 7.9 | ENSGACG00000019942 | *fibroblast growth factor 11a* | *fgf11a* |
| 7.9 | ENSGACG00000019943 | *cholinergic receptor, nicotinic, beta 1 (muscle)* | *chrnb1* |
| 7.9 | ENSGACG00000019946 | *cholinergic receptor, nicotinic, beta 1 (muscle) like* | *chrnb1l* |
| 7.9 | ENSGACG00000019947 | *claudin 7a* | *cldn7a* |
| 7.9 | ENSGACG00000019950 | *novel gene* |  |
| 7.9 | ENSGACG00000019951 | *plac8 onzin related protein 5* | *ponzr5* |
| 7.9 | ENSGACG00000019952 | *novel gene* |  |
| 7.9 | ENSGACG00000019953 | *UDP glucuronosyltransferase 5 family, polypeptide G1* | *ugt5g1 (1 of 2)* |
| 7.9 | ENSGACG00000019954 | *UDP glucuronosyltransferase 5 family, polypeptide G1* | *ugt5g1 (2 of 2)* |
| 7.9 | ENSGACG00000019958 | *novel gene* |  |
| 7.9 | ENSGACG00000019959 | *prostaglandin D2 receptor 2* | *PTGDR2 (2 of 3)* |
| 7.9 | ENSGACG00000019960 | *prostaglandin D2 receptor 2* | *PTGDR2 (3 of 3)* |
| 7.9 | ENSGACG00000019961 | *novel immune-type receptor 9* | *nitr9 (1 of 12)* |
| 7.9 | ENSGACG00000019963 | *novel immune-type receptor 9* | *nitr9 (2 of 12)* |
| 7.9 | ENSGACG00000019964 | *novel immune-type receptor 9* | *nitr9 (3 of 12)* |
| 7.9 | ENSGACG00000019965 | *novel immune-type receptor 9* | *nitr9 (4 of 12)* |
| 7.9 | ENSGACG00000019966 | *novel immune-type receptor 9* | *nitr9 (5 of 12)* |
| 7.9 | ENSGACG00000019968 | *novel immune-type receptor 9* | *nitr9 (6 of 12)* |
| 7.9 | ENSGACG00000019969 | *novel immune-type receptor 9* | *nitr9 (7 of 12)* |
| 7.9 | ENSGACG00000019971 | *novel immune-type receptor 9* | *nitr9 (8 of 12)* |
| 7.9 | ENSGACG00000019972 | *novel immune-type receptor 9* | *nitr9 (9 of 12)* |
| 7.9 | ENSGACG00000019974 | *novel immune-type receptor 9* | *nitr9 (10 of 12)* |
| 7.9 | ENSGACG00000019976 | *novel immune-type receptor 9* | *nitr9 (11 of 12)* |
| 7.9 | ENSGACG00000019978 | *novel immune-type receptor 9* | *nitr9 (12 of 12)* |
| 7.9 | ENSGACG00000019980 | *RNA binding motif protein 4.3* | *rbm4.3* |
| 7.9 | ENSGACG00000019982 | *TRAF-interacting protein with forkhead-associated domain* | *TIFA* |
| 7.9 | ENSGACG00000019983 | *fragile X mental retardation, autosomal homolog 2* | *fxr2* |
| 7.10 | ENSGACG00000020028 | *novel gene* |  |
| 7.10 | ENSGACG00000020029 | *novel gene* |  |
| 7.10 | ENSGACG00000020030 | *neurexin 2* | *NRXN2* |
| 7.11 | ENSGACG00000020035 | *DR1-associated protein 1 (negative cofactor 2 alpha)* | *drap1* |
| 7.11 | ENSGACG00000020038 | *chromosome 11 open reading frame 68* | *C11orf68* |
| 7.11 | ENSGACG00000020039 | *v-rel avian reticuloendotheliosis viral oncogene homolog A* | *rela* |
| 7.11 | ENSGACG00000020043 | *protein phosphatase 1, catalytic subunit, beta isoform, like* | *ppp1cbl* |
| 7.11 | ENSGACG00000020057 | *solute carrier family 29 (equilibrative nucleoside transporter), member 2* | *SLC29A2 (2 of 2)* |
| 7.12 | ENSGACG00000000797 | *potassium channel, two pore domain subfamily K, member 7* | *KCNK7* |
| 7.12 | ENSGACG00000000799 | *TAF6-like RNA polymerase II, p300/CBP-associated factor (PCAF)-associated factor* | *taf6l* |
| 7.12 | ENSGACG00000000802 | *cth1* | *cth1* |
| 7.12 | ENSGACG00000000803 | *metastasis associated 1 family, member 2* | *mta2* |
| 7.12 | ENSGACG00000000804 | *echinoderm microtubule associated protein like 3* | *eml3* |
| 7.12 | ENSGACG00000000808 | *novel gene* |  |
| 7.12 | ENSGACG00000000809 | *ECSIT signalling integrator* | *ecsit* |
| 7.12 | ENSGACG00000000811 | *si:dkey-28a3.2* | *si:dkey-28a3.2* |
| 7.12 | ENSGACG00000000812 | *reticulon 3* | *rtn3* |
| 7.12 | ENSGACG00000000817 | *multiple endocrine neoplasia I* | *men1* |
| 7.12 | ENSGACG00000000819 | *mitogen-activated protein kinase kinase kinase kinase 2* | *map4k2* |
| 7.12 | ENSGACG00000000821 | *RNA binding motif protein 4.2* | *rbm4.2* |
| 7.12 | ENSGACG00000000822 | *RNA binding motif protein 4.1* | *rbm4.1* |
| 7.12 | ENSGACG00000000826 | *splicing factor 1* | *sf1* |
| 7.12 | ENSGACG00000000827 | *phosphorylase, glycogen, muscle b* | *pygmb* |
| 7.12 | ENSGACG00000000840 | *novel gene* |  |
| 7.12 | ENSGACG00000000843 | *bolA family member 2B* | *BOLA2B* |
| 7.12 | ENSGACG00000000846 | *SLX1 structure-specific endonuclease subunit homolog B (S. cerevisiae)* | *slx1b* |
| 7.12 | ENSGACG00000000848 | *im:7136729* | *im:7136729* |
| 7.12 | ENSGACG00000000850 | *CD68 molecule* | *CD68* |
| 7.12 | ENSGACG00000000852 | *inhibitor of growth family, member 2* | *ing2* |
| 7.12 | ENSGACG00000000853 | *RWD domain containing 4* | *rwdd* |
| 7.12 | ENSGACG00000000862 | *ADAM metallopeptidase domain 19a* | *adam19a* |
| 7.12 | ENSGACG00000000867 | *leucine-rich repeats and calponin homology (CH) domain containing 4* | *lrch4* |
| 7.12 | ENSGACG00000000870 | *H2A histone family, member Z* | *H2AFZ* |
| 7.12 | ENSGACG00000000874 | *myeloid/lymphoid or mixed-lineage leukemia (trithorax homolog, Drosophila); translocated to, 3* | *mllt3* |
| 7.12 | ENSGACG00000000877 | *focadhesin* | *focad* |
| 7.12 | ENSGACG00000000890 | *3-hydroxyacyl-CoA dehydratase 4* | *HACD4* |
| 7.12 | ENSGACG00000000892 | *tRNA methyltransferase 10 homolog A (S. cerevisiae)* | *TRMT10A* |
| 7.12 | ENSGACG00000000900 | *novel gene* |  |
| 7.12 | ENSGACG00000000901 | *solute carrier family 1 (neuronal/epithelial high affinity glutamate transporter, system Xag), member 1* | *slc1a1* |
| 7.12 | ENSGACG00000000917 | *RAP1, GTP-GDP dissociation stimulator 1* | *RAP1GDS1* |
| 7.12 | ENSGACG00000000929 | *tetraspanin 5a* | *tspan5a* |
| 7.12 | ENSGACG00000000932 | *Fras1 related extracellular matrix 1a* | *frem1a* |
| 7.12 | ENSGACG00000000941 | *sortilin-related VPS10 domain containing receptor 2* | *SORCS2* |
| 7.12 | ENSGACG00000000958 | *GrpE-like 1, mitochondrial* | *grpel1* |
| 7.12 | ENSGACG00000000960 | *transcriptional adaptor 2B* | *tada2b* |
| 7.12 | ENSGACG00000000961 | *actin filament associated protein 1* | *afap1* |
| 7.12 | ENSGACG00000000966 | *actin binding LIM protein family, member 2* | *ablim2* |
| 7.12 | ENSGACG00000022460 | *novel gene* |  |
| 7.13 | ENSGACG00000020129 | *roundabout, axon guidance receptor, homolog 3 (Drosophila)* | *robo3* |
| 7.13 | ENSGACG00000020133 | *Myb/SANT-like DNA-binding domain containing 2* | *MSANTD2* |
| 7.14 | ENSGACG00000020188 | *Down syndrome cell adhesion molecule* | *DSCAM (2 of 2)* |
| 9.4 | ENSGACG00000018376 | *HtrA serine peptidase 3a* | *htra3a* |
| 9.4 | ENSGACG00000018377 | *phosphatidylinositol 4-kinase type 2 beta* | *pi4k2b* |
| 9.4 | ENSGACG00000018380 | *ring finger protein 38* | *rnf38 (2 of 2)* |
| 9.4 | ENSGACG00000018382 | *cholinergic receptor, nicotinic, beta 2a* | *chrnb2a* |
| 9.4 | ENSGACG00000018392 | *cholinergic receptor, nicotinic, alpha 6* | *chrna6 (2 of 2)* |
| 9.4 | ENSGACG00000018396 | *cholinergic receptor, nicotinic, beta polypeptide 3a* | *chrnb3a* |
| 9.4 | ENSGACG00000018398 | *si:ch73-380n15.2* | *si:ch73-380n15.2 (2 of 2)* |
| 9.4 | ENSGACG00000018400 | *dynein, axonemal, heavy chain 11* | *dnah11* |
| 12.3 | ENSGACG00000011124 | *spalt-like transcription factor 4* | *sall4* |
| 12.3 | ENSGACG00000011128 | *zinc finger protein 64 homolog (mouse)* | *zfp64* |
| 12.3 | ENSGACG00000011135 | *teashirt zinc finger homeobox 2* | *TSHZ2* |
| 12.3 | ENSGACG00000011137 | *novel gene* |  |
| 12.3 | ENSGACG00000011139 | *prefoldin subunit 4* | *pfdn4* |
| 12.3 | ENSGACG00000011148 | *novel gene* |  |
| 12.3 | ENSGACG00000011149 | *cerebellin 4 precursor* | *cbln4* |
| 12.3 | ENSGACG00000011155 | *CD40 molecule, TNF receptor superfamily member 5* | *cd40* |
| 12.3 | ENSGACG00000011160 | *iroquois homeobox 7* | *irx7* |
| 12.3 | ENSGACG00000011163 | *novel gene* |  |
| 12.3 | ENSGACG00000011168 | *pleckstrin homology domain containing, family G (with RhoGef domain) member 5* | *PLEKHG5* |
| 12.3 | ENSGACG00000011178 | *nucleolar protein 9* | *nol9* |
| 12.3 | ENSGACG00000011186 | *novel gene* |  |
| 12.3 | ENSGACG00000011187 | *zinc finger and BTB domain containing 48* | *zbtb48* |
| 12.3 | ENSGACG00000011193 | *kelch-like family member 21* | *klhl21* |
| 12.3 | ENSGACG00000011194 | *synaptotagmin VIb* | *syt6b* |
| 12.3 | ENSGACG00000011204 | *olfactomedin-like 3a* | *olfml3a* |
| 12.3 | ENSGACG00000011209 | *family with sequence similarity 132, member A* | *fam132a* |
| 12.3 | ENSGACG00000011217 | *stromal cell derived factor 4* | *sdf4* |
| 12.3 | ENSGACG00000011240 | *homeodomain interacting protein kinase 1a* | *hipk1a* |
| 12.5 | ENSGACG00000010722 | *PAN2 poly(A) specific ribonuclease subunit homolog (S. cerevisiae)* | *pan2* |
| 12.5 | ENSGACG00000010743 | *ORMDL sphingolipid biosynthesis regulator 2* | *ormdl2* |
| 12.5 | ENSGACG00000010747 | *nuclear envelope integral membrane protein 1* | *nemp1* |
| 12.5 | ENSGACG00000010752 | *CD63 molecule* | *cd63* |
| 12.5 | ENSGACG00000010770 | *LETM1 domain containing 1* | *letmd1* |
| 12.5 | ENSGACG00000010783 | *zgc:56699* | *zgc:56699* |
| 12.5 | ENSGACG00000010788 | *nuclear receptor subfamily 4, group A, member 1* | *NR4A1 (2 of 2)* |
| 12.5 | ENSGACG00000010800 | *GRP1 (general receptor for phosphoinositides 1)-associated scaffold protein* | *grasp* |
| 12.5 | ENSGACG00000010810 | *diacylglycerol kinase, alpha a* | *dgkaa* |
| 12.5 | ENSGACG00000010838 | *ring finger protein 41* | *rnf41* |
| 12.5 | ENSGACG00000010842 | *ankyrin repeat domain 52a* | *ankrd52a* |
| 12.5 | ENSGACG00000010849 | *coenzyme Q10A* | *COQ10A* |
| 12.5 | ENSGACG00000010851 | *citrate synthase* | *cs* |
| 12.5 | ENSGACG00000010877 | *novel gene* |  |
| 12.5 | ENSGACG00000010885 | *si:ch211-210c8.6* | *si:ch211-210c8.6* |
| 12.5 | ENSGACG00000010890 | *novel gene* |  |
| 12.5 | ENSGACG00000010902 | *si:ch211-210c8.7* | *si:ch211-210c8.7* |
| 12.5 | ENSGACG00000010906 | *DAZ associated protein 2* | *dazap2* |
| 12.5 | ENSGACG00000010925 | *POU class 6 homeobox 1* | *pou6f1* |
| 12.5 | ENSGACG00000010929 | *transcription factor CP2* | *tfcp2* |
| 12.5 | ENSGACG00000010943 | *cysteine-serine-rich nuclear protein 2* | *csrnp2* |
| 12.5 | ENSGACG00000010945 | *integrin, alpha 5 (fibronectin receptor, alpha polypeptide)* | *itga5* |
| 12.5 | ENSGACG00000010990 | *Rap guanine nucleotide exchange factor (GEF) 3* | *rapgef3* |
| 12.5 | ENSGACG00000011000 | *solute carrier family 26 (anion exchanger), member 9* | *SLC26A9* |
| 12.5 | ENSGACG00000011005 | *si:ch211-226m16.3* | *si:ch211-226m16.3* |
| 12.5 | ENSGACG00000021334 | *novel gene* |  |
